# Illumina next generation sequencing data and expression microarrays data from retinoblastoma and medulloblastoma tissues

**DOI:** 10.1016/j.dib.2015.12.052

**Published:** 2016-01-27

**Authors:** A.J. García-Chequer, A. Méndez-Tenorio, G. Olguín-López, C. Sánchez-Vallejo, P. Isa, C.F. Arias, J. Torres, A. Hernández-Angeles, M.A. Ramírez-Ortiz, C. Lara, Ma.de.L. Cabrera-Muñoz, S. Sadowinski-Pine, J.C. Bravo-Ortiz, G. Ramón-García, J. Diegopérez-Ramírez, G. Ramírez-Reyes, R. Casarrubias-Islas, J. Ramírez, M. Orjuela, M.V. Ponce-Castañeda

**Affiliations:** a Unidad de Investigación Médica en Enfermedades Infecciosas, Hospital de Pediatría, Instituto Mexicano del Seguro Social, Centro Médico Nacional SXXI, México D.F., Mexico; b Escuela Nacional de Ciencias Biológicas, Instituto Politécnico Nacional, México D.F., Mexico; c Instituto de Biotecnología, Universidad Nacional Autónoma de México, Cuernavaca, Mexico; d Hospital Infantil de México Federico Gómez, México D.F., Mexico; e Columbia University, New York, USA; f Unidad de Microarreglos, Instituto de Fisiología Celular, Universidad Nacional Autónoma de México, México D.F., Mexico

## Abstract

Retinoblastoma (Rb) is a pediatric intraocular malignancy and probably the most robust clinical model on which genetic predisposition to develop cancer has been demonstrated. Since deletions in chromosome 13 have been described in this tumor, we performed next generation sequencing to test whether recurrent losses could be detected in low coverage data. We used Illumina platform for 13 tumor tissue samples: two pools of 4 retinoblastoma cases each and one pool of 5 medulloblastoma cases (raw data can be found at http://www.ebi.ac.uk/ena/data/view/PRJEB6630).

We first created an *in silico* reference profile generated from a human sequenced genome (GRCh37p5). From this data we calculated an integrity score to get an overview of gains and losses in all chromosomes; we next analyzed each chromosome in windows of 40 kb length, calculating for each window the log_2_ ratio between reads from tumor pool and *in silico* reference. Finally we generated panoramic maps with all the windows whether lost or gained along each chromosome associated to its cytogenetic bands to facilitate interpretation. Expression microarrays was done for the same samples and a list of over and under expressed genes is presented here. For this detection a significance analysis was done and a log_2_ fold change was chosen as significant (raw data can be found at http://www.ncbi.nlm.nih.gov/geo/accession number GSE11488). The complete research article can be found at Cancer Genetics journal (Garcia-Chequer et al., in press) [1]. In summary here we provide an overview with visual graphics of gains and losses chromosome by chromosome in retinoblastoma and medulloblastoma, also the integrity score analysis and a list of genes with relevant expression associated. This material can be useful to researchers that may want to explore gains and losses in other malignant tumors with this approach or compare their data with retinoblastoma.

## **Specifications Table**

**Table:** 

Subject area	Biomedicine, Bioinformatics
More specific subject area	Human Genomics of retinoblastoma and medulloblastoma.
Type of data	Tables, graphs, chromosome maps and figures
Organism/cell line/tissue	Human, retinoblastoma and medulloblastoma primary tissues
How data was acquired	DNA was isolated from snap frozen pediatric tumors (retinoblastoma and medulloblastoma), DNA was pooled and sequenced using an Illumina Genome Analyzer IIX. Expression microarray data was generated with RNA isolated from the corresponding short term primary cultures and analyzed with an in house two channel platform of expression microarrays and using as a reference RNA isolated from *S. cerevisiae* harvested at log phase growth.
Data format	Raw sequencing data, analyzed data and mapped data
Experimental factors	2 retinoblastoma pools (2 boys and 2 girls each one)
1 medulloblastoma pool (4 boys and 1 girl)
All patients were naïve to chemotherapy treatment at the moment of the sample collection.
Experimental features	Retinoblastoma and medulloblastoma tissues samples were deep sequenced in 3 pools (2 Rb and 1 Mb). The sequencing data was filtered, mapped using an *in silico* reference and analyzed chromosome by chromosome to evaluate their integrity and to ponder important regions of gains or losses at the chromosomes.
Consent	All tumor tissues were collected under informed written consent from the patients’ parents and as part of studies approved by the Scientific and Ethics Review Boards from each participating institution.
Data source location	Mexico city, Hospital de Pediatría, Centro Médico Nacional Siglo XXI, IMSS and Mexico city, Hospital Infantil de México, SS.
Data accessibility	Deposited raw data of the NGS of retinoblastoma and meduloblastoma can be found at: http://www.ebi.ac.uk/ena/data/view/PRJEB6630.
Microarray Data sets are uploaded at GEO-NCBI database in: http://www.ncbi.nlm.nih.gov/geo/ accession number GSE11488.

## **Value of the data**

• Retinoblastoma tissues naïve to treatment are very rare and scarce and thus difficult to obtain and study.
• With the visual graphics presented, the data provides an overview of the retinoblastoma and medulloblastoma genomes structure easy to comprehend, analyze and compare.
• This data facilitates the merge of cytogenetic information and vocabulary with next generation sequencing information and vocabulary.
• The format of data presentation with the pooling experimental design facilitates navigation through complex data, allowing exploration of recurrent events in an intuitive manner.
• Simplicity of these analyses can guide researchers about interesting genomic areas of consistent recurrent losses for further study in these genomes.
• The data can help in the design of future analysis like cytogenetics, selecting genes to study by immunohistochemistry, gene hunting etc.
• The data can help to easily compare chromosomal changes with other type of tumors.

## 1 Data

Next generation sequencing was done for retinoblastoma and medulloblastoma samples. These sequences or “reads” were mapped and compared to a human reference genome and low coverage was determined. This comparison allowed the construction of chromosomal maps with positions and association to cytogenetic bands for an overview of the data. An integrity score was used for a panoramic view of the sequencing data. Expression microarray was also done for the same samples and lists of over and under expressed genes are presented related to regions of gains and losses [1].

## 2 Experimental design

Tumor tissue samples from 8 retinoblastoma patients and from 5 medulloblastoma patients were collected. These 13 samples were deep sequenced as three pools, two for retinoblastoma and one for medulloblastoma. The *in silico* references were generated from a whole human genome sequence GRCh37p5 from the Genome Reference Consortium.

## 3 Materials and methods

### 3.1 Patients and DNA extraction

Eight patients diagnosed with retinoblastoma (Rb) and five with medulloblastoma (Mb) were included. DNA was isolated from snap frozen tumor tissues. Two DNA pools from four Rb patients each were considered biological replicas and one DNA pool containing five Mb patients considered a biological contrast were sequenced. The original and raw data are uploaded at the European Nucleotide Archive and can be found in http://www.ebi.ac.uk/ena/data/view/PRJEB6630.

All patients were newly diagnosed and tumor samples were collected at time of surgery, prior to any adjuvant therapy. Tumor tissues were collected under informed written consent from their parents and as part of studies approved by the Scientific and Ethics Review Boards from each participating institution.

### 3.2 Illumina sequencing

Sequencing was performed at the sequencing core facility of the National University of Mexico (UNAM) located at the Biotechnology Institute in Cuernavaca, Mexico using the Illumina Genome Analyzer IIx (Illumina). For sequencing 5 µg of DNA, 1.2 μg DNA from each retinoblastoma per pool, and 1 µg DNA from each meduloblastoma per pool were used to make approximately two hundred base-pair sized libraries using Illumina’s Genomic DNA sample prep kit.

### 3.3 *In silico* genome references

A reference profile of sequencing data for each sample was obtained using an *in silico* technique. The whole human genome sequence GRCh37p5 was downloaded from the Genome Reference Consortium.

A random sampling of this reference genome was done for the generation of reference sets of reads.

### 3.4 Reads mapping and symmetric relation treatment

The treatment applied to both sequencing datasets and *in silico* datasets was the same. After filtering out low quality and low complexity reads, both *in silico* and experimental sets were then mapped to the GRCh37.p5 genome sequence with Bowtie2 [2]. A Perl program was written for extracting the reads that map only once to the Human Genome. For the comparison between the *in silico* reads and *in vitro* reads already mapped, log_2_ ratios values were used.

$$\log\;\mathrm{ratio}=\log_{2}\frac{\mathrm{Number}\;\mathrm{of}\;\mathrm{reads}\;\mathrm{in}\;\mathrm{Sample}\;X}{\mathrm{Number}\;\mathrm{of}\;\mathrm{reads}\;\mathrm{in}\;\mathrm{Referece}\;X}$$

The log_2_ ratio gives a symmetric relation between two sets of values facilitating the interpretation of the data. For example, a 2 value will be equivalent to a −2, that will correspond to a relation of 4:1 and 1:4 respectively or a fold change of 4.

### 3.5 ChromDraw

ChromDraw.exe is a computational program that draws each chromosome and all its cytogenetic bands. This graphic visualization software was written by one of the authors of the present work (Méndez-Tenorio, PhD) with the purpose of facilitating identification of losses and gains in each chromosome by analyzing the comparison between one sample and a reference using the log_2_ ratio value. This software is available upon request.

### 3.6 Integrity score

The integrity score was calculated as a relation between the number of reads in a chromosome and the length of that chromosome, in units of reads/kilobase. The integrity score allow us an overview of losses and gains in all the chromosomes. This score is calculated as follows:

$$\mathrm{Score}\;\mathrm{Chr}i=\frac{\mathrm{Number}\;\mathrm{of}\;\mathrm{reads}\;\mathrm{in}\;\mathrm{chromosome}\;i}{\mathrm{Length}\;\mathrm{of}\;\mathrm{chromosome}\;i}\left[=\right]\frac{\mathrm{reads}}{\mathrm{kB}}$$

**Fig. 1:**
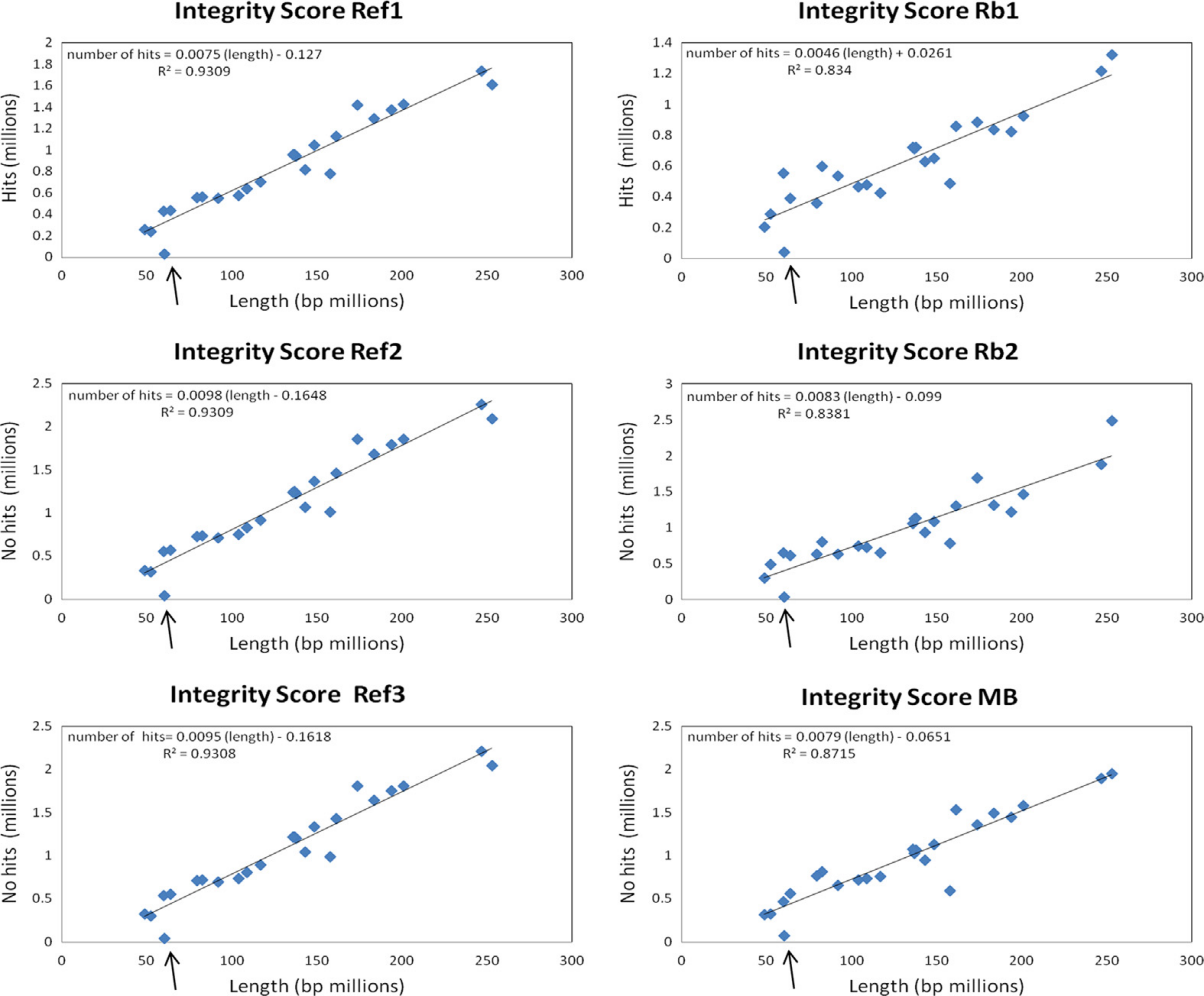
Integrity score for all the samples including ChrY. Each plot corresponds to each one of the samples, in the left the references and in the right the tumors. The points in the plots represent the human chromosomes. Chromosome Y is an outlier indicated by the arrow in all the samples.

**Fig. 2:**
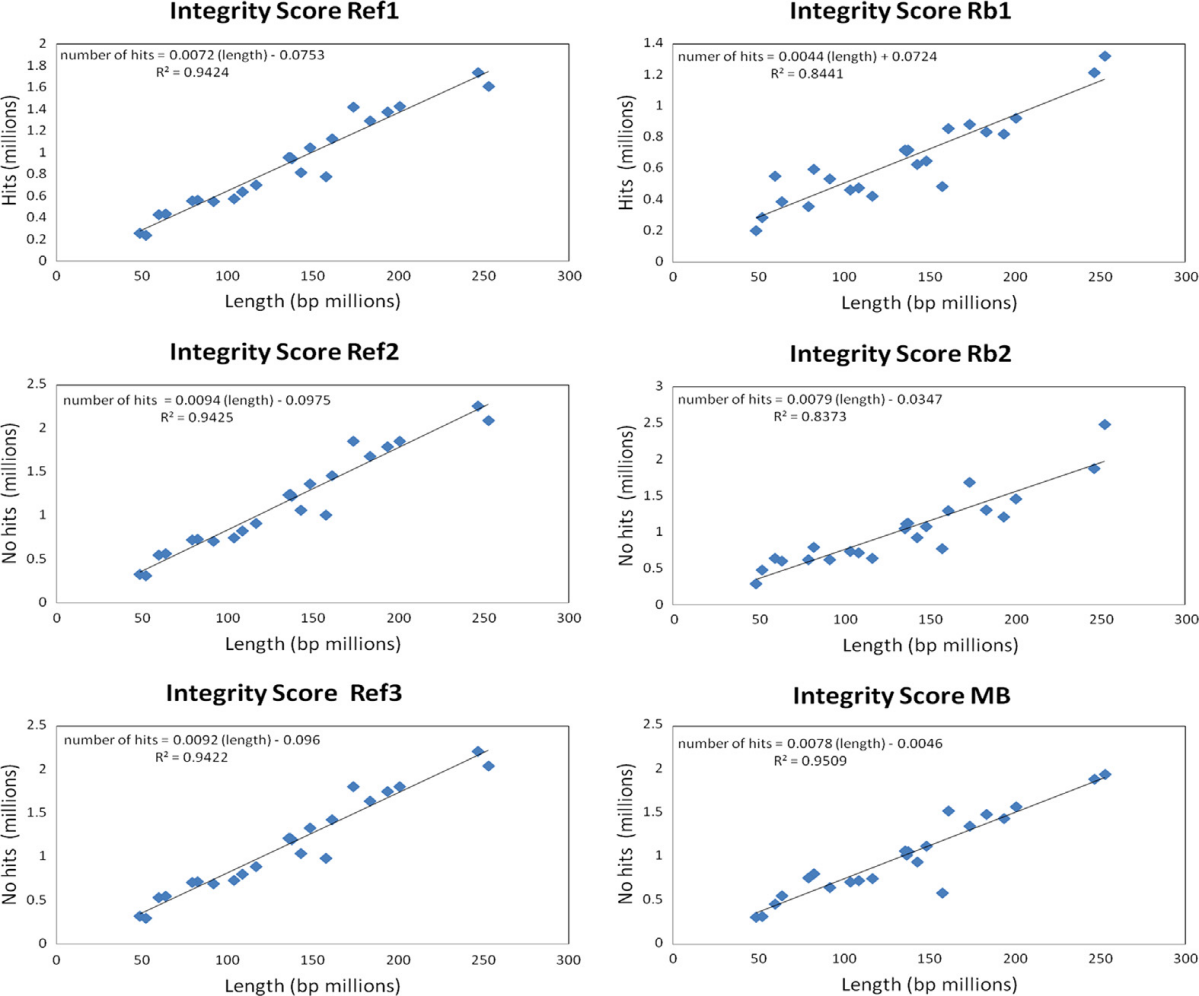
Integrity score for all the samples not including ChrY. Each plot corresponds to each one of the samples, in the left the references and in the right the tumors. The points in the plots represent the human chromosomes.

**Fig. 3:**
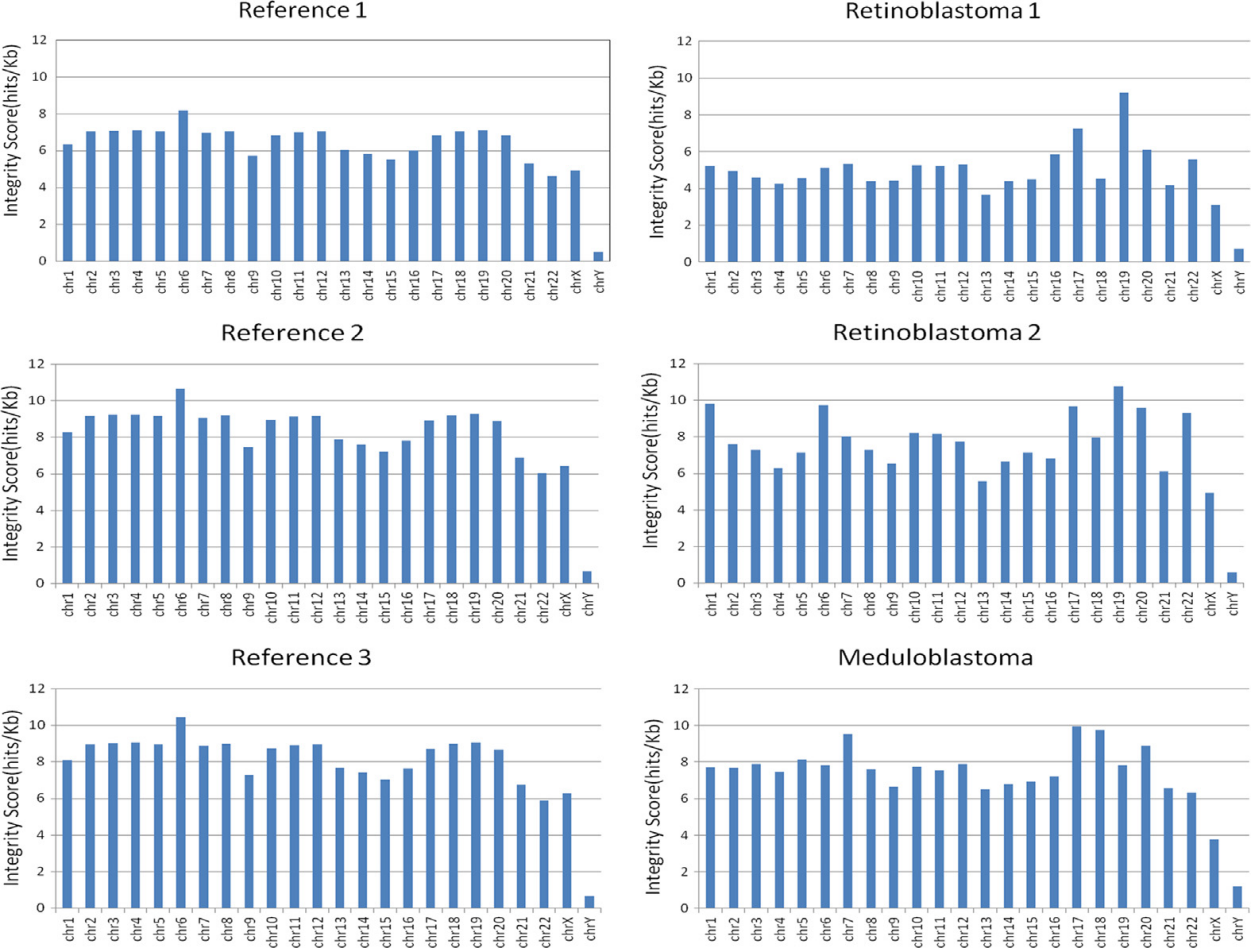
Integrity score plotted as bars. Each bar corresponds to each chromosome and this figure shows a marked difference for chromosome Y.

With this score is possible to explore linearity of sequencing and *in silico* data by plotting the size of each chromosome in millions of bp against the number of reads obtained for each chromosome. Because we found a linear relationship between filtered reads and chromosome size we developed the integrity score. Using this integrity score is possible to determine with low resolution if there is any chromosome with less or more reads compared to its reference. The integrity score was calculated for all the chromosomes in the six data samples, three for the reference samples and three for the pooled tumor samples. Fig. 1, Fig. 2 show the plot for all the references and samples considering or not the Chromosome Y respectively. The *r*^2^ value can be seen in each individual plot. Another figure shows the Integrity Score plotted as bars and a marked difference for chromosome Y is observed in all tumors and reference samples (Fig. 3).

### 3.7 Chromosomes analyses by windows

Each chromosome sequence was divided into fragments of the same length (40 kb) called ‘windows’. Mapped reads in each window for both the *in silico* reference and the tumor samples were measured and using ChromDraw data is plotted as log_g_ ratios against the cytogenetic maps (See Supplemental Maps File) (Table 1).

### 3.8 Expression microarrays

Expression microarray data was generated at the microarray facility of the National University of Mexico (UNAM) located at the Instituto de Fisiología Celular in Mexico City. Total RNA was obtained using Trizol reagent by Ambion according to manufacturer directions, from short term primary cultures established from enucleated eyes and propagated for seven days in RPMI 15% FBS. In house double channel platform was used and microarrays were printed with a human 50-mer oligo library set “A” from MWG BiotechOligo Gene expression was determined as significant over or under expressed using the Significance Analysis of Microarray (SAM) algorithm from the TM4 suite of programs [3]. A log fold change value larger than 2 or lower than -2 was used for selecting the over and under expresses genes respectively and a false discovery rate of 0.05 [4]. A list with the over and under expressed genes is shown in Table 2.

## Figures and Tables

**Table 1:** Shared gross losses between retinoblastoma and medulloblastoma.

Chromosome	Cytogenetic band	Loss location	# Genes
(loss size)
Chr 5	q13.2	68.5 M–71 M	66
(2.5 M)
Chr 6	p22.1	28.4M–33.4 M	407
p21.33	(5 M)
p21.32	
Chr 17	q21.31	44.1 M–44.6 M	28
(560 K)
Chr 19	p13.11	20 M–22.5 M	103
(2.5 M)
Chr 19	q13.42	54.5 M–55.5 M	66
(1.04 M)

**Table 2:** Over and underexpressed genes in retinoblastoma samples.

**CHR**	**Number of overexpressed genes**	**Symbol**	**log fold change**	**Number of underexpressed genes**	**Symbol**	**log fold change**
chr1	6	CSF3R	2.98	2	LOC376745	−2.59
		CDC42BPA	2.55		DDX20	−2.73
		FLJ20277	2.33			
		TRIM17	2.32			
		KIAA0663	2.27			
		WARP	2.26			
chr2	3	ZFHX1B	3.51	2	ANXA4	−2.68
		SEMA4F	2.96		TLK1	−2.71
		C2orf24	2.14			
chr3	5	FSTL1	2.83	0		
		SYN2	2.77			
		CCR1	2.28			
		BAP1	2.12			
		ZFYVE20	2.10			
chr4	5	FGFR3	2.43	1	FABP2	−2.61
		MXD4	2.40			
		STK32B	2.35			
		MAD2L1	2.20			
		LIM	2.12			
chr5	0			2	SNX2	−2.79
					PCSK1	−2.86
chr6	4	CUL7	2.75	6	AMD1	−2.13
		APOBEC2	2.70		TPMT	−2.33
		RXRB	2.52		BF	−2.48
		C6orf108	2.27		PGC	−2.53
					HIST1H1C	−2.74
					GOPC	−3.38
chr7	1	FLJ20359	2.77	2	ORC5L	−2.76
					ARP3BETA	−3.53
chr8	2	FLJ14299	2.84	2	IMPA1	−2.70
		LY6E	2.49		TNFRSF10A	−2.73
chr9	2	SARDH	2.63	2	C9orf78	−2.54
		GTF3C4	2.40		SMARCA2	−3.08
chr10	3	GDF2	2.36	2	SEC23IP	−2.20
		CCDC6	2.20		ACADSB	−2.67
		LIPA	2.13			
chr11	5	VEGFB	2.89	1	FLJ22794	−2.71
		ZBTB3	2.76			
		TIMM10	2.56			
		DKK3	2.42			
		MTA2	2.24			
chr12	7	NM_002261	3.30	3	ATP5B	−2.33
		NAB2	2.98		ING4	−2.63
		DKFZP586A0522	2.85		HCFC2	−2.87
		NM_025247	2.50			
		NM_014830	2.47			
		OAS1	2.42			
		OAS1	2.35			
chr13	0			0		
chr14	1	NKX2−8	2.75	0		
chr15	3	SMAD3	2.99	0		
		B7H3	2.62			
		FOXB1	2.34			
chr16	4	ALDOA	2.92	5	TRAP1	−2.33
		MT3	2.80		MRTF-B	−2.36
		SLC12A4	2.75		HMOX2	−2.39
		TRADD	2.46		KIAA0174	−2.47
chr17	7	FLJ11164	3.26	2	CCL7	−2.48
		HOXB4	3.01		TBCD	−2.61
		TOP3A	2.91			
		DKFZp434H2215	2.64			
		FLJ10385	2.62			
		MGC4172	2.54			
		MEOX1	2.24			
chr18	0			0		
chr19	13	SNAPC2	3.07	4	FLJ11535	−2.22
		TBXA2R	3.04		POLD1	−2.39
		P2RY11	3.00		ASNA1	−3.45
		MGC3169	2.86		GAPDS	−4.16
		AURKC	2.69			
		CEACAM3	2.56			
		ZNF42	2.37			
		EFNA2	2.36			
		VMD2L1	2.30			
		EPOR	2.27			
		MRPL4	2.26			
		KLF2	2.25			
		IL12RB1	2.08			
chr20	2	GNRH2	4.23	0		
		GPR8	3.12			
chr21	1	NDUFV3	2.86	1	CLDN17	−2.90
chr22	2	SUSD2	2.84	3	TOM1	−2.11
		XBP1	2.61		PSCD4	−2.24
chrX	1	CTAG1B	2.58	2	HEPH	−2.42
					DKC1	−2.50

## References

[1] A.J. Garcia-Chequer, A. Mendez-Tenorio, J. Torres, et al., Overview of recurrent chromosomal losses in retinoblastoma detected by low coverage next generation sequencing, Cancer Genet, 10.1016/j.cancergen.2015.12.001, in press, 2016 10.1016/j.cancergen.2015.12.001 PMC4993565 26883451

[2] Langmead B., Salzberg S.L. (2012). Fast gapped-read alignment with Bowtie 2. Nat. Methods.

[3] Saeed A.I., Sharov V., White J. (2003). TM4: a free, open-source system for microarray data management and analysis. Biotechniques.

[4] Storey J.D., Tibshirani R. (2003). Statistical methods for identifying differentially expressed genes in DNA microarrays. Methods Mol. Biol..

