# Supplementary material for: Illumina next generation sequencing data and expression microarrays data from retinoblastoma and medulloblastoma tissues
Source: Data Brief. 2016 Jan 27;6:908–16. doi: 10.1016/j.dib.2015.12.052 (PMC4753385; doi:10.1016/j.dib.2015.12.052)
Supplement: Supplementary file 1 — Supplementary material [file mmc1.pdf]

Chromosome: chr1
Length: 249250621

Number of RefSeq genes: 3511
Number of genes on positive strand: 1797
Number of genes on negative strand: 1714

Chr1 Mb pool

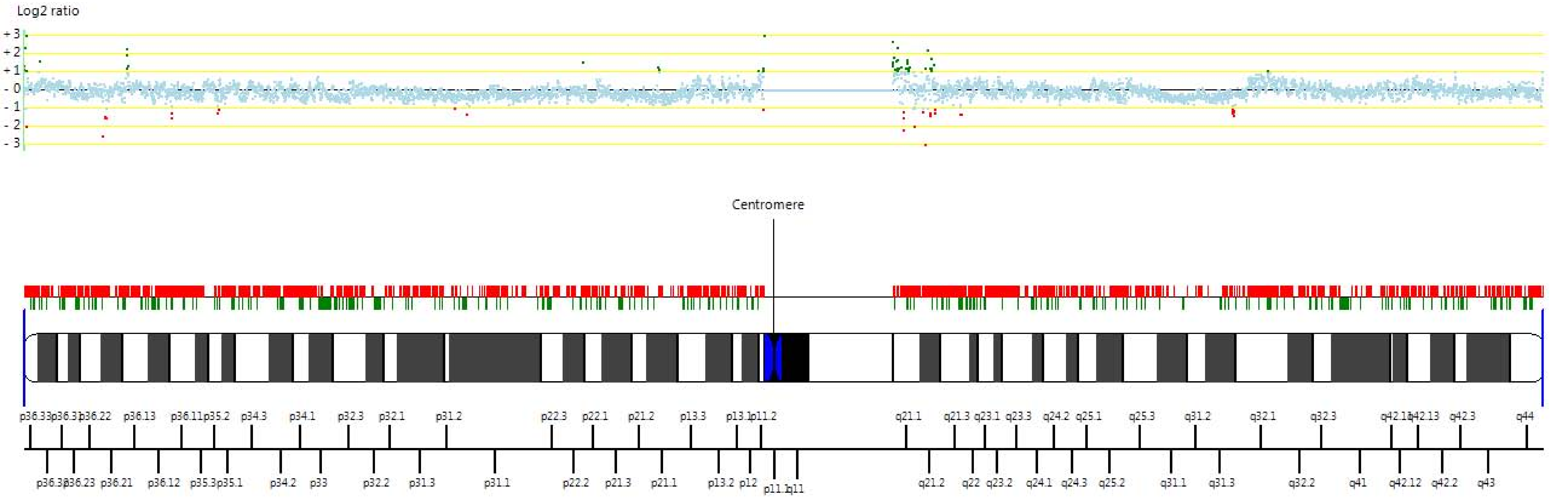

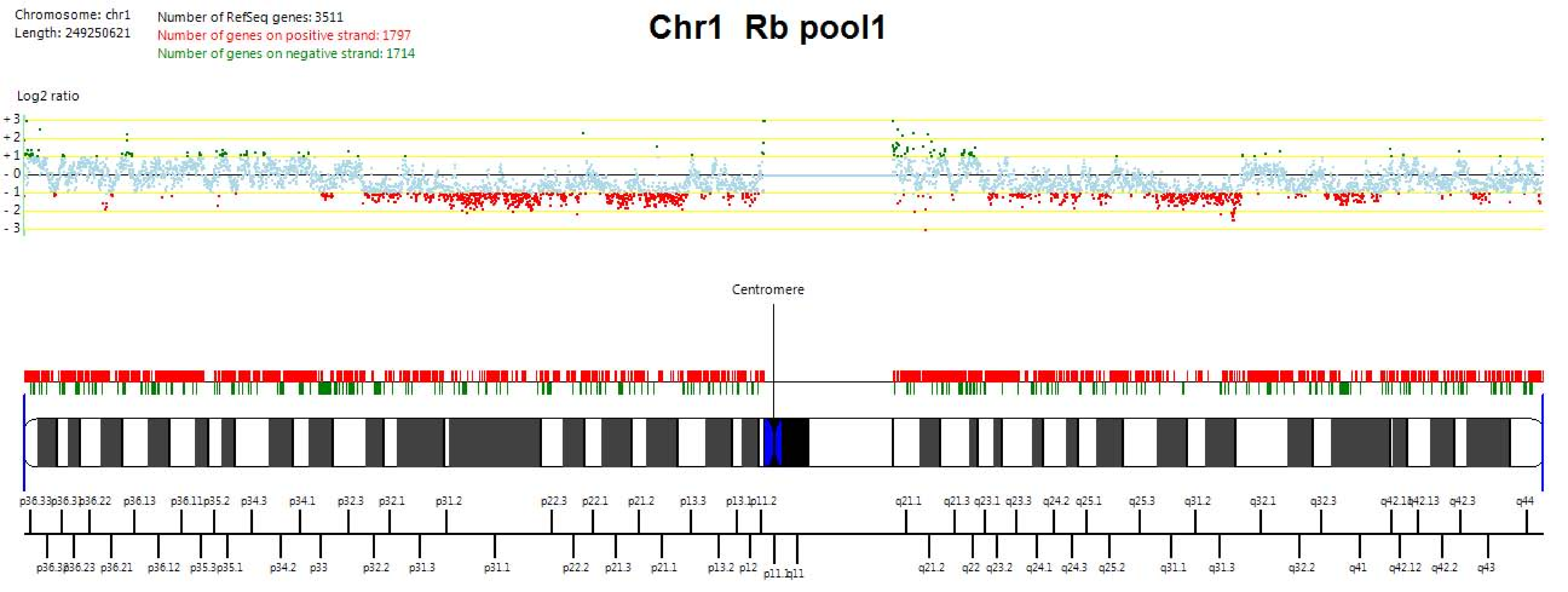
Chromosome: chr1
Length: 249250621
Number of RefSeq genes: 3511
Number of genes on positive strand: 1797
Number of genes on negative strand: 1714
Chr1 Rb pool1
Log2 ratio
+3
+2
+1
0
-1
-2
-3
Centromere

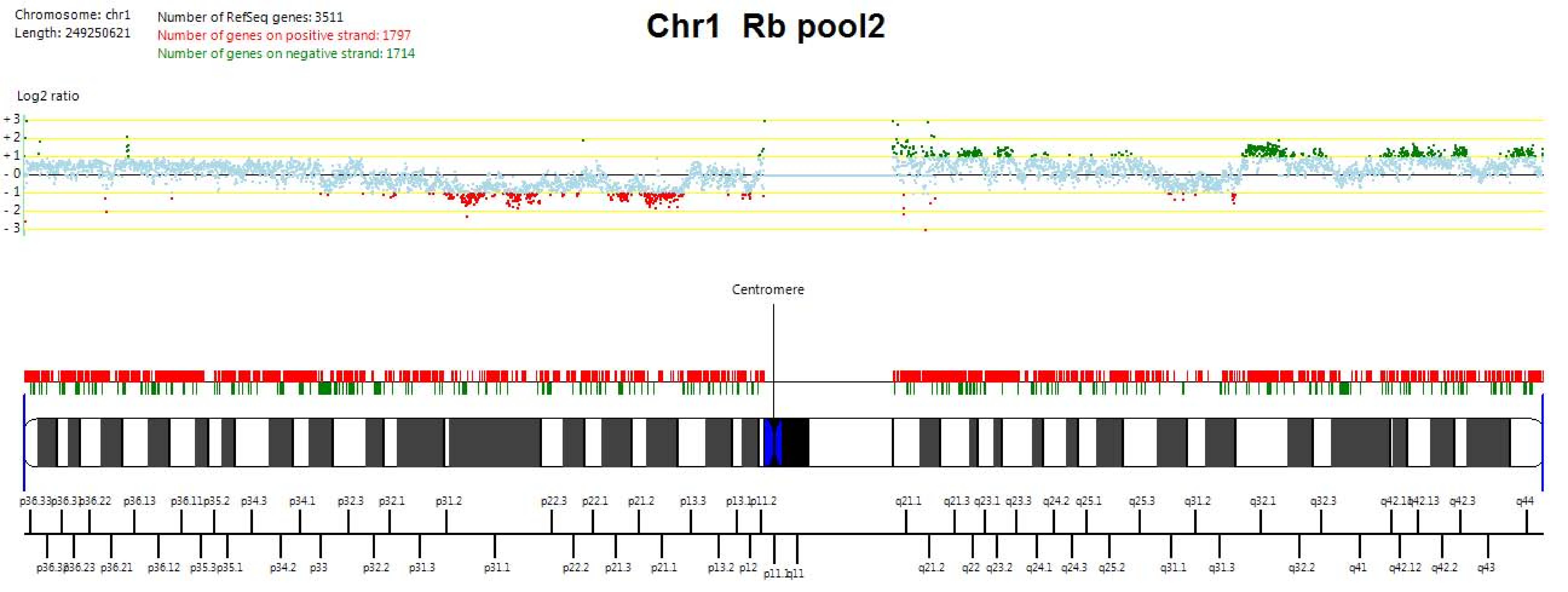
Chromosome: chr1
Length: 249250621
Number of RefSeq genes: 3511
Number of genes on positive strand: 1797
Number of genes on negative strand: 1714
Chr1 Rb pool2
Log2 ratio
+3
+2
+1
-0
-1
-2
-3
Centromere
p36.33
p36.22
p36.13
p36.11
p35.2
p34.3
p34.1
p32.3
p32.1
p31.2
p22.3
p22.1
p21.2
p13.3
p11.2
q21.1
q21.3
q23.1
q23.3
q24.2
q25.1
q25.3
q31.2
q32.1
q32.3
q42.13
q42.3
q44
p36.23
p36.21
p36.12
p35.1
p34.2
p33
p32.2
p31.3
p31.1
p22.2
p21.3
p21.1
p13.2
p12
q21.2
q22
q23.2
q24.1
q24.3
q25.2
q31.1
q31.3
q32.2
q41
q42.12
q42.2
q43

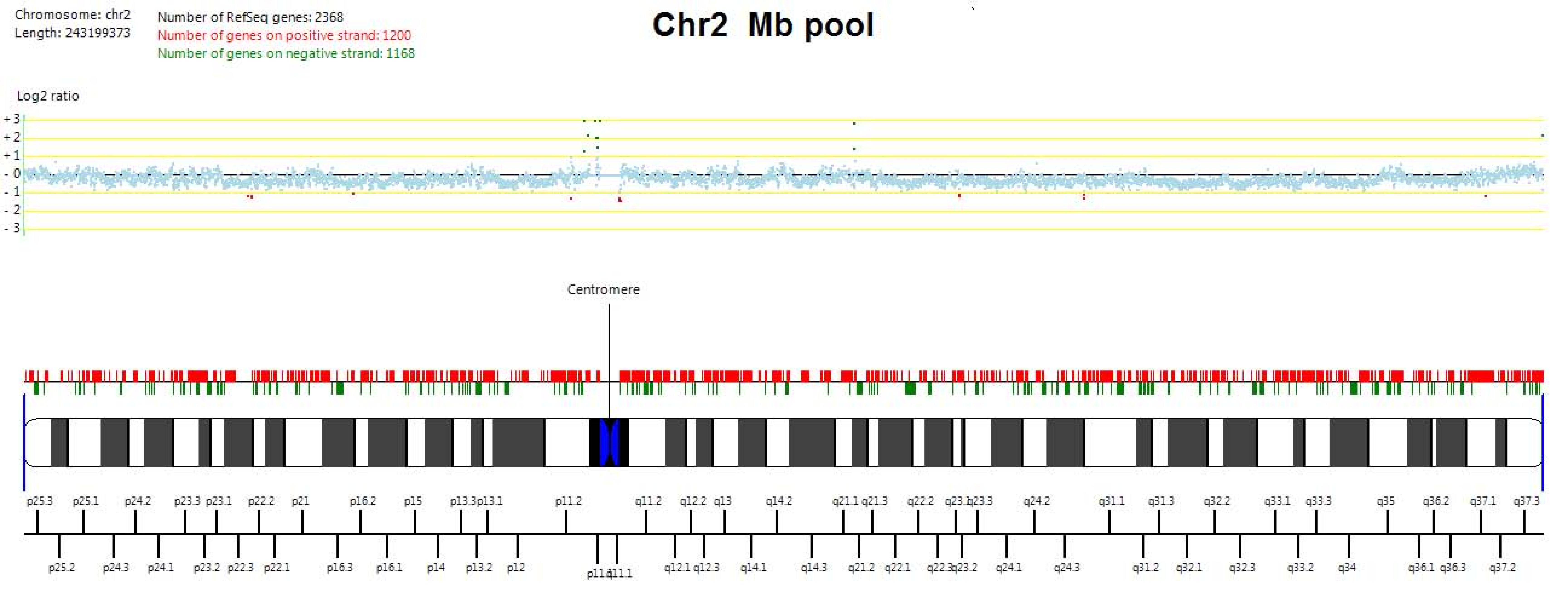
Chromosome: chr2
Length: 243199373
Number of RefSeq genes: 2368
Number of genes on positive strand: 1200
Number of genes on negative strand: 1168
Chr2 Mb pool
Log2 ratio
+3
+2
+1
-0
-1
-2
-3
Centromere
p25.3 p25.1 p24.2 p23.3 p23.1 p22.2 p21 p16.2 p15 p13.3 p13.1 p11.2 q11.2 q12.2 q13 q14.2 q21.1 q21.3 q22.2 q23.1 q23.3 q24.2 q31.1 q31.3 q32.2 q33.1 q33.3 q35 q36.2 q37.1 q37.3
p25.2 p24.3 p24.1 p23.2 p22.3 p22.1 p16.3 p16.1 p14 p13.2 p12 q12.1 q12.3 q14.1 q14.3 q21.2 q22.1 q23.2 q24.1 q24.3 q31.2 q32.1 q32.3 q33.2 q34 q36.1 q36.3 q37.2

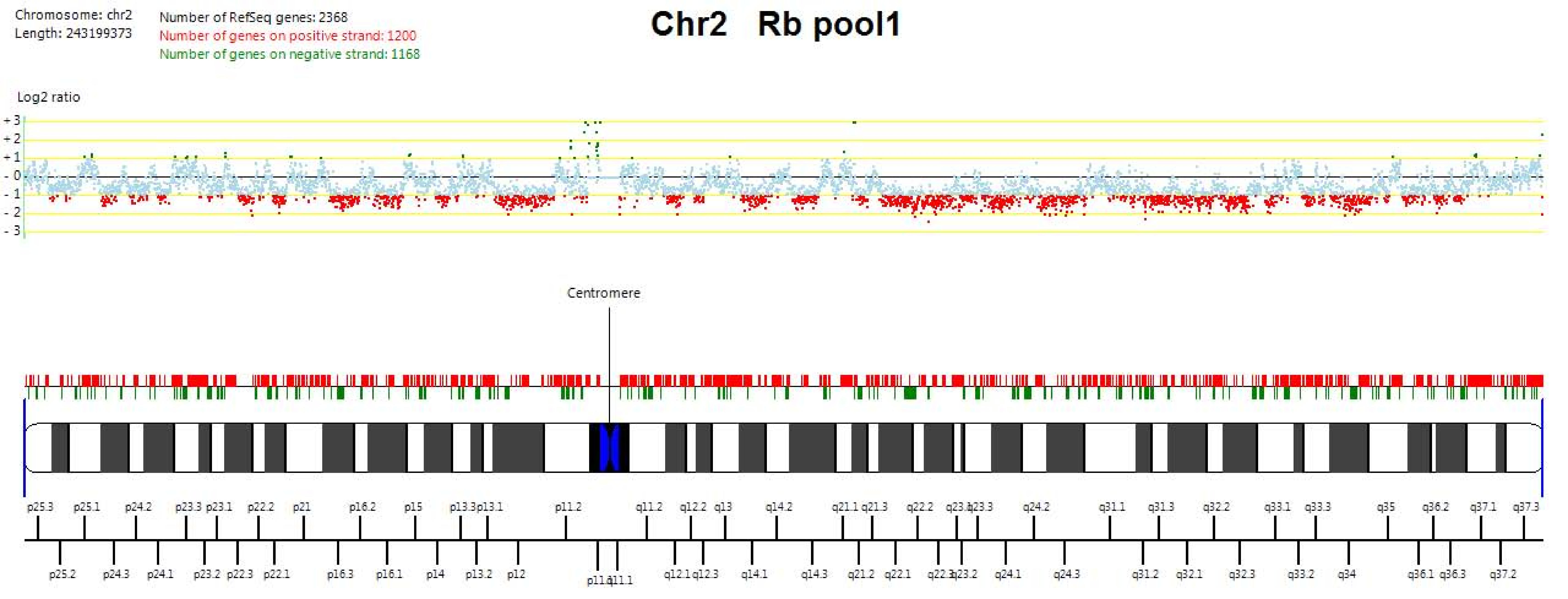

Chromosome: chr2
Length: 243199373
Number of RefSeq genes: 2368
Number of genes on positive strand: 1200
Number of genes on negative strand: 1168
Chr2 Rb pool1
Log2 ratio
+3
+2
+1
-0
-1
-2
-3
Centromere
p25.3 p25.1 p24.2 p23.3 p23.1 p22.2 p21 p16.2 p15 p13.3 p13.1 p11.2 q11.2 q12.2 q13 q14.2 q21.1 q21.3 q22.2 q23.1 q23.3 q24.2 q31.1 q31.3 q32.2 q33.1 q33.3 q35 q36.2 q37.1 q37.3
p25.2 p24.3 p24.1 p23.2 p22.3 p22.1 p16.3 p16.1 p14 p13.2 p12 p11.1 q11.1 q12.1 q12.3 q14.1 q14.3 q21.2 q22.1 q22.3 q23.2 q24.1 q24.3 q31.2 q32.1 q32.3 q33.2 q34 q36.1 q36.3 q37.2

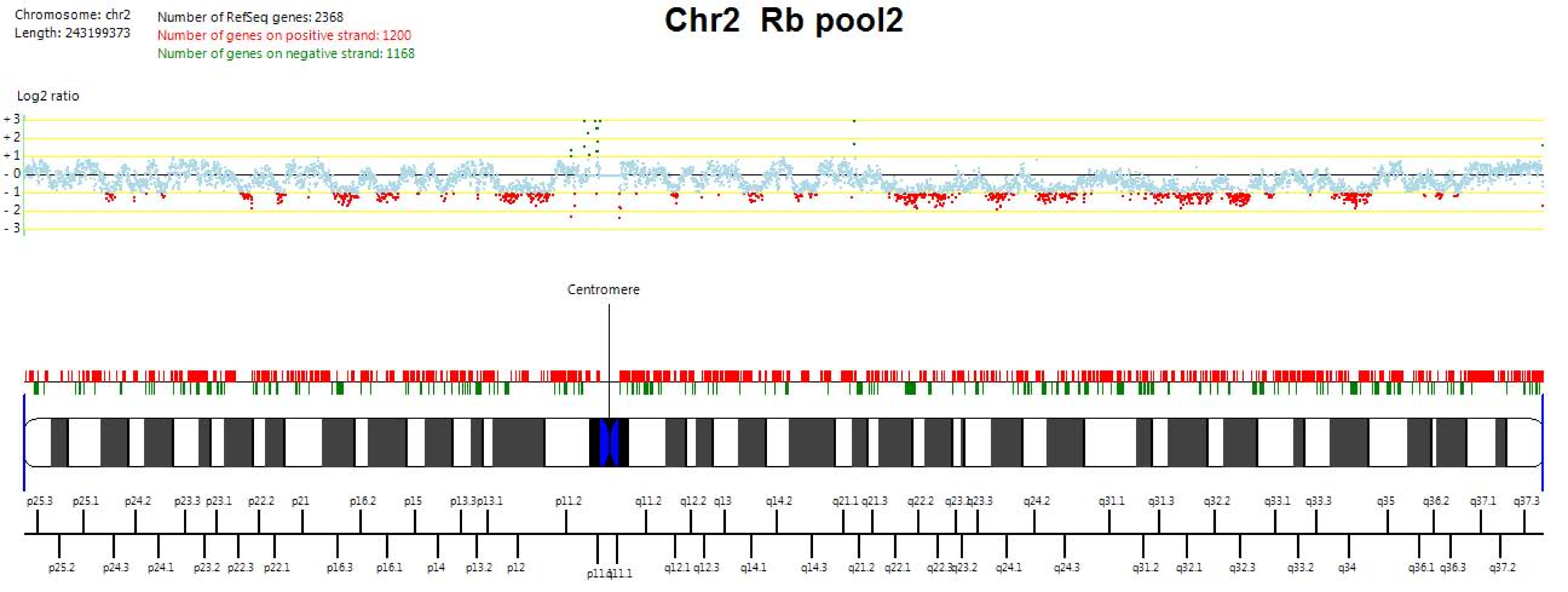
Chromosome: chr2
Length: 243199373
Number of RefSeq genes: 2368
Number of genes on positive strand: 1200
Number of genes on negative strand: 1168
Chr2 Rb pool2
Log2 ratio
+3
+2
+1
-0
-1
-2
-3
Centromere
p25.3
p25.1
p24.2
p23.3
p23.1
p22.2
p21
p16.2
p15
p13.3
p13.1
p11.2
q11.2
q12.2
q13
q14.2
q21.1
q21.3
q22.2
q23.3
q24.2
q31.1
q31.3
q32.2
q33.1
q33.3
q35
q36.2
q37.1
q37.3
p25.2
p24.3
p24.1
p23.2
p22.3
p22.1
p16.3
p16.1
p14
p13.2
p12
q11.1
q12.1
q12.3
q14.1
q14.3
q21.2
q22.1
q23.2
q24.1
q24.3
q31.2
q32.1
q32.3
q33.2
q34
q36.1
q36.3
q37.2

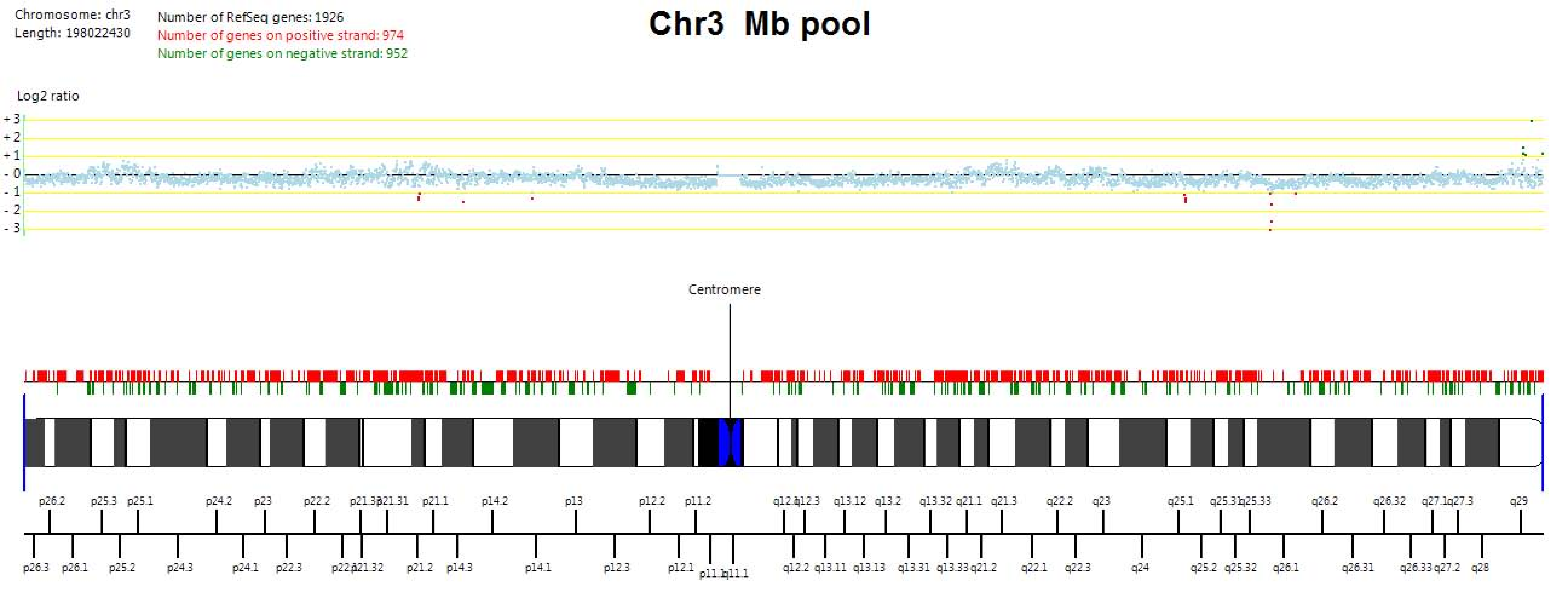

Chromosome: chr3
Length: 198022430
Number of RefSeq genes: 1926
Number of genes on positive strand: 974
Number of genes on negative strand: 952
Chr3 Mb pool
Log2 ratio
+3
+2
+1
-0
-1
-2
-3
Centromere
p26.2
p25.3
p25.1
p24.2
p23
p22.2
p21.31
p21.1
p14.2
p13
p12.2
p11.2
q12.3
q13.12
q13.2
q13.32
q21.1
q21.3
q22.2
q23
q25.1
q25.33
q26.2
q26.32
q27.3
q29
p26.3
p26.1
p25.2
p24.3
p24.1
p22.3
p21.32
p21.2
p14.3
p14.1
p12.3
p12.1
q11.1
q12.2
q13.11
q13.13
q13.31
q13.33
q21.2
q22.1
q22.3
q24
q25.2
q25.32
q26.1
q26.31
q26.33
q27.2
q28

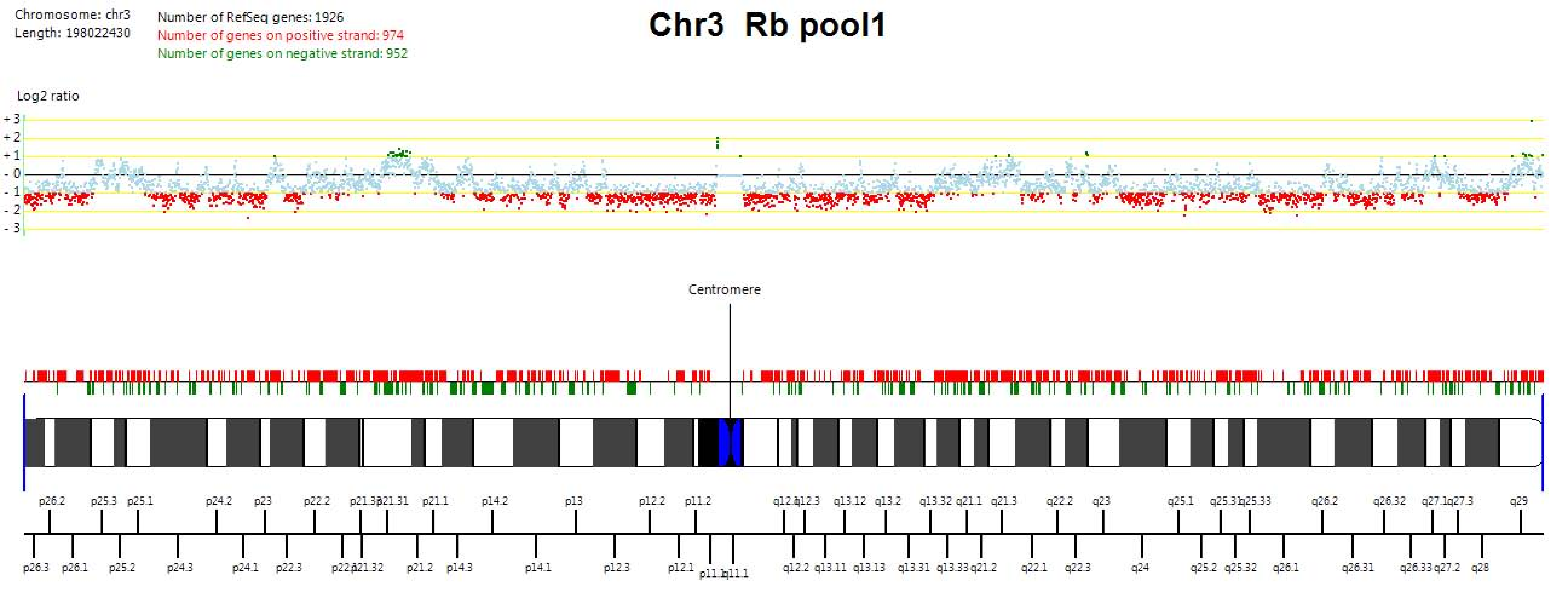
Chromosome: chr3
Length: 198022430
Number of RefSeq genes: 1926
Number of genes on positive strand: 974
Number of genes on negative strand: 952
Chr3 Rb pool1
Log2 ratio
+3
+2
+1
-0
-1
-2
-3
Centromere
p26.2 p25.3 p25.1 p24.2 p23 p22.2 p21.31 p21.1 p14.2 p13 p12.2 p11.2 q12.3 q13.12 q13.2 q13.32 q21.1 q21.3 q22.2 q23 q25.1 q25.33 q26.2 q26.32 q27.3 q29
p26.3 p26.1 p25.2 p24.3 p24.1 p22.3 p21.32 p21.2 p14.3 p14.1 p12.3 p12.1 q12.2 q13.11 q13.13 q13.31 q13.33 q21.2 q22.1 q22.3 q24 q25.2 q25.32 q26.1 q26.31 q27.2 q28

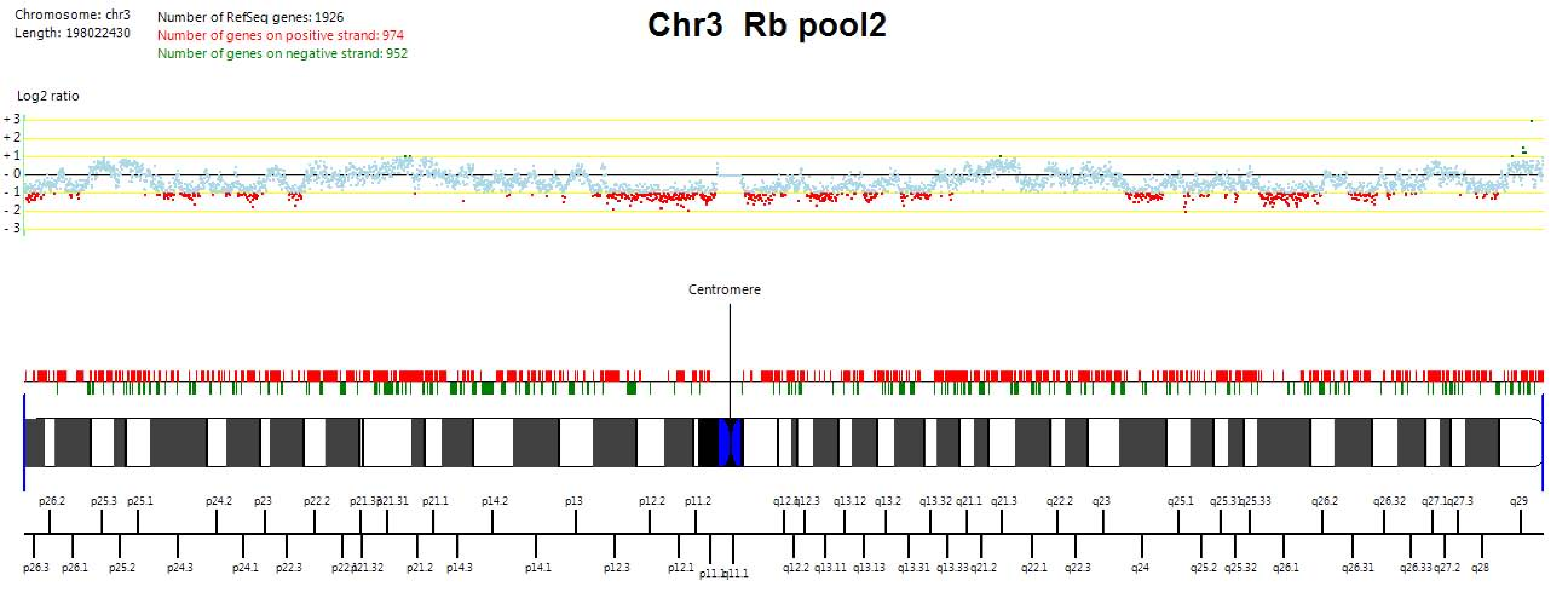

Chromosome: chr3
Length: 198022430
Number of RefSeq genes: 1926
Number of genes on positive strand: 974
Number of genes on negative strand: 952
Chr3 Rb pool2
Log2 ratio
+3
+2
+1
-0
-1
-2
-3
Centromere
p26.2 p25.3 p25.1 p24.2 p23 p22.2 p21.33 p21.31 p21.1 p14.2 p13 p12.2 p11.2 q12.1 q12.3 q13.12 q13.2 q13.32 q21.1 q21.3 q22.2 q23 q25.1 q25.31 q25.33 q26.2 q26.32 q27.1 q27.3 q29
p26.3 p26.1 p25.2 p24.3 p24.1 p22.3 p22.1 p21.32 p21.2 p14.3 p14.1 p12.3 p12.1 p11.1 q11.1 q12.2 q13.11 q13.13 q13.31 q13.33 q21.2 q22.1 q22.3 q24 q25.2 q25.32 q26.1 q26.31 q26.33 q27.2 q28

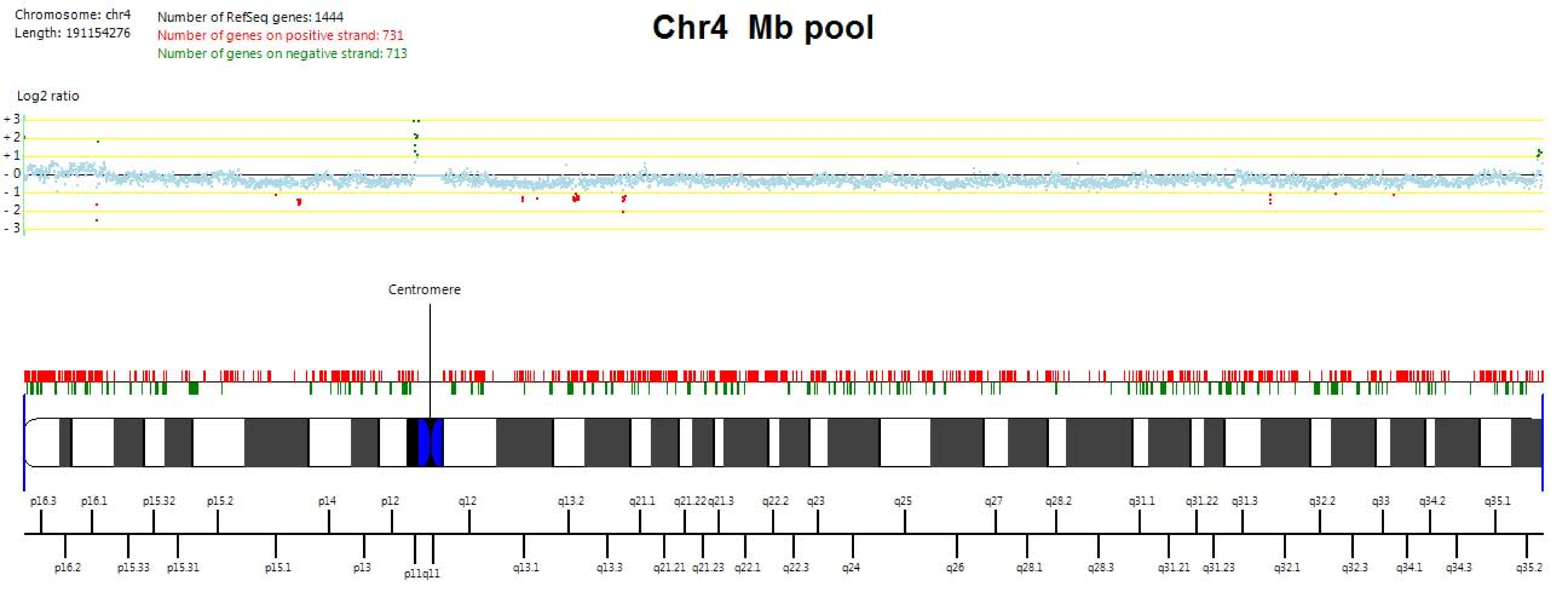

Chromosome: chr4
Length: 191154276
Number of RefSeq genes: 1444
Number of genes on positive strand: 731
Number of genes on negative strand: 713
Chr4 Mb pool
Log2 ratio
+3
+2
+1
- 0
- 1
- 2
- 3
Centromere
p16.3 p16.1 p15.32 p15.2 p14 p12 q12 q13.2 q21.1 q21.22 q21.3 q22.2 q23 q25 q27 q28.2 q31.1 q31.22 q31.3 q32.2 q33 q34.2 q35.1
p16.2 p15.33 p15.31 p15.1 p13 p11 q11 q13.1 q13.3 q21.21 q21.23 q22.1 q22.3 q24 q26 q28.1 q28.3 q31.21 q31.23 q32.1 q32.3 q34.1 q34.3 q35.2

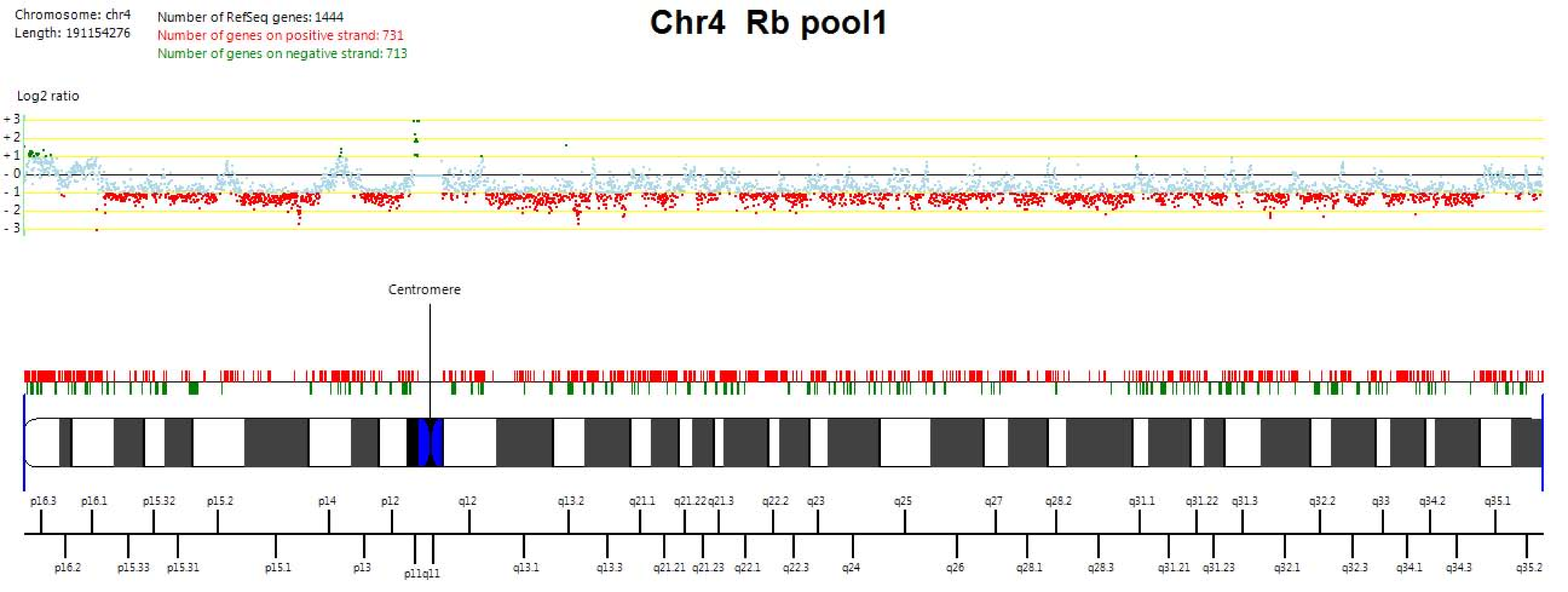
Chromosome: chr4
Length: 191154276
Number of RefSeq genes: 1444
Number of genes on positive strand: 731
Number of genes on negative strand: 713
Chr4 Rb pool1
Log2 ratio
+3
+2
+1
-0
-1
-2
-3
Centromere
p16.3
p16.1
p15.32
p15.2
p14
p12
q12
q13.2
q21.1
q21.22
q21.3
q22.2
q23
q25
q27
q28.2
q31.1
q31.22
q31.3
q32.2
q33
q34.2
q35.1
p16.2
p15.33
p15.31
p15.1
p13
p11
q11
q13.1
q13.3
q21.21
q21.23
q22.1
q22.3
q24
q26
q28.1
q28.3
q31.21
q31.23
q32.1
q32.3
q34.1
q34.3
q35.2

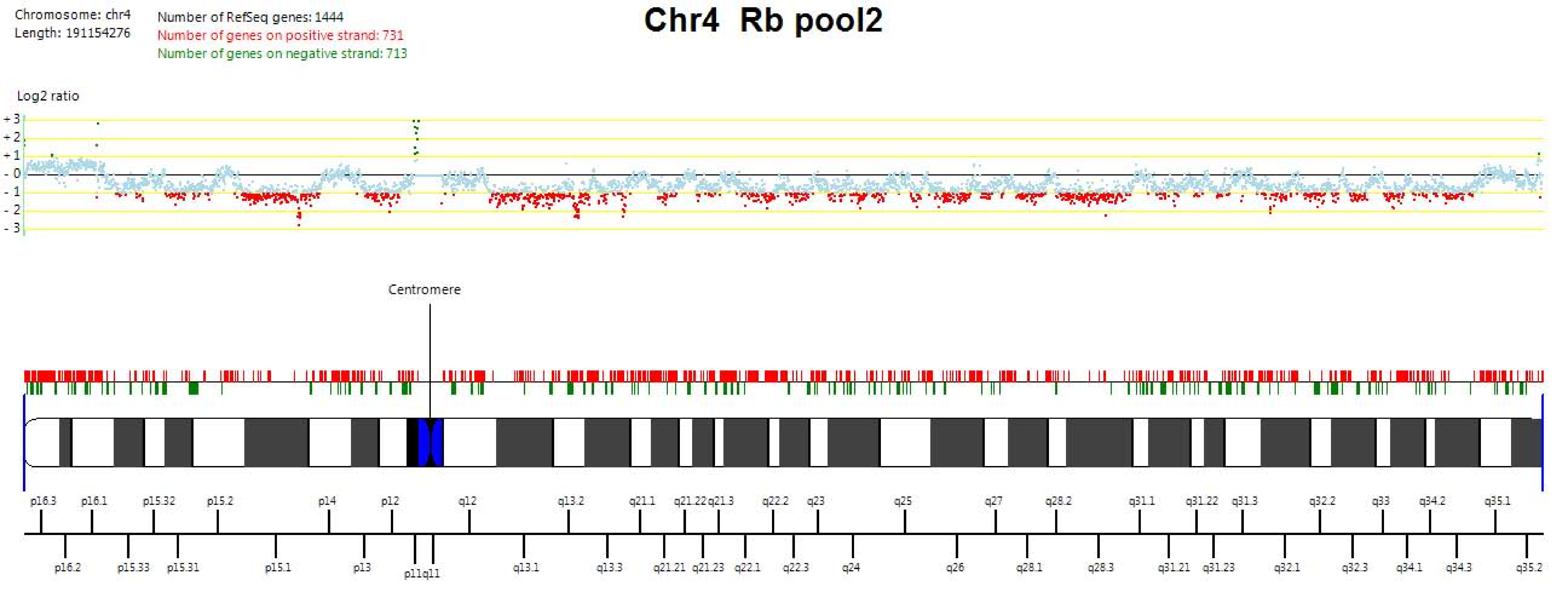
Chromosome: chr4
Length: 191154276
Number of RefSeq genes: 1444
Number of genes on positive strand: 731
Number of genes on negative strand: 713
Chr4 Rb pool2
Log2 ratio
+3
+2
+1
-0
-1
-2
-3
Centromere
p16.3 p16.1 p15.32 p15.2 p14 p12 q12 q13.2 q21.1 q21.22 q21.3 q22.2 q23 q25 q27 q28.2 q31.1 q31.22 q31.3 q32.2 q33 q34.2 q35.1
p16.2 p15.33 p15.31 p15.1 p13 p11 q11 q13.1 q13.3 q21.21 q21.23 q22.1 q22.3 q24 q26 q28.1 q28.3 q31.21 q31.23 q32.1 q32.3 q34.1 q34.3 q35.2

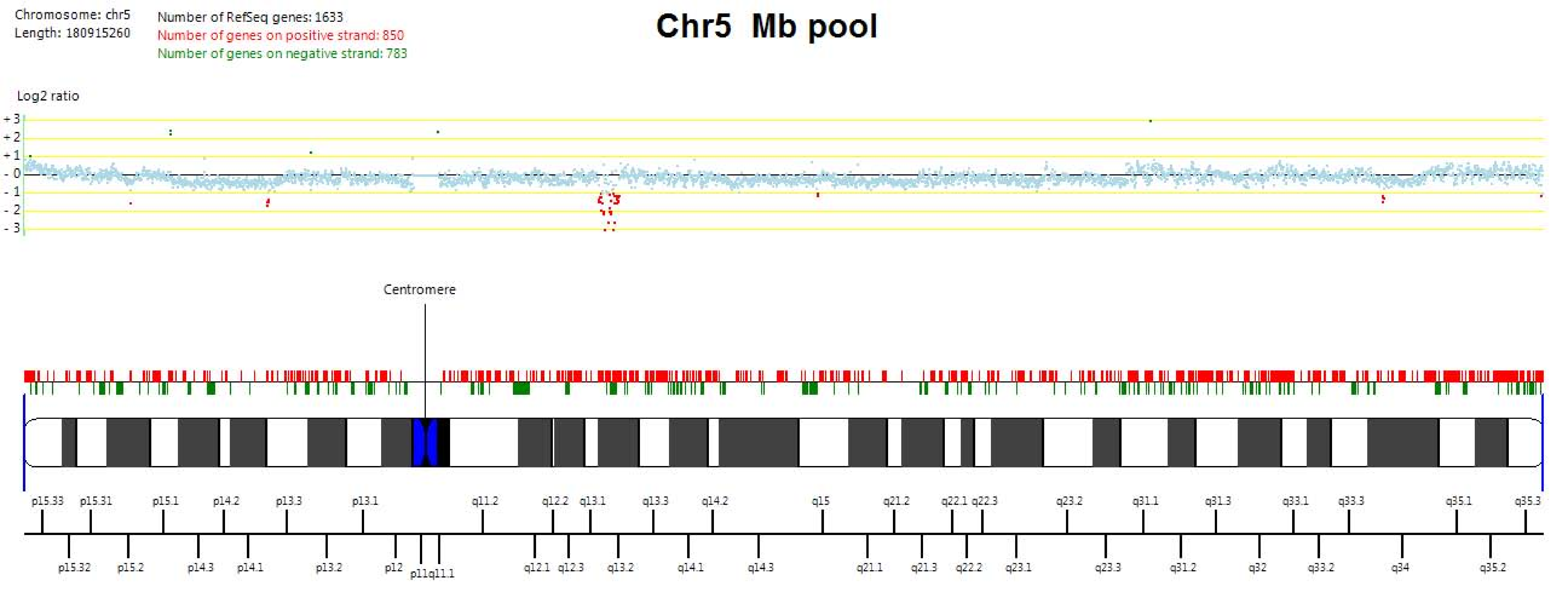

Chromosome: chr5
Length: 180915260
Number of RefSeq genes: 1633
Number of genes on positive strand: 850
Number of genes on negative strand: 783
Chr5 Mb pool
Log2 ratio
+3
+2
+1
- 0
- 1
- 2
- 3
Centromere
p15.33
p15.31
p15.1
p14.2
p13.3
p13.1
q11.2
q12.2
q13.1
q13.3
q14.2
q15
q21.2
q22.1
q22.3
q23.2
q31.1
q31.3
q33.1
q33.3
q35.1
q35.3
p15.32
p15.2
p14.3
p14.1
p13.2
p12
p11
q11.1
q12.1
q12.3
q13.2
q14.1
q14.3
q21.1
q21.3
q22.2
q23.1
q23.3
q31.2
q32
q33.2
q34
q35.2

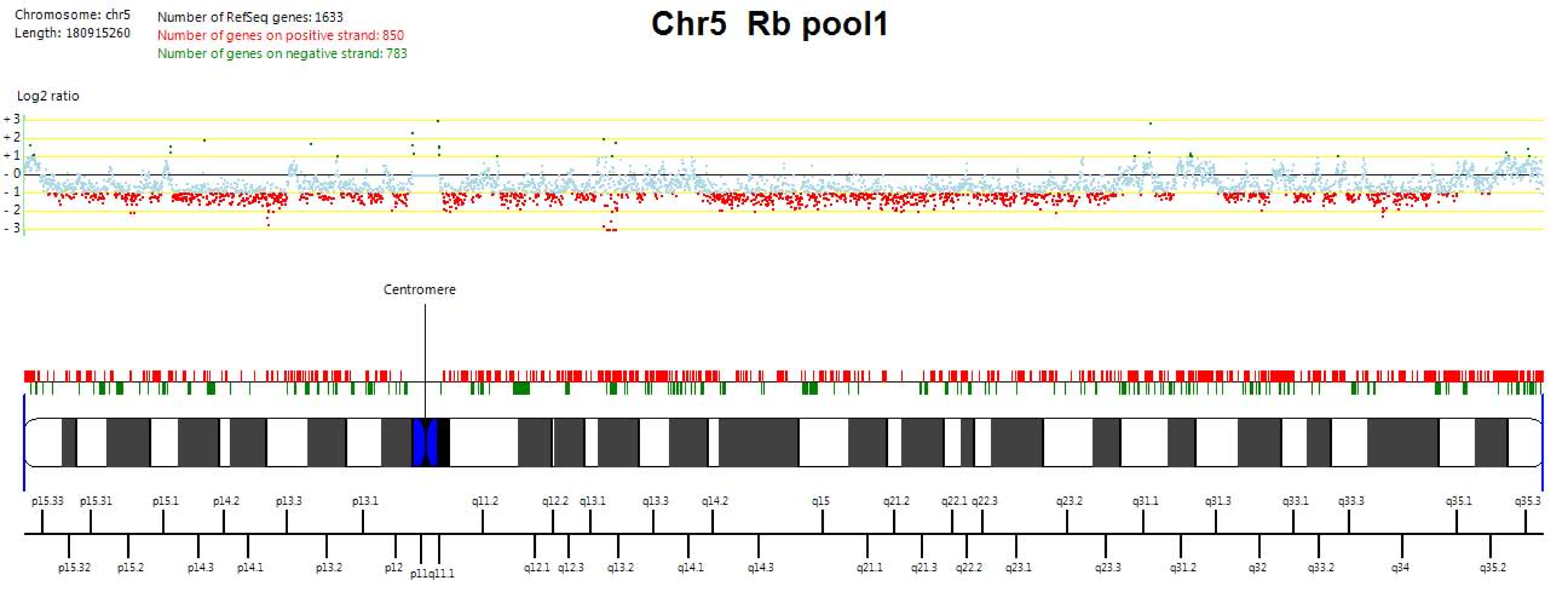
Chromosome: chr5
Length: 180915260
Number of RefSeq genes: 1633
Number of genes on positive strand: 850
Number of genes on negative strand: 783
Chr5 Rb pool1
Log2 ratio
+3
+2
+1
-0
-1
-2
-3
Centromere
p15.33
p15.31
p15.1
p14.2
p13.3
p13.1
q11.2
q12.2
q13.1
q13.3
q14.2
q15
q21.2
q22.1
q22.3
q23.2
q31.1
q31.3
q33.1
q33.3
q35.1
q35.3
p15.32
p15.2
p14.3
p14.1
p13.2
p12
p11
q11.1
q12.1
q12.3
q13.2
q14.1
q14.3
q21.1
q21.3
q22.2
q23.1
q23.3
q31.2
q32
q33.2
q34
q35.2

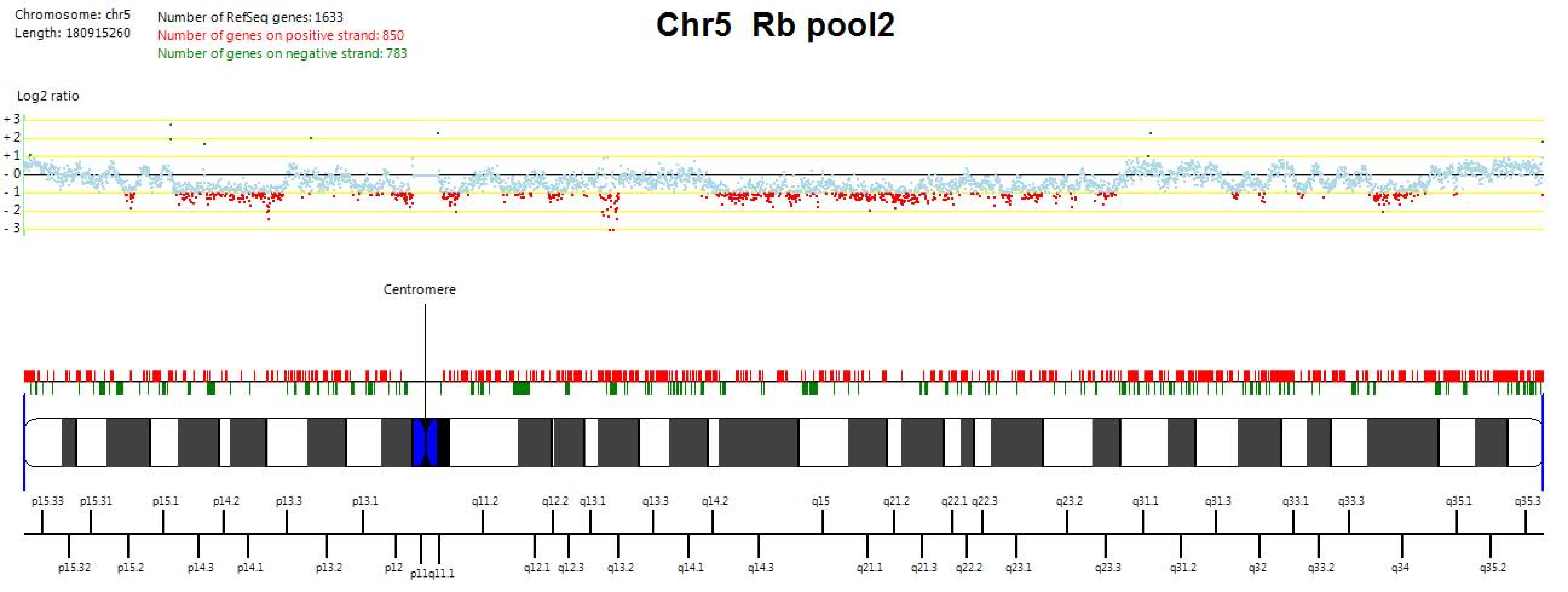
Chromosome: chr5
Length: 180915260
Number of RefSeq genes: 1633
Number of genes on positive strand: 850
Number of genes on negative strand: 783
Chr5 Rb pool2
Log2 ratio
+3
+2
+1
-0
-1
-2
-3
Centromere
p15.33
p15.31
p15.1
p14.2
p13.3
p13.1
q11.2
q12.2
q13.1
q13.3
q14.2
q15
q21.2
q22.1
q22.3
q23.2
q31.1
q31.3
q33.1
q33.3
q35.1
q35.3
p15.32
p15.2
p14.3
p14.1
p13.2
p12
p11
q11.1
q12.1
q12.3
q13.2
q14.1
q14.3
q21.1
q21.3
q22.2
q23.1
q23.3
q31.2
q32
q33.2
q34
q35.2

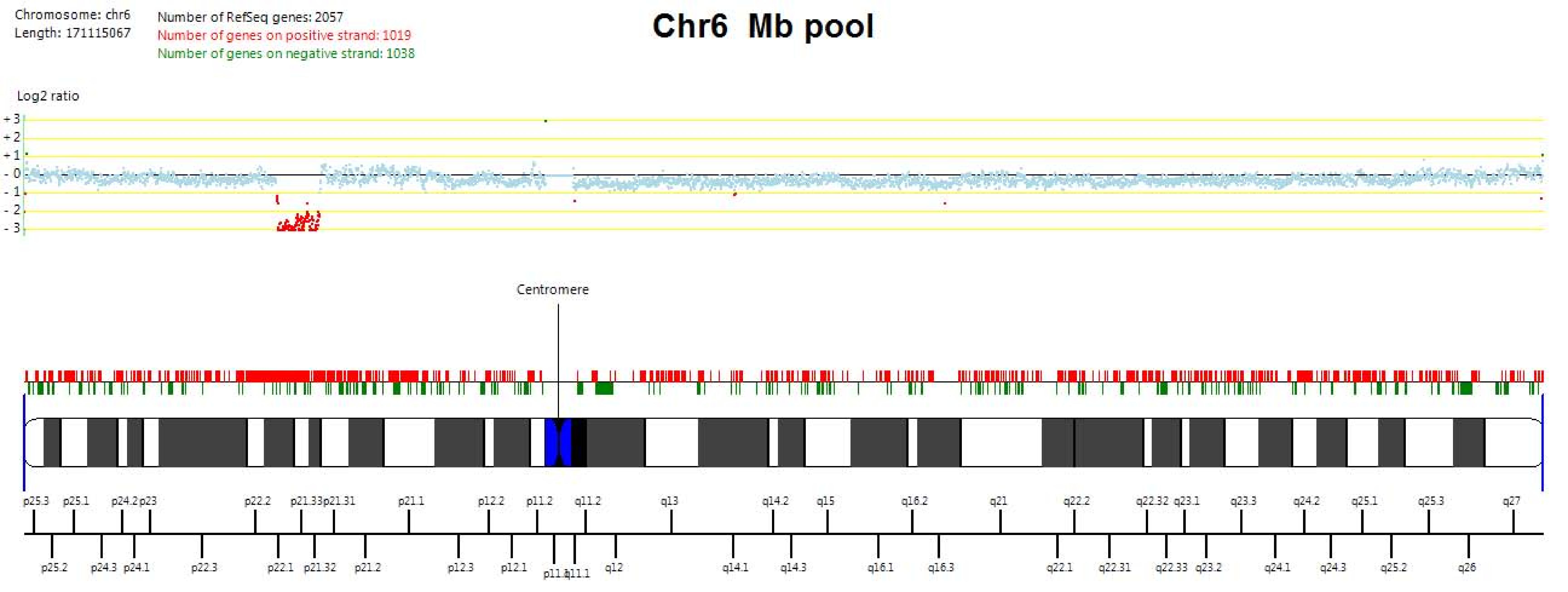
Chromosome: chr6
Length: 171115067
Number of RefSeq genes: 2057
Number of genes on positive strand: 1019
Number of genes on negative strand: 1038
Chr6 Mb pool
Log2 ratio
+3
+2
+1
- 0
- 1
- 2
- 3
Centromere
p25.3 p25.1 p24.2 p23 p22.2 p21.33 p21.31 p21.1 p12.2 p11.2 q11.2 q13 q14.2 q15 q16.2 q21 q22.2 q22.32 q23.1 q23.3 q24.2 q25.1 q25.3 q27
p25.2 p24.3 p24.1 p22.3 p22.1 p21.32 p21.2 p12.3 p12.1 p11.1 q11.1 q12 q14.1 q14.3 q16.1 q16.3 q22.1 q22.31 q22.33 q23.2 q24.1 q24.3 q25.2 q26

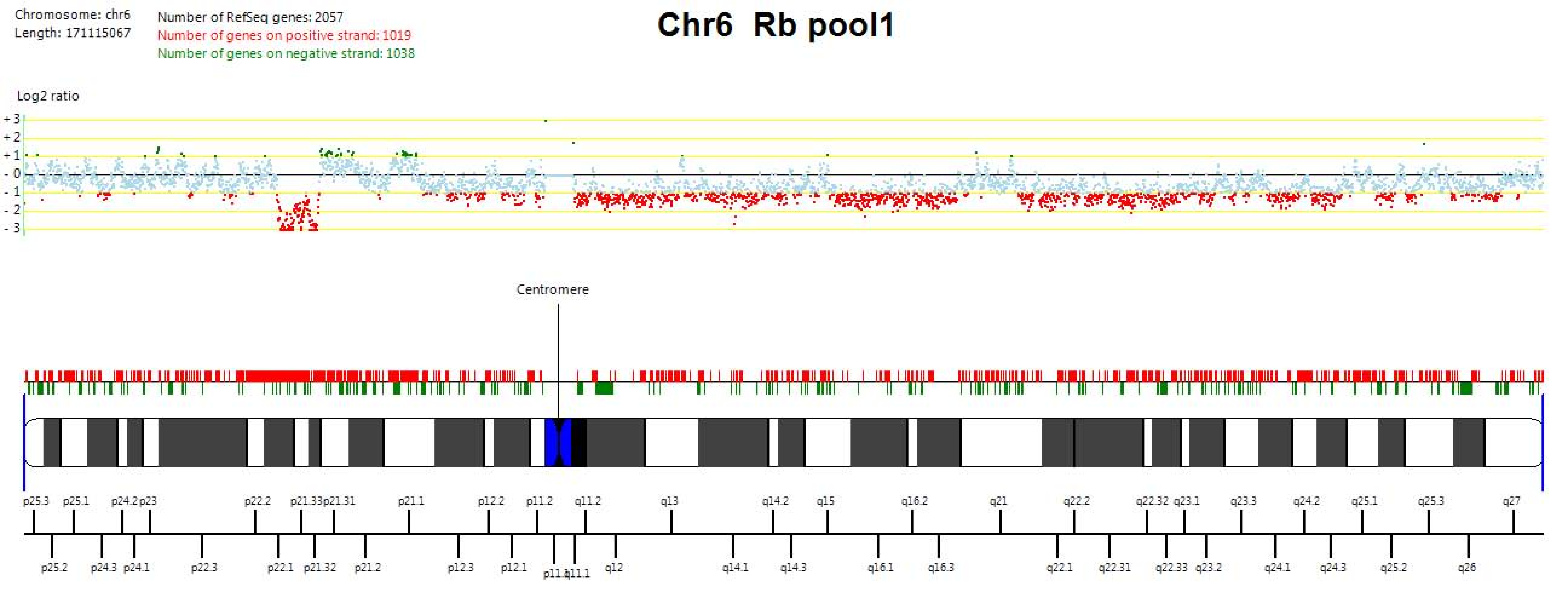

Chromosome: chr6
Length: 171115067
Number of RefSeq genes: 2057
Number of genes on positive strand: 1019
Number of genes on negative strand: 1038
Chr6 Rb pool1
Log2 ratio
+3
+2
+1
-0
-1
-2
-3
Centromere
p25.3 p25.1 p24.2 p23 p22.2 p21.33 p21.31 p21.1 p12.2 p11.2 q11.2 q13 q14.2 q15 q16.2 q21 q22.2 q22.32 q23.1 q23.3 q24.2 q25.1 q25.3 q27
p25.2 p24.3 p24.1 p22.3 p22.1 p21.32 p21.2 p12.3 p12.1 p11.1 q11.1 q12 q14.1 q14.3 q16.1 q16.3 q22.1 q22.31 q22.33 q23.2 q24.1 q24.3 q25.2 q26

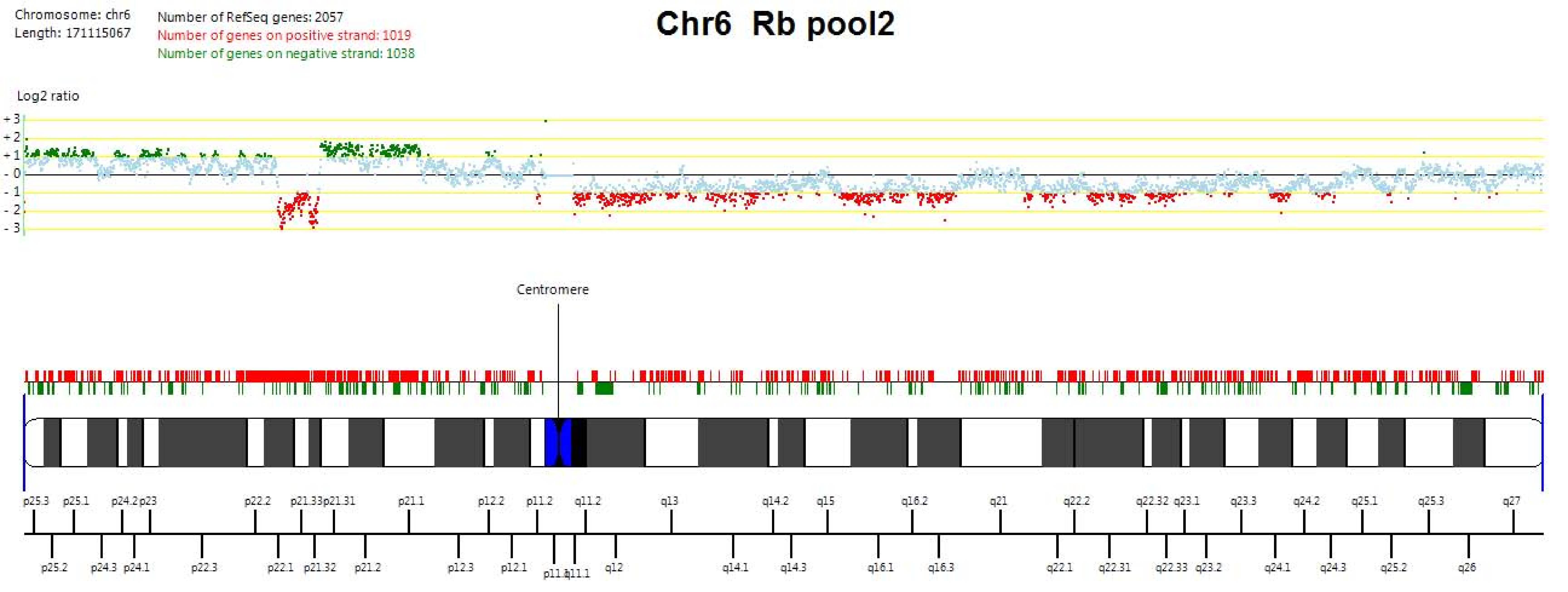

Chromosome: chr6
Length: 171115067
Number of RefSeq genes: 2057
Number of genes on positive strand: 1019
Number of genes on negative strand: 1038
Chr6 Rb pool2
Log2 ratio
+3
+2
+1
-0
-1
-2
-3
Centromere
p25.3
p25.1
p24.2
p23
p22.2
p21.33
p21.31
p21.1
p12.2
p11.2
q11.2
q13
q14.2
q15
q16.2
q21
q22.2
q22.32
q23.1
q23.3
q24.2
q25.1
q25.3
q27
p25.2
p24.3
p24.1
p22.3
p22.1
p21.32
p21.2
p12.3
p12.1
q12
q14.1
q14.3
q16.1
q16.3
q22.1
q22.31
q22.33
q23.2
q24.1
q24.3
q25.2
q26

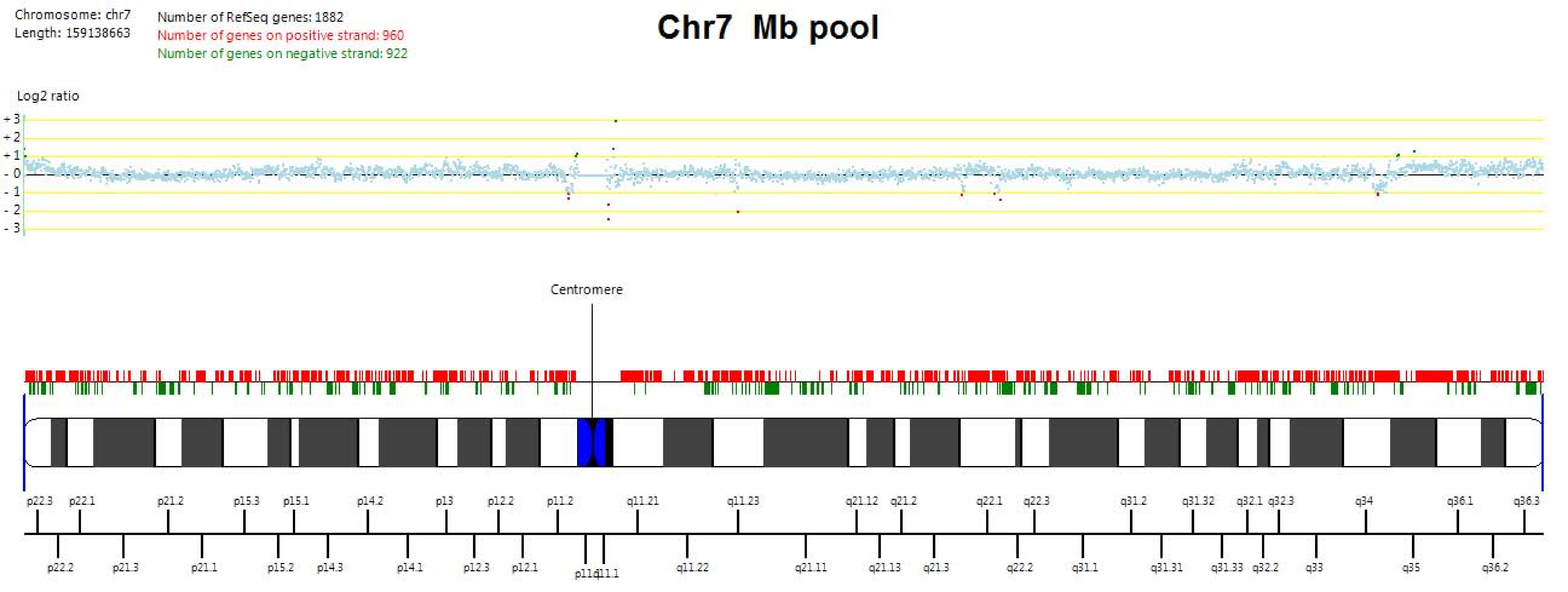
Chromosome: chr7
Length: 159138663
Number of RefSeq genes: 1882
Number of genes on positive strand: 960
Number of genes on negative strand: 922
Chr7 Mb pool
Log2 ratio
+3
+2
+1
-0
-1
-2
-3
Centromere
p22.3
p22.1
p21.2
p15.3
p15.1
p14.2
p13
p12.2
p11.2
q11.21
q11.23
q21.12
q21.2
q22.1
q22.3
q31.2
q31.32
q32.1
q32.3
q34
q36.1
q36.3
p22.2
p21.3
p21.1
p15.2
p14.3
p14.1
p12.3
p12.1
p11.1
q11.1
q11.22
q21.11
q21.13
q21.3
q22.2
q31.1
q31.31
q31.33
q32.2
q33
q35
q36.2

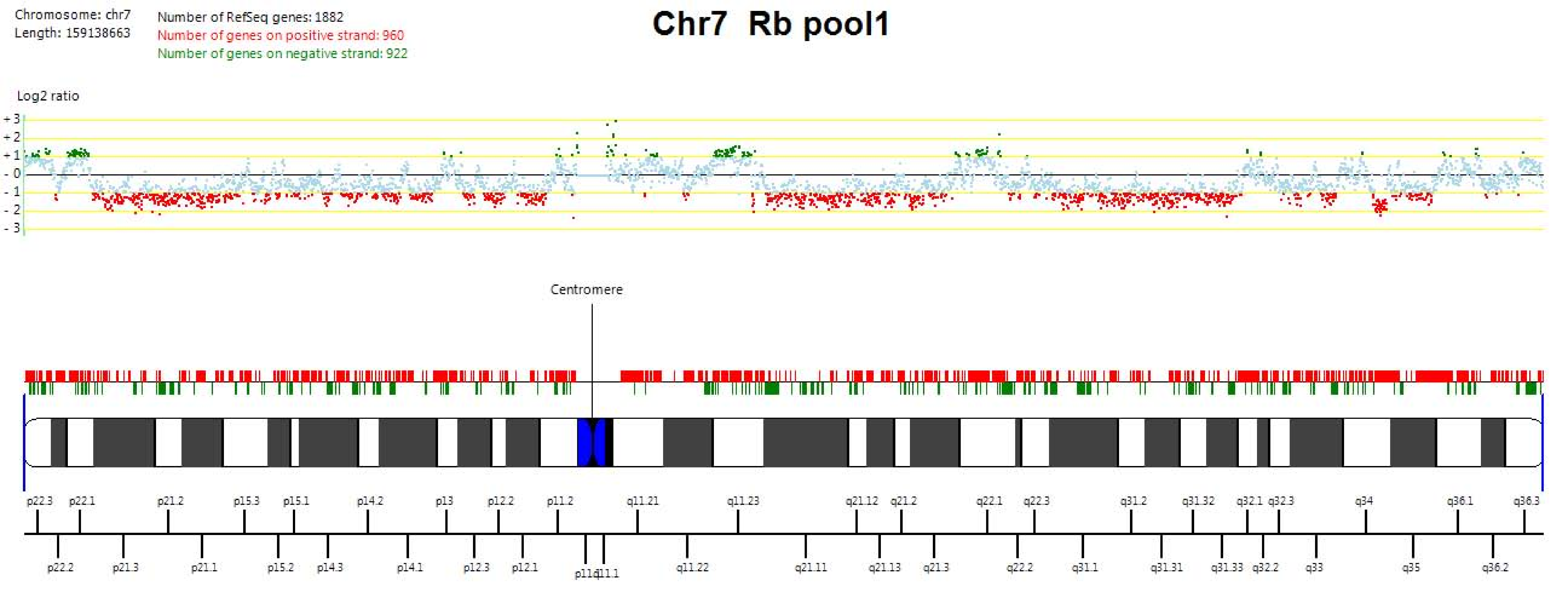

Chromosome: chr7
Length: 159138663
Number of RefSeq genes: 1882
Number of genes on positive strand: 960
Number of genes on negative strand: 922
Chr7 Rb pool1
Log2 ratio
+3
+2
+1
-0
-1
-2
-3
Centromere
p22.3
p22.1
p21.2
p15.3
p15.1
p14.2
p13
p12.2
p11.2
q11.21
q11.23
q21.12
q21.2
q22.1
q22.3
q31.2
q31.32
q32.1
q32.3
q34
q36.1
q36.3
p22.2
p21.3
p21.1
p15.2
p14.3
p14.1
p12.3
p12.1
p11.1
q11.1
q11.22
q21.11
q21.13
q21.3
q22.2
q31.1
q31.31
q31.33
q32.2
q33
q35
q36.2

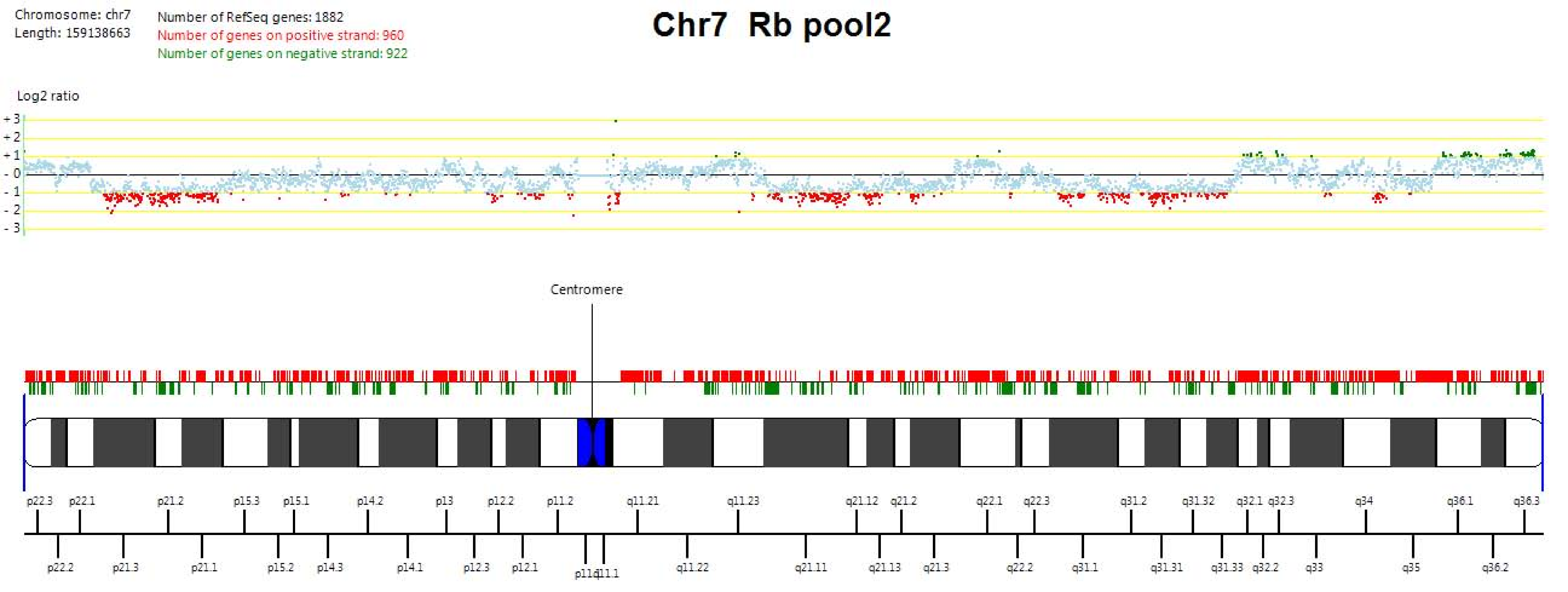
Chromosome: chr7
Length: 159138663
Number of RefSeq genes: 1882
Number of genes on positive strand: 960
Number of genes on negative strand: 922
Chr7 Rb pool2
Log2 ratio
+3
+2
+1
-0
-1
-2
-3
Centromere
p22.3
p22.1
p21.2
p15.3
p15.1
p14.2
p13
p12.2
p11.2
q11.21
q11.23
q21.12
q21.2
q22.1
q22.3
q31.2
q31.32
q32.1
q32.3
q34
q36.1
q36.3
p22.2
p21.3
p21.1
p15.2
p14.3
p14.1
p12.3
p12.1
p11.1
q11.1
q11.22
q21.11
q21.13
q21.3
q22.2
q31.1
q31.31
q31.33
q32.2
q33
q35
q36.2

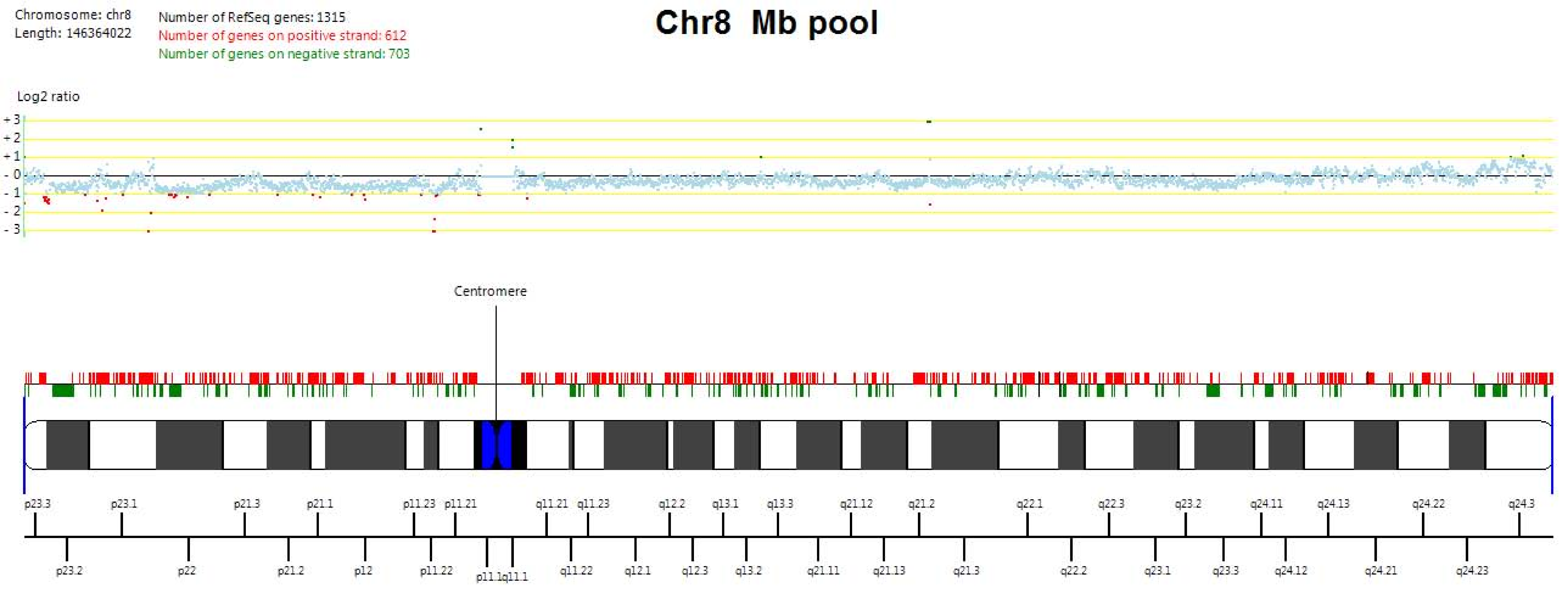
Chromosome: chr8
Length: 146364022
Number of RefSeq genes: 1315
Number of genes on positive strand: 612
Number of genes on negative strand: 703
Chr8 Mb pool
Log2 ratio
+3
+2
+1
-0
-1
-2
-3
Centromere
p23.3
p23.1
p21.3
p21.1
p11.23
p11.21
q11.21
q11.23
q12.2
q13.1
q13.3
q21.12
q21.2
q22.1
q22.3
q23.2
q24.11
q24.13
q24.22
q24.3
p23.2
p22
p21.2
p12
p11.22
p11.1
q11.1
q11.22
q12.1
q12.3
q13.2
q21.11
q21.13
q21.3
q22.2
q23.1
q23.3
q24.12
q24.21
q24.23

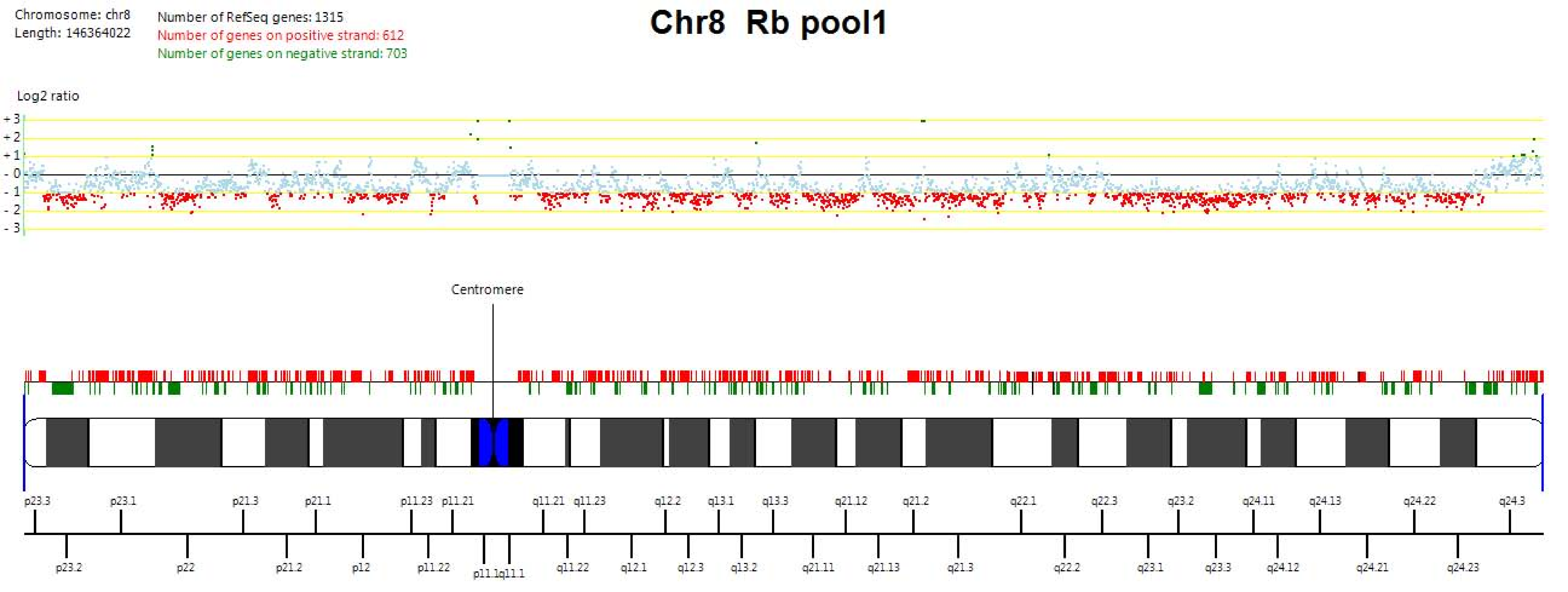
Chr8 Rb pool1
Chromosome: chr8
Length: 146364022
Number of RefSeq genes: 1315
Number of genes on positive strand: 612
Number of genes on negative strand: 703
Log2 ratio
+3
+2
+1
-0
-1
-2
-3
Centromere
p23.3 p23.1 p21.3 p21.1 p11.23 p11.21 q11.21 q11.23 q12.2 q13.1 q13.3 q21.12 q21.2 q22.1 q22.3 q23.2 q24.11 q24.13 q24.22 q24.3
p23.2 p22 p21.2 p12 p11.22 p11.1 q11.1 q11.22 q12.1 q12.3 q13.2 q21.11 q21.13 q21.3 q22.2 q23.1 q23.3 q24.12 q24.21 q24.23

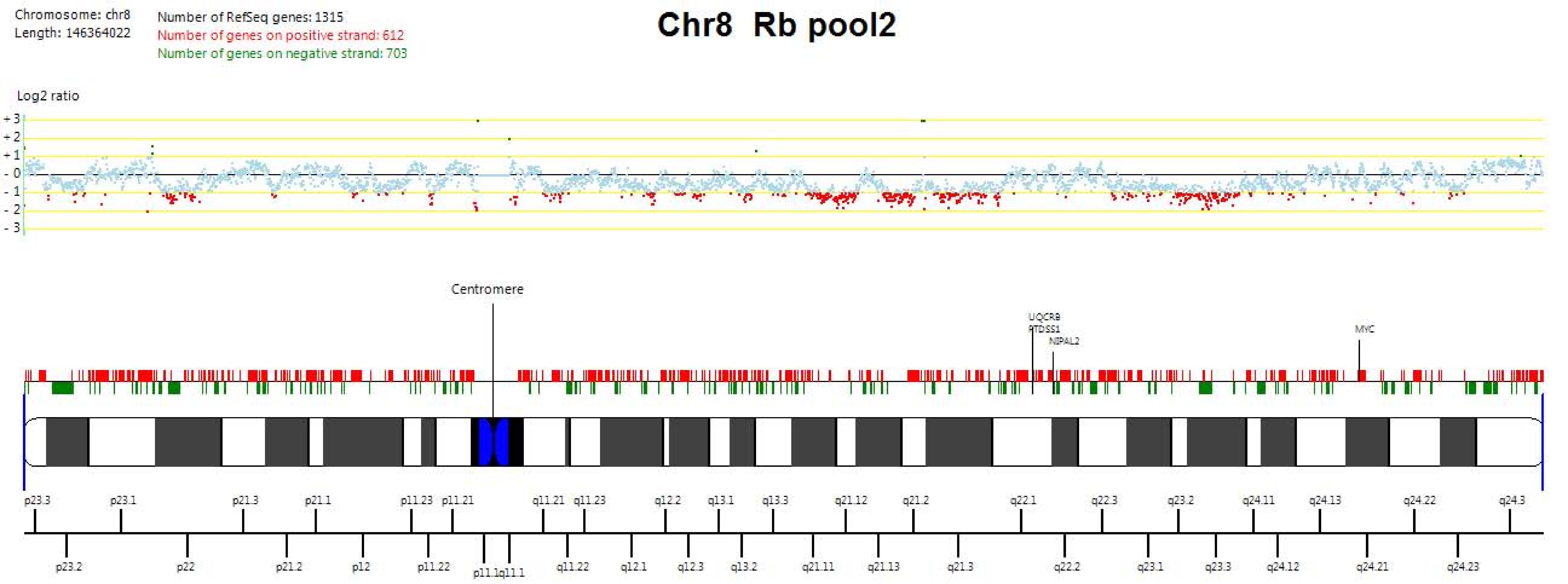

Chromosome: chr8
Length: 146364022
Number of RefSeq genes: 1315
Number of genes on positive strand: 612
Number of genes on negative strand: 703
Chr8 Rb pool2
Log2 ratio
+3
+2
+1
-0
-1
-2
-3
Centromere
UQCRB
PTDSS1
NIPAL2
MYC
p23.3
p23.1
p21.3
p21.1
p11.23
p11.21
q11.21
q11.23
q12.2
q13.1
q13.3
q21.12
q21.2
q22.1
q22.3
q23.2
q24.11
q24.13
q24.22
q24.3
p23.2
p22
p21.2
p12
p11.22
p11.1
q11.1
q11.22
q12.1
q12.3
q13.2
q21.11
q21.13
q21.3
q22.2
q23.1
q23.3
q24.12
q24.21
q24.23

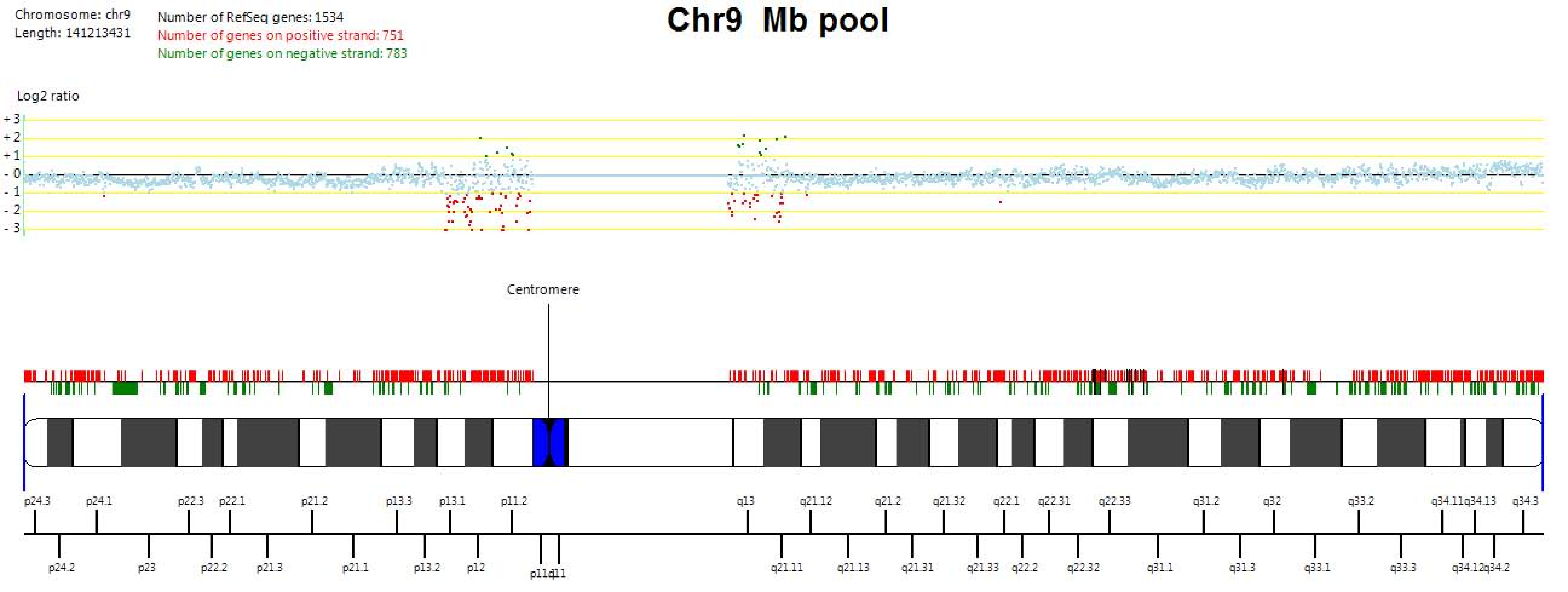
Chromosome: chr9
Length: 141213431
Number of RefSeq genes: 1534
Number of genes on positive strand: 751
Number of genes on negative strand: 783
Chr9 Mb pool
Log2 ratio
+3
+2
+1
- 0
- 1
- 2
- 3
Centromere
p24.3
p24.1
p22.3
p22.1
p21.2
p13.3
p13.1
p11.2
q13
q21.12
q21.2
q21.32
q22.1
q22.31
q22.33
q31.2
q32
q33.2
q34.11
q34.13
q34.3
p24.2
p23
p22.2
p21.3
p21.1
p13.2
p12
p11
q11
q21.11
q21.13
q21.31
q21.33
q22.2
q22.32
q31.1
q31.3
q33.1
q33.3
q34.12
q34.2

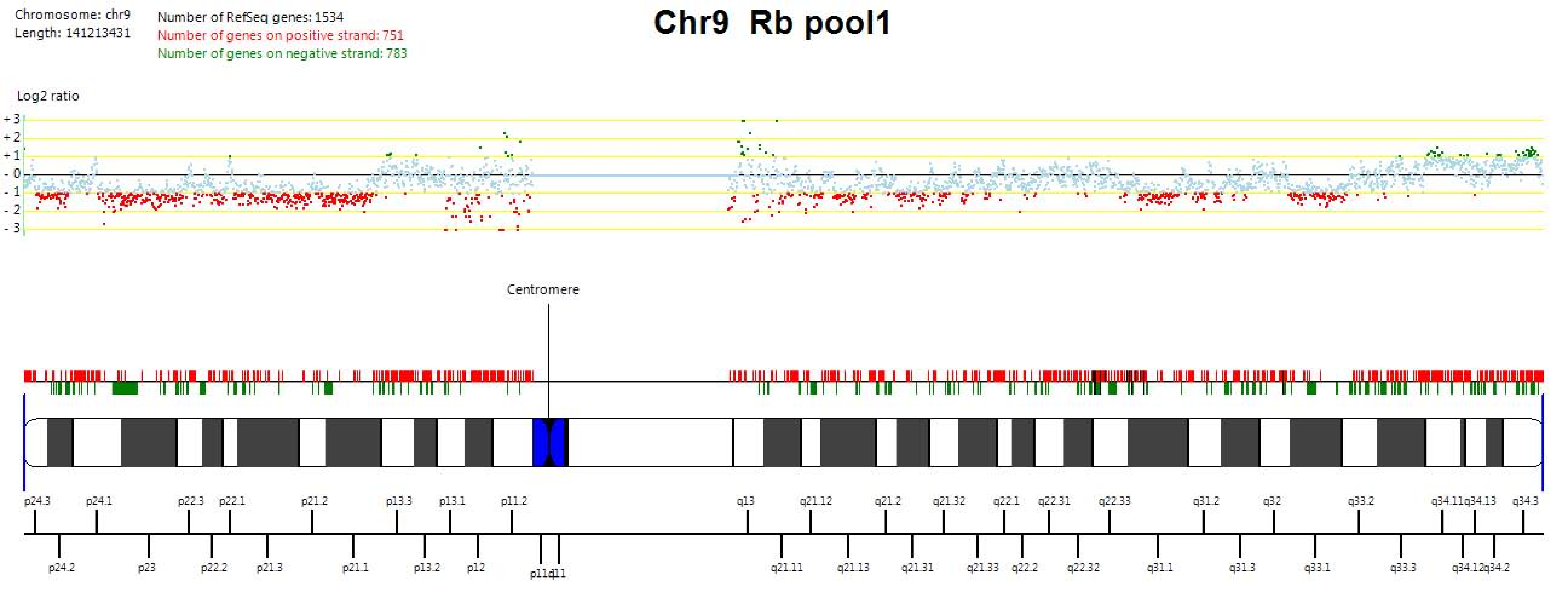
Chromosome: chr9
Length: 141213431
Number of RefSeq genes: 1534
Number of genes on positive strand: 751
Number of genes on negative strand: 783
Chr9 Rb pool1
Log2 ratio
+3
+2
+1
- 0
- 1
- 2
- 3
Centromere
p24.3
p24.1
p22.3
p22.1
p21.2
p13.3
p13.1
p11.2
q13
q21.12
q21.2
q21.32
q22.1
q22.31
q22.33
q31.2
q32
q33.2
q34.11
q34.13
q34.3
p24.2
p23
p22.2
p21.3
p21.1
p13.2
p12
p11.1
q11
q21.11
q21.13
q21.31
q21.33
q22.2
q22.32
q31.1
q31.3
q33.1
q33.3
q34.12
q34.2

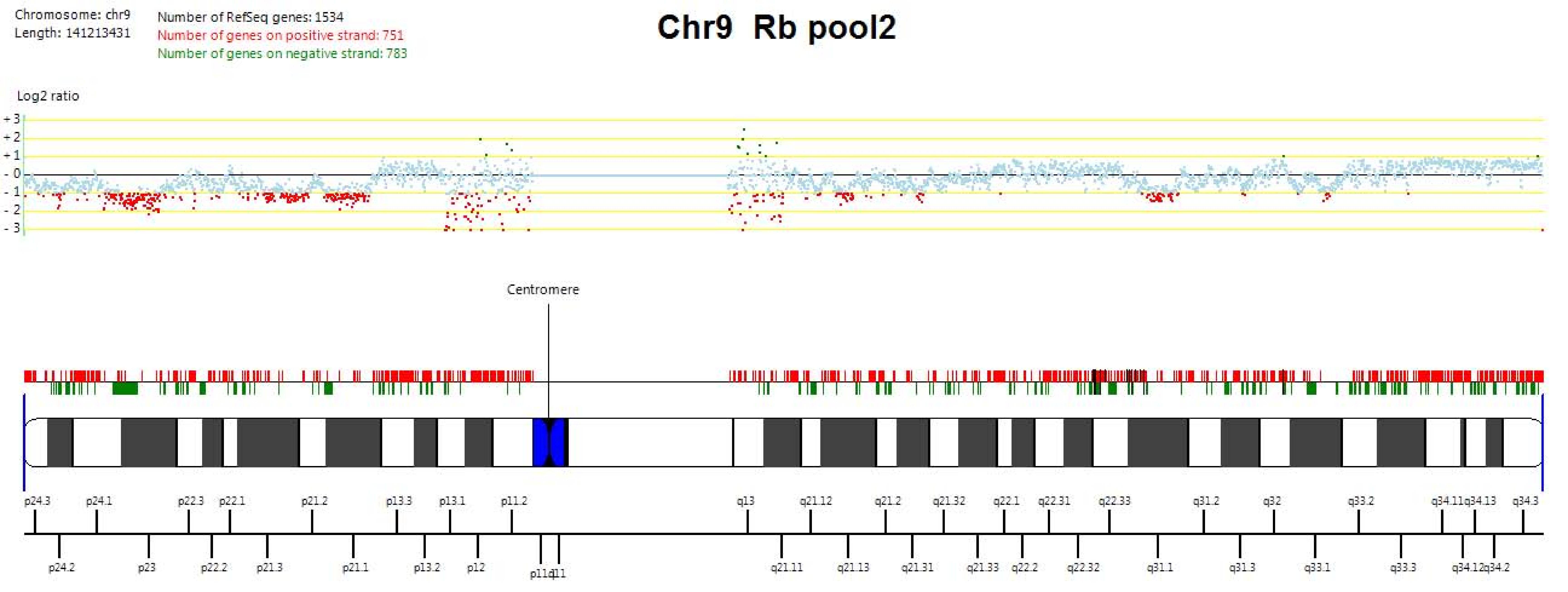

Chromosome: chr9
Length: 141213431
Number of RefSeq genes: 1534
Number of genes on positive strand: 751
Number of genes on negative strand: 783
Chr9 Rb pool2
Log2 ratio
+3
+2
+1
-0
-1
-2
-3
Centromere
p24.3
p24.1
p22.3
p22.1
p21.2
p13.3
p13.1
p11.2
q13
q21.12
q21.2
q21.32
q22.1
q22.31
q22.33
q31.2
q32
q33.2
q34.11
q34.13
q34.3
p24.2
p23
p22.2
p21.3
p21.1
p13.2
p12
p11.1
q11
q21.11
q21.13
q21.31
q21.33
q22.2
q22.32
q31.1
q31.3
q33.1
q33.3
q34.12
q34.2

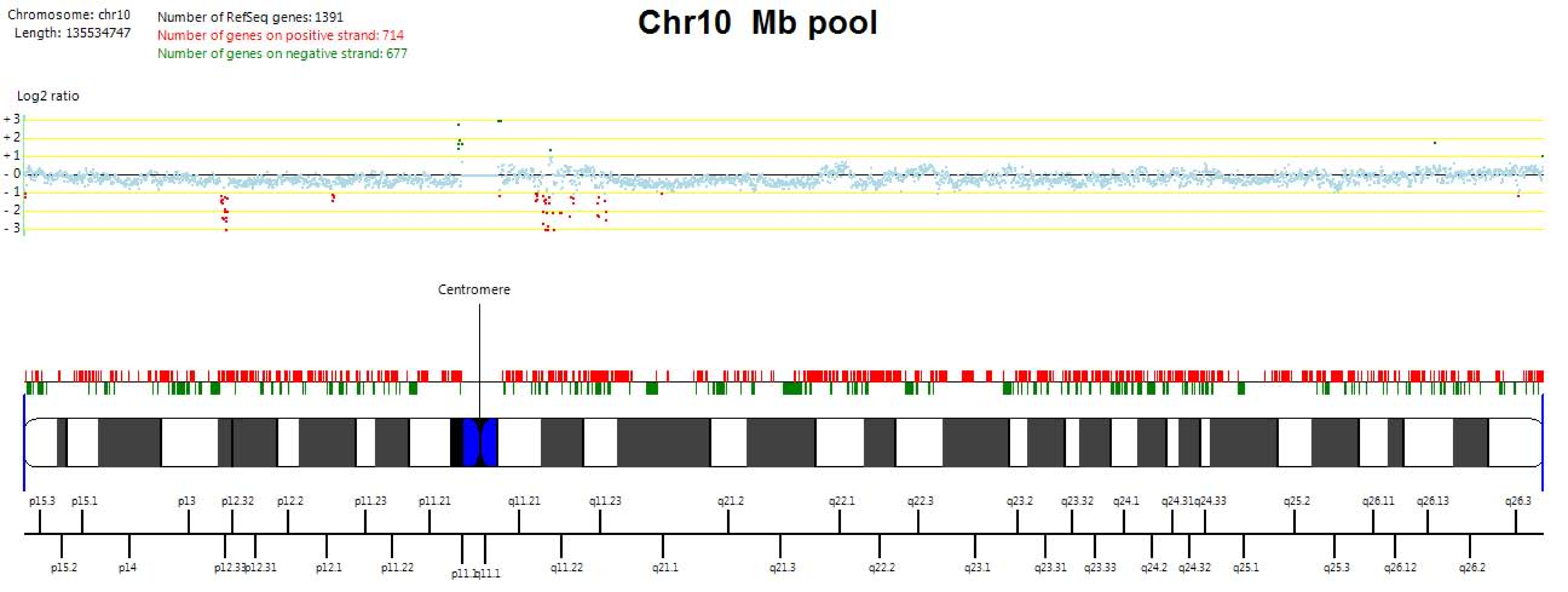
Chromosome: chr10
Length: 135534747
Number of RefSeq genes: 1391
Number of genes on positive strand: 714
Number of genes on negative strand: 677
Chr10 Mb pool
Log2 ratio
+3
+2
+1
- 0
- 1
- 2
- 3
Centromere
p15.3 p15.1 p13 p12.32 p12.2 p11.23 p11.21 q11.21 q11.23 q21.2 q22.1 q22.3 q23.2 q23.32 q24.1 q24.31 q24.33 q25.2 q26.11 q26.13 q26.3
p15.2 p14 p12.33 p12.31 p12.1 p11.22 p11.1 q11.1 q11.22 q21.1 q21.3 q22.2 q23.1 q23.31 q23.33 q24.2 q24.32 q25.1 q25.3 q26.12 q26.2

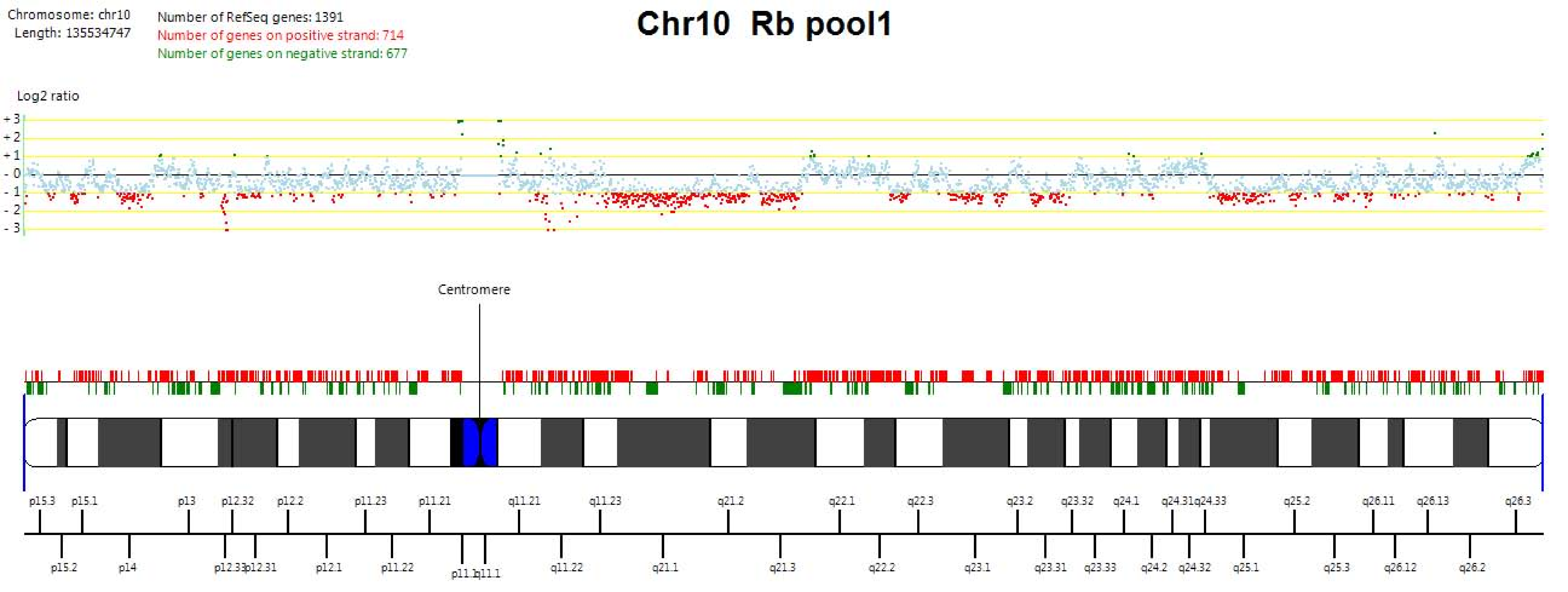
Chr10 Rb pool1
Chromosome: chr10
Length: 135534747
Number of RefSeq genes: 1391
Number of genes on positive strand: 714
Number of genes on negative strand: 677
Log2 ratio
+3
+2
+1
- 0
- 1
- 2
- 3
Centromere
p15.3
p15.1
p13
p12.32
p12.2
p11.23
p11.21
q11.21
q11.23
q21.2
q22.1
q22.3
q23.2
q23.32
q24.1
q24.31
q24.33
q25.2
q26.11
q26.13
q26.3
p15.2
p14
p12.33
p12.31
p12.1
p11.22
q11.22
q21.1
q21.3
q22.2
q23.1
q23.31
q23.33
q24.2
q24.32
q25.1
q25.3
q26.12
q26.2

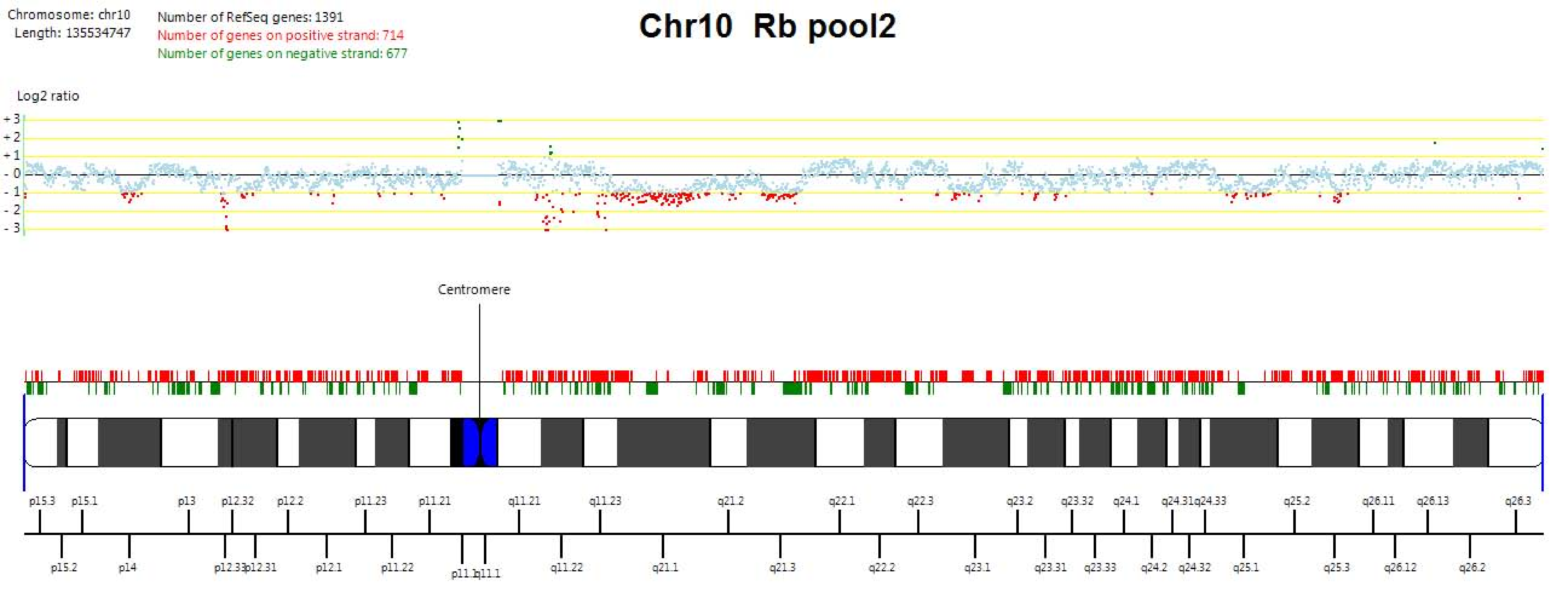

Chromosome: chr10
Length: 135534747
Number of RefSeq genes: 1391
Number of genes on positive strand: 714
Number of genes on negative strand: 677
Chr10 Rb pool2
Log2 ratio
+3
+2
+1
- 0
- 1
- 2
- 3
Centromere
p15.3 p15.1 p13 p12.32 p12.2 p11.23 p11.21 q11.21 q11.23 q21.2 q22.1 q22.3 q23.2 q23.32 q24.1 q24.31 q24.33 q25.2 q26.11 q26.13 q26.3
p15.2 p14 p12.33 p12.31 p12.1 p11.22 p11.1 q11.1 q11.22 q21.1 q21.3 q22.2 q23.1 q23.31 q23.33 q24.2 q24.32 q25.1 q25.3 q26.12 q26.2

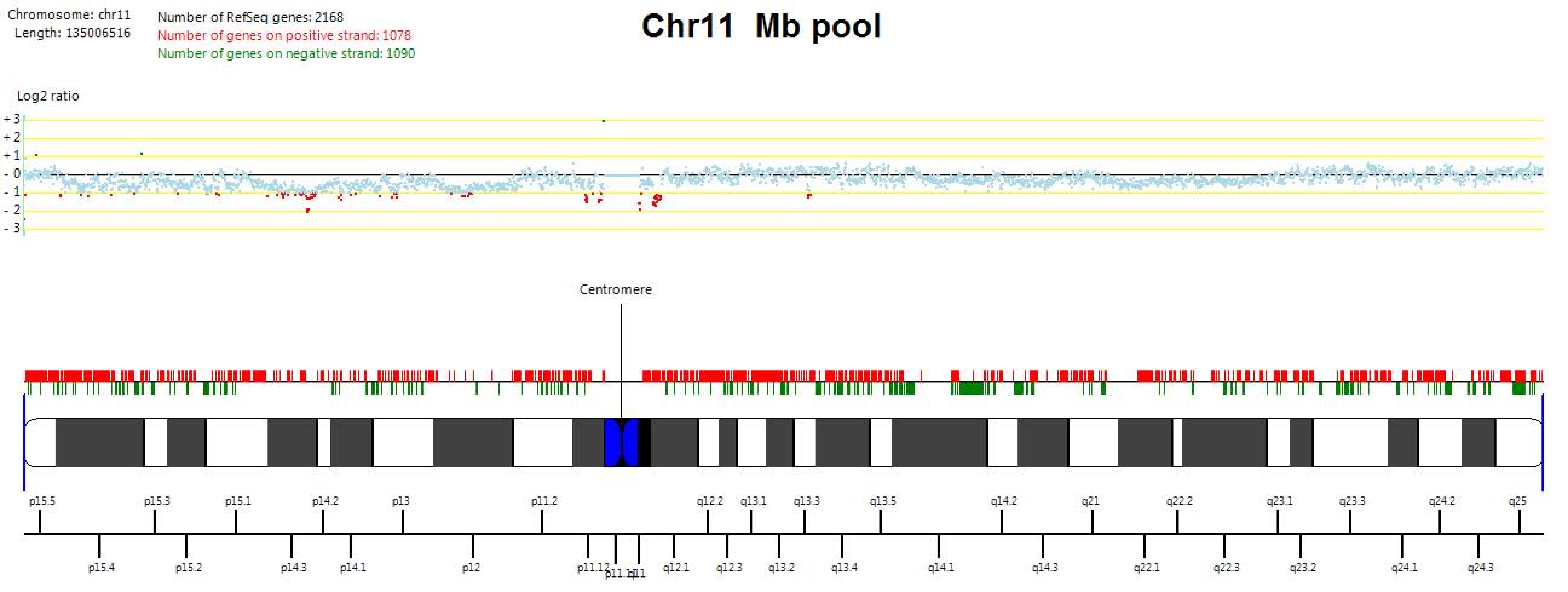

Chromosome: chr11
Length: 135006516
Number of RefSeq genes: 2168
Number of genes on positive strand: 1078
Number of genes on negative strand: 1090
Chr11 Mb pool
Log2 ratio
+3
+2
+1
- 0
- 1
- 2
- 3
Centromere
p15.5
p15.3
p15.1
p14.2
p13
p11.2
q12.2
q13.1
q13.3
q13.5
q14.2
q21
q22.2
q23.1
q23.3
q24.2
q25
p15.4
p15.2
p14.3
p14.1
p12
p11.12
q12.1
q12.3
q13.2
q13.4
q14.1
q14.3
q22.1
q22.3
q23.2
q24.1
q24.3

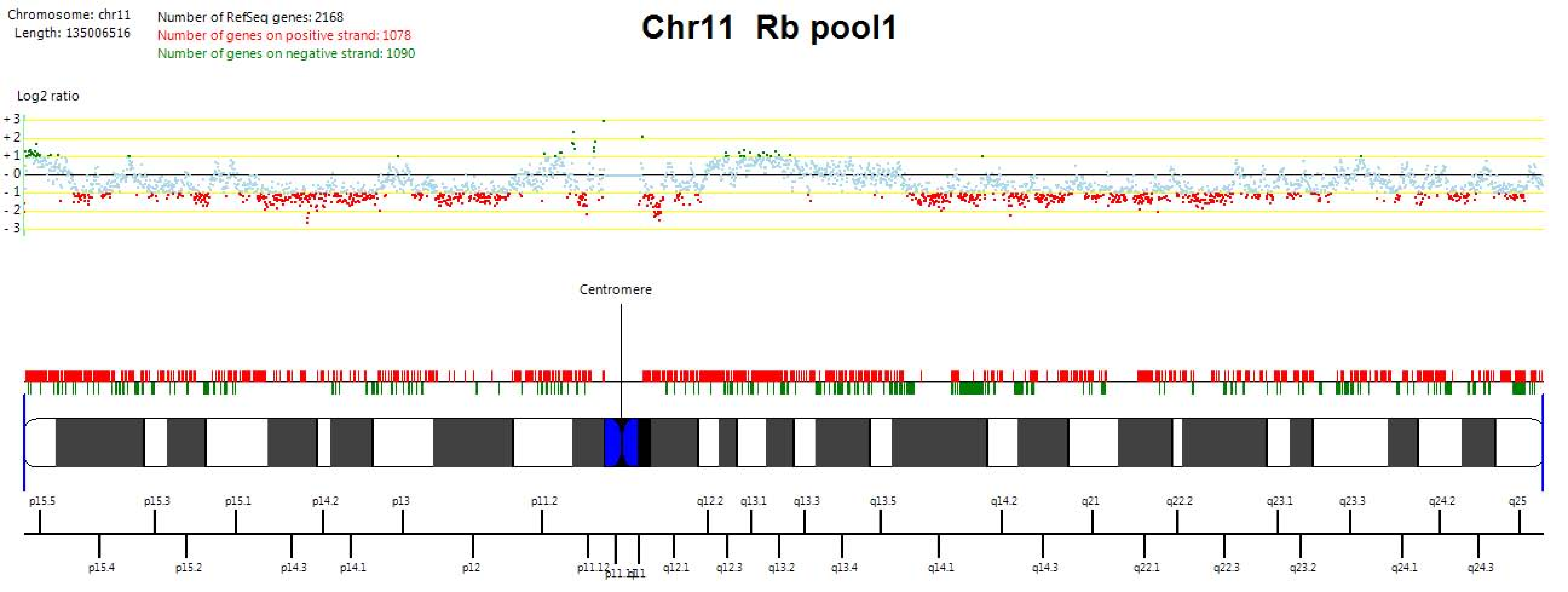
Chromosome: chr11
Length: 135006516
Number of RefSeq genes: 2168
Number of genes on positive strand: 1078
Number of genes on negative strand: 1090
Chr11 Rb pool1
Log2 ratio
+3
+2
+1
-0
-1
-2
-3
Centromere
p15.5
p15.3
p15.1
p14.2
p13
p11.2
q12.2
q13.1
q13.3
q13.5
q14.2
q21
q22.2
q23.1
q23.3
q24.2
q25
p15.4
p15.2
p14.3
p14.1
p12
p11.12
p11.11
q11
q12.1
q12.3
q13.2
q13.4
q14.1
q14.3
q22.1
q22.3
q23.2
q24.1
q24.3

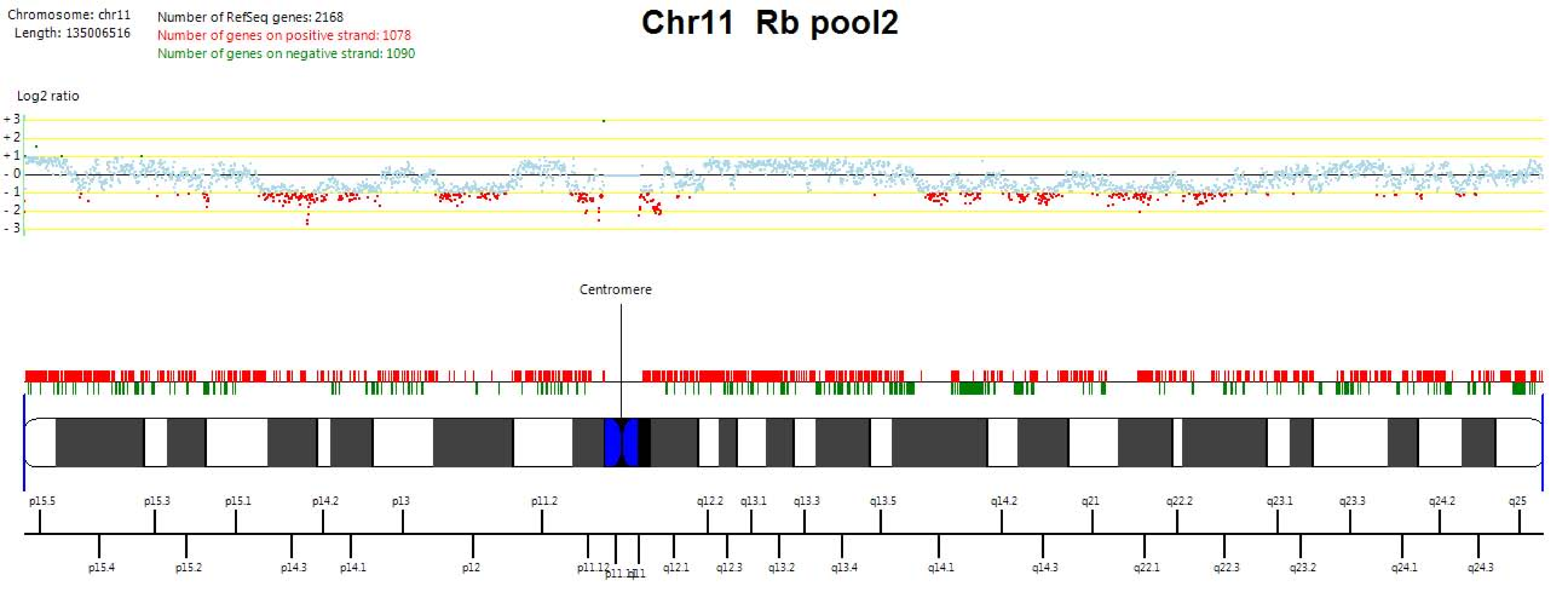
Chromosome: chr11
Length: 135006516
Number of RefSeq genes: 2168
Number of genes on positive strand: 1078
Number of genes on negative strand: 1090
Chr11 Rb pool2
Log2 ratio
+3
+2
+1
- 0
- 1
- 2
- 3
Centromere
p15.5
p15.3
p15.1
p14.2
p13
p11.2
q12.2
q13.1
q13.3
q13.5
q14.2
q21
q22.2
q23.1
q23.3
q24.2
q25
p15.4
p15.2
p14.3
p14.1
p12
p11.12
q11
q12.1
q12.3
q13.2
q13.4
q14.1
q14.3
q22.1
q22.3
q23.2
q24.1
q24.3

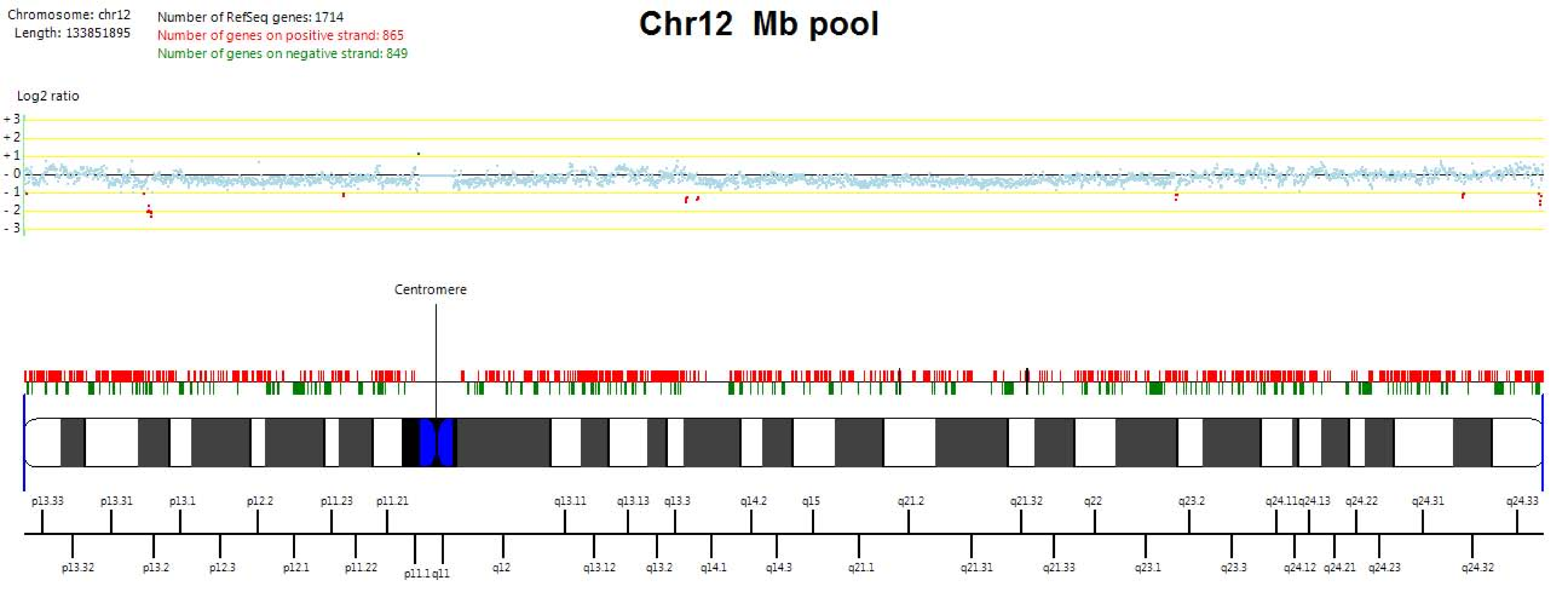
Chromosome: chr12
Length: 133851895
Number of RefSeq genes: 1714
Number of genes on positive strand: 865
Number of genes on negative strand: 849
Chr12 Mb pool
Log2 ratio
+3
+2
+1
-0
-1
-2
-3
Centromere
p13.33 p13.31 p13.1 p12.2 p11.23 p11.21 q13.11 q13.13 q13.3 q14.2 q15 q21.2 q21.32 q22 q23.2 q24.11 q24.13 q24.22 q24.31 q24.33
p13.32 p13.2 p12.3 p12.1 p11.22 p11.1 q11 q12 q13.12 q13.2 q14.1 q14.3 q21.1 q21.31 q21.33 q23.1 q23.3 q24.12 q24.21 q24.23 q24.32

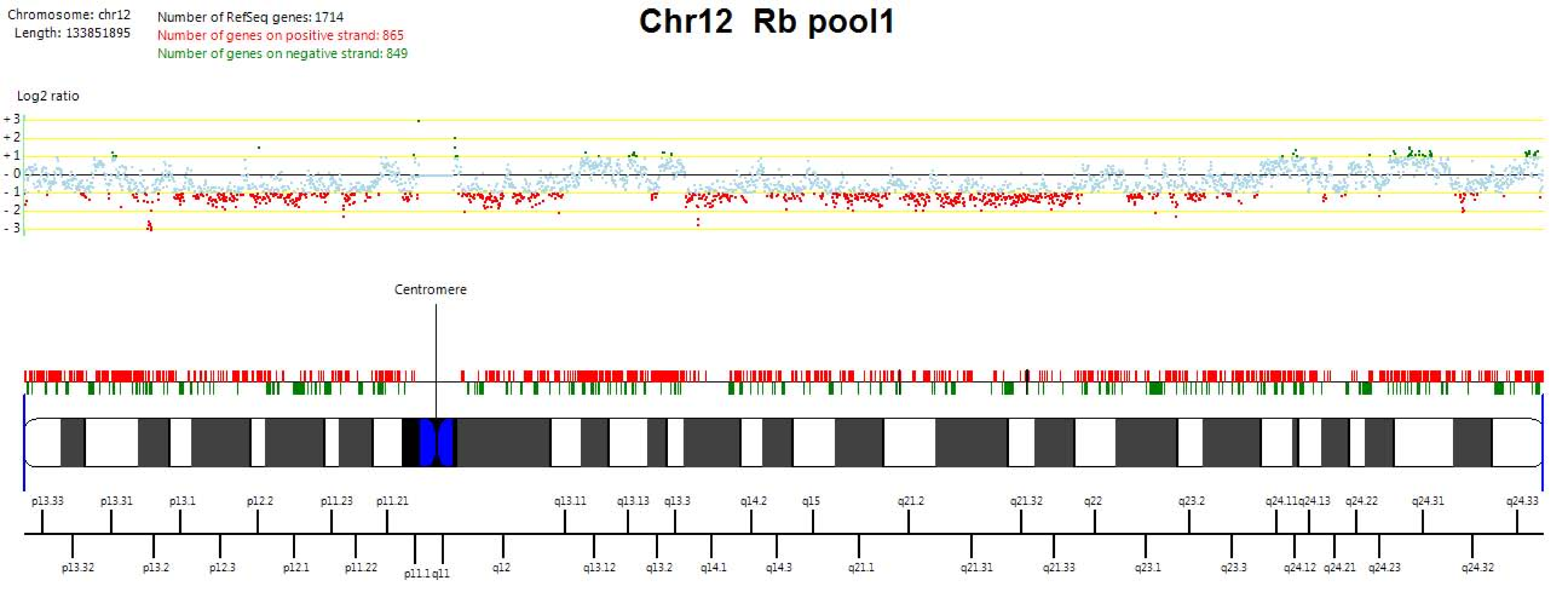
Chromosome: chr12
Length: 133851895
Number of RefSeq genes: 1714
Number of genes on positive strand: 865
Number of genes on negative strand: 849
Chr12 Rb pool1
Log2 ratio
+3
+2
+1
-0
-1
-2
-3
Centromere
p13.33
p13.31
p13.1
p12.2
p11.23
p11.21
q13.11
q13.13
q13.3
q14.2
q15
q21.2
q21.32
q22
q23.2
q24.11
q24.13
q24.22
q24.31
q24.33
p13.32
p13.2
p12.3
p12.1
p11.22
p11.1
q11
q12
q13.12
q13.2
q14.1
q14.3
q21.1
q21.31
q21.33
q23.1
q23.3
q24.12
q24.21
q24.23
q24.32

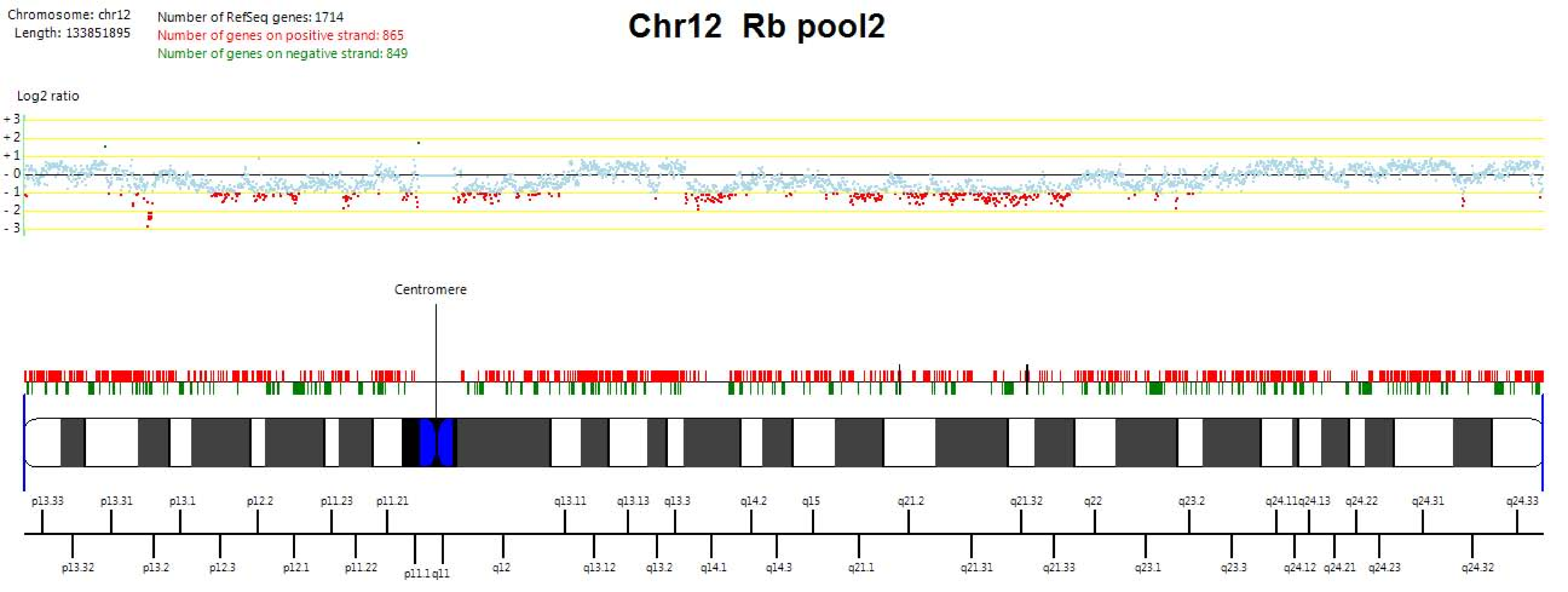

Chromosome: chr12
Length: 133851895
Number of RefSeq genes: 1714
Number of genes on positive strand: 865
Number of genes on negative strand: 849
Chr12 Rb pool2
Log2 ratio
+3
+2
+1
-0
-1
-2
-3
Centromere
p13.33
p13.31
p13.1
p12.2
p11.23
p11.21
q13.11
q13.13
q13.3
q14.2
q15
q21.2
q21.32
q22
q23.2
q24.11
q24.13
q24.22
q24.31
q24.33
p13.32
p13.2
p12.3
p12.1
p11.22
p11.1
q11
q12
q13.12
q13.2
q14.1
q14.3
q21.1
q21.31
q21.33
q23.1
q23.3
q24.12
q24.21
q24.23
q24.32

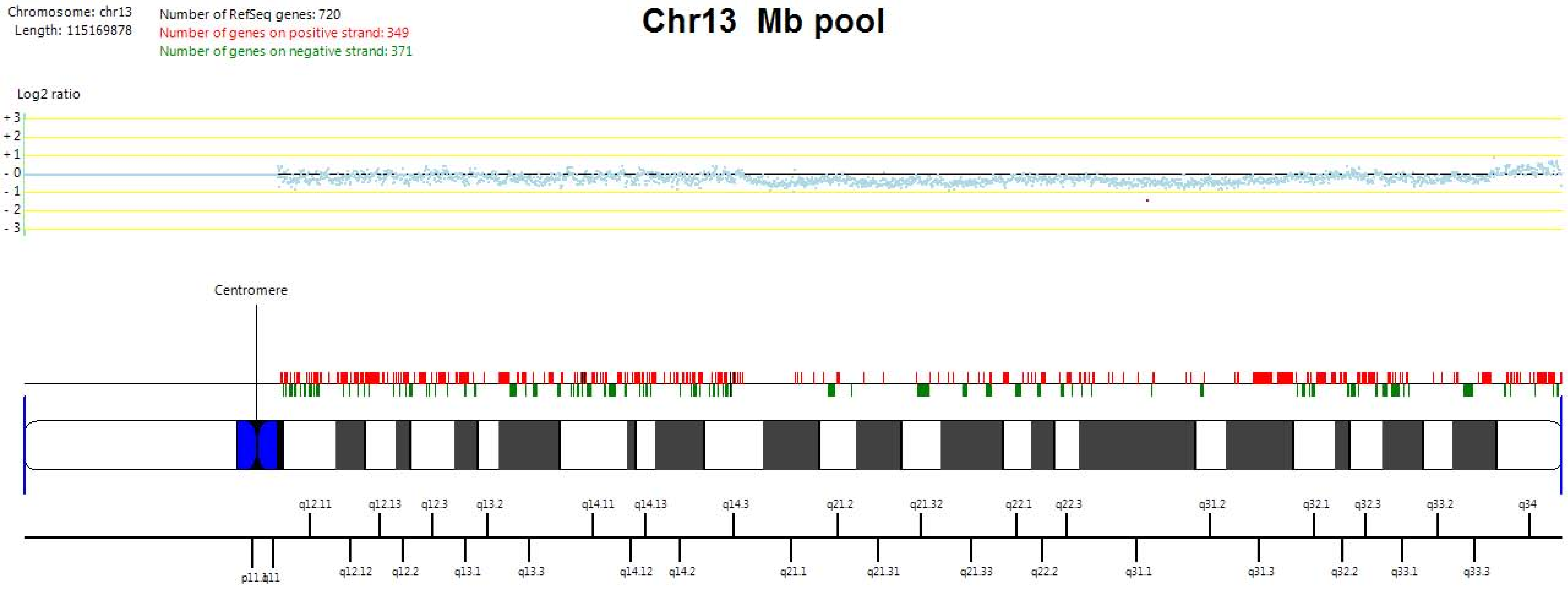

Chromosome: chr13
Length: 115169878
Number of RefSeq genes: 720
Number of genes on positive strand: 349
Number of genes on negative strand: 371
Chr13 Mb pool
Log2 ratio
+3
+2
+1
- 0
- 1
- 2
- 3
Centromere
q12.11
q12.13
q12.3
q13.2
q14.11
q14.13
q14.3
q21.2
q21.32
q22.1
q22.3
q31.2
q32.1
q32.3
q33.2
q34
p11.1
q11
q12.12
q12.2
q13.1
q13.3
q14.12
q14.2
q21.1
q21.31
q21.33
q22.2
q31.1
q31.3
q32.2
q33.1
q33.3

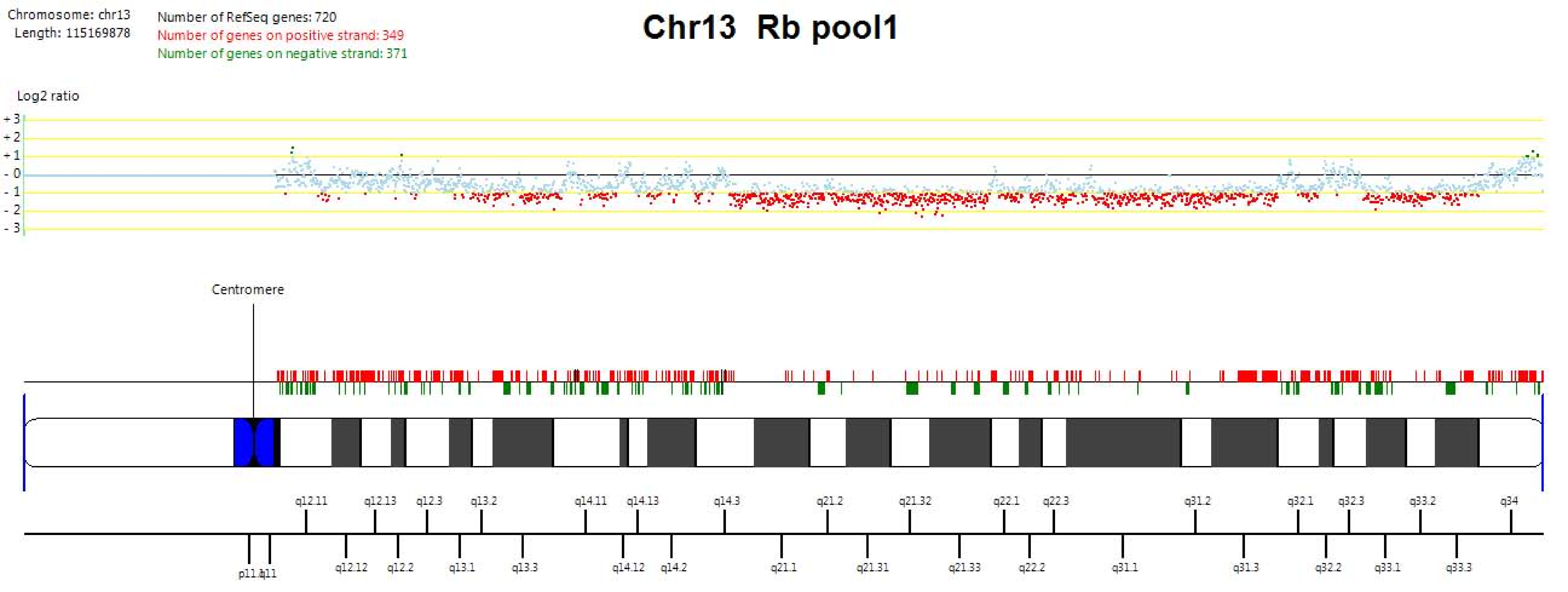
Chromosome: chr13
Length: 115169878
Number of RefSeq genes: 720
Number of genes on positive strand: 349
Number of genes on negative strand: 371
Chr13 Rb pool1
Log2 ratio
+3
+2
+1
-0
-1
-2
-3
Centromere
q12.11
q12.13
q12.3
q13.2
q14.11
q14.13
q14.3
q21.2
q21.32
q22.1
q22.3
q31.2
q32.1
q32.3
q33.2
q34
p11.1
q11
q12.12
q12.2
q13.1
q13.3
q14.12
q14.2
q21.1
q21.31
q21.33
q22.2
q31.1
q31.3
q32.2
q33.1
q33.3

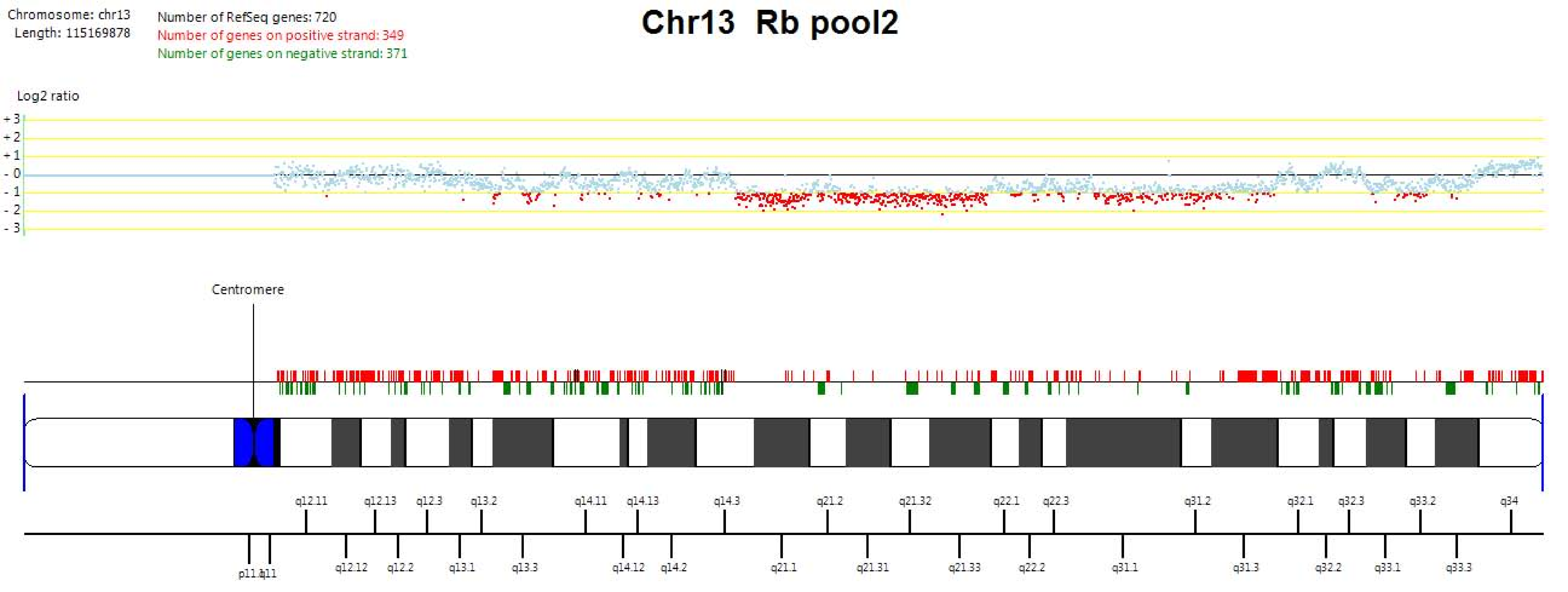

Chromosome: chr13
Length: 115169878
Number of RefSeq genes: 720
Number of genes on positive strand: 349
Number of genes on negative strand: 371
Chr13 Rb pool2
Log2 ratio
+3
+2
+1
- 0
- 1
- 2
- 3
Centromere
q12.11
q12.13
q12.3
q13.2
q14.11
q14.13
q14.3
q21.2
q21.32
q22.1
q22.3
q31.2
q32.1
q32.3
q33.2
q34
p11.1
q11
q12.12
q12.2
q13.1
q13.3
q14.12
q14.2
q21.1
q21.31
q21.33
q22.2
q31.1
q31.3
q32.2
q33.1
q33.3

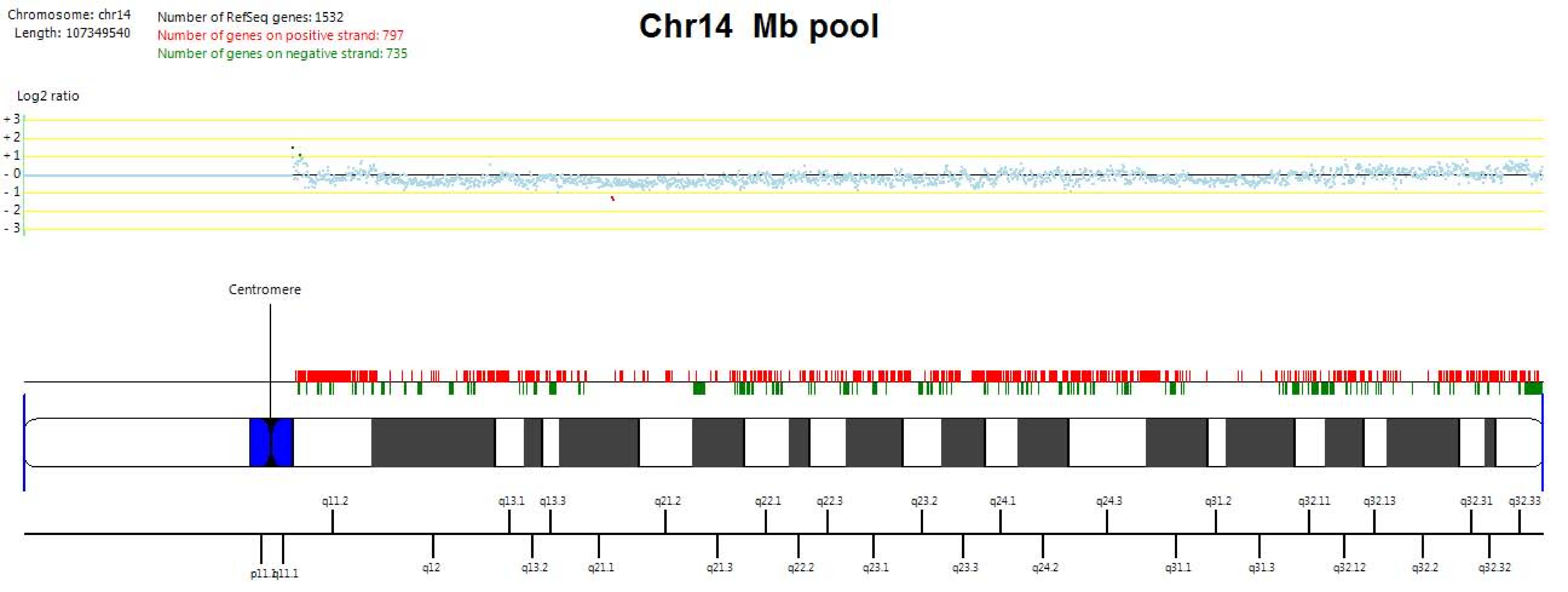

Chromosome: chr14
Length: 107349540
Number of RefSeq genes: 1532
Number of genes on positive strand: 797
Number of genes on negative strand: 735
Chr14 Mb pool
Log2 ratio
+3
+2
+1
- 0
- 1
- 2
- 3
Centromere
q11.2
q13.1
q13.3
q21.2
q22.1
q22.3
q23.2
q24.1
q24.3
q31.2
q32.11
q32.13
q32.31
q32.33
p11.1
q11.1
q12
q13.2
q21.1
q21.3
q22.2
q23.1
q23.3
q24.2
q31.1
q31.3
q32.12
q32.2
q32.32

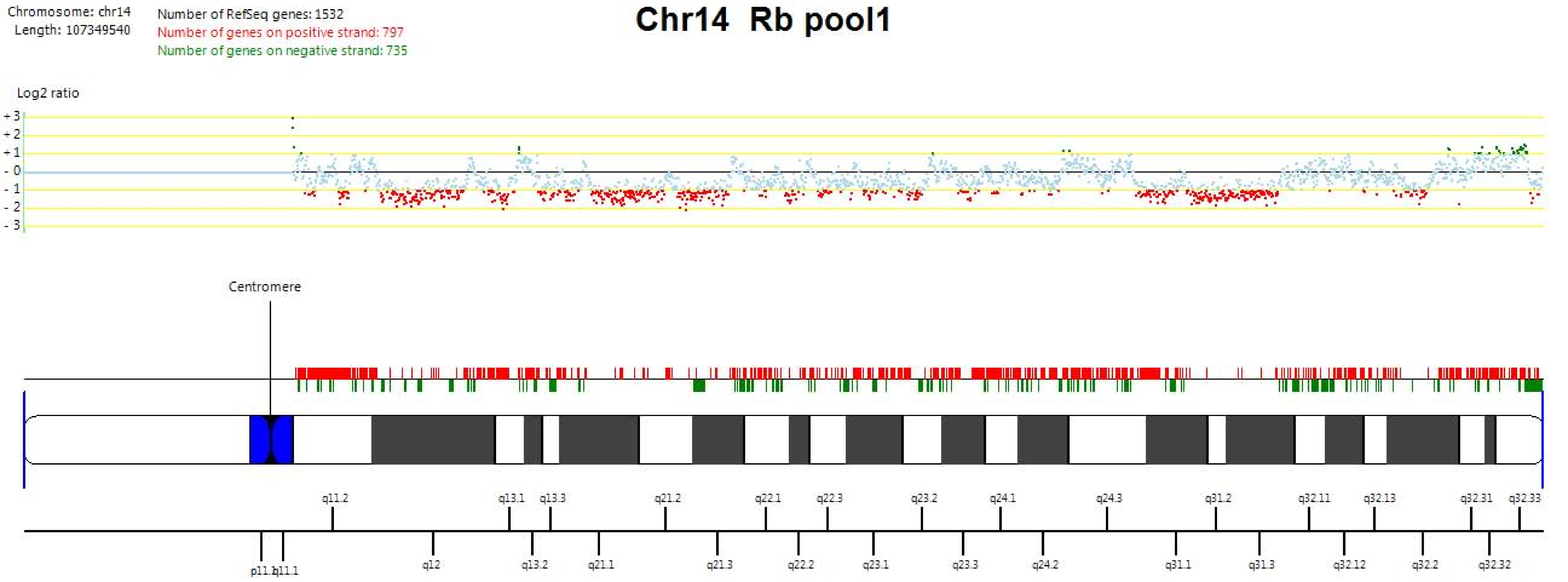

Chromosome: chr14
Length: 107349540
Number of RefSeq genes: 1532
Number of genes on positive strand: 797
Number of genes on negative strand: 735
Chr14 Rb pool1
Log2 ratio
+3
+2
+1
-0
-1
-2
-3
Centromere
q11.2
q13.1
q13.3
q21.2
q22.1
q22.3
q23.2
q24.1
q24.3
q31.2
q32.11
q32.13
q32.31
q32.33
p11.1
q11.1
q12
q13.2
q21.1
q21.3
q22.2
q23.1
q23.3
q24.2
q31.1
q31.3
q32.12
q32.2
q32.32

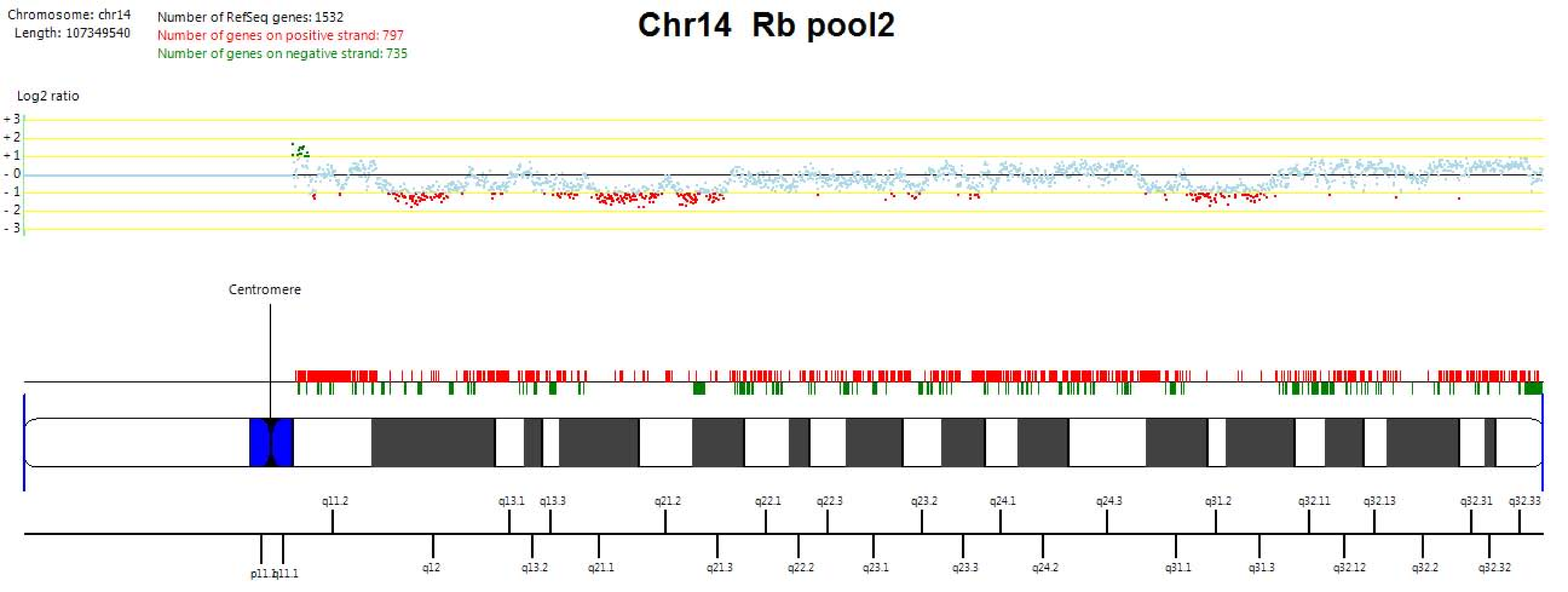
Chromosome: chr14
Length: 107349540
Number of RefSeq genes: 1532
Number of genes on positive strand: 797
Number of genes on negative strand: 735
Chr14 Rb pool2
Log2 ratio
+3
+2
+1
-0
-1
-2
-3
Centromere
q11.2
q13.1
q13.3
q21.2
q22.1
q22.3
q23.2
q24.1
q24.3
q31.2
q32.11
q32.13
q32.31
q32.33
p11.1
q11.1
q12
q13.2
q21.1
q21.3
q22.2
q23.1
q23.3
q24.2
q31.1
q31.3
q32.12
q32.2
q32.32

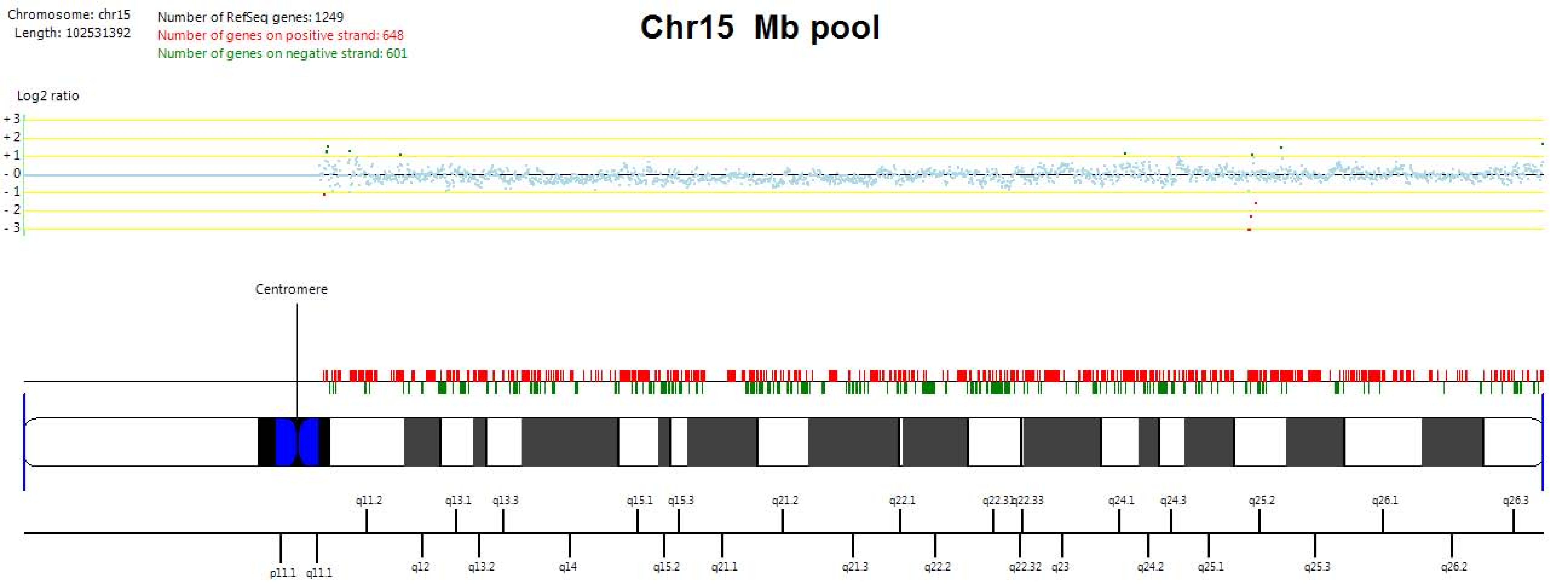

Chromosome: chr15
Length: 102531392
Number of RefSeq genes: 1249
Number of genes on positive strand: 648
Number of genes on negative strand: 601
Chr15 Mb pool
Log2 ratio
+3
+2
+1
-0
-1
-2
-3
Centromere
q11.2
q13.1
q13.3
q15.1
q15.3
q21.2
q22.1
q22.31
q22.33
q24.1
q24.3
q25.2
q26.1
q26.3
p11.1
q11.1
q12
q13.2
q14
q15.2
q21.1
q21.3
q22.2
q22.32
q23
q24.2
q25.1
q25.3
q26.2

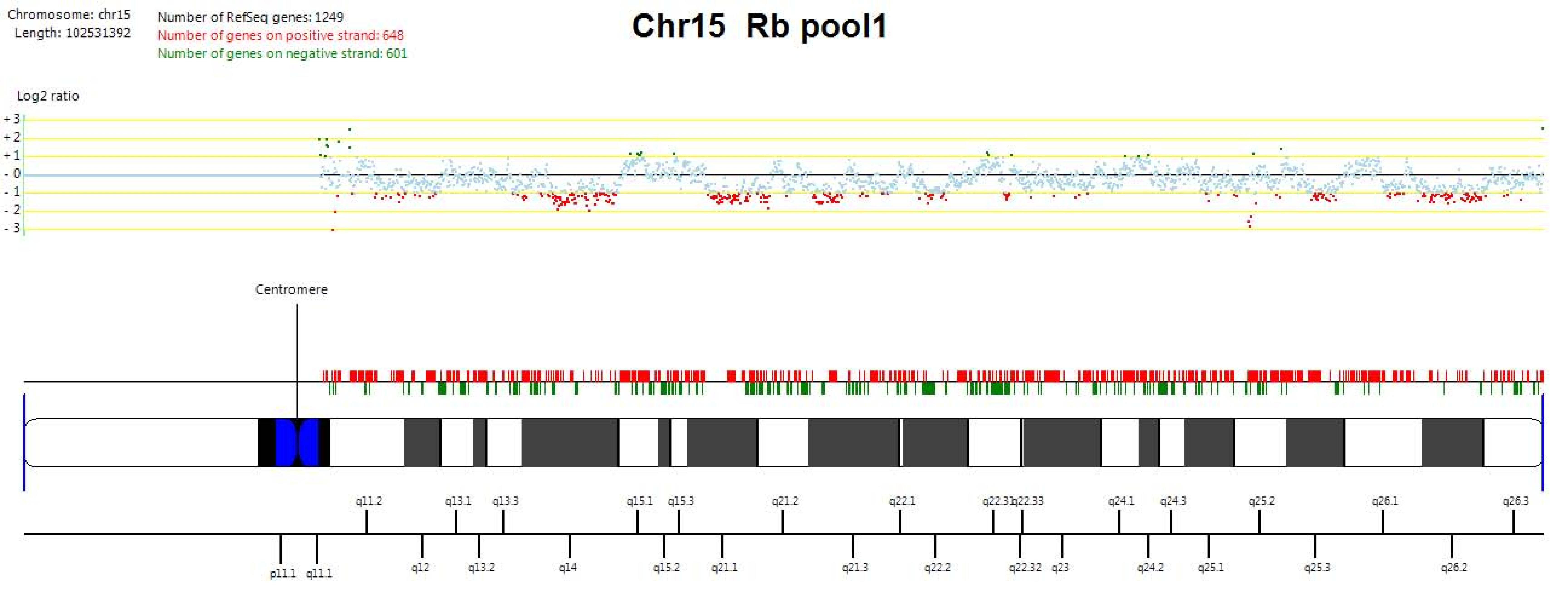
Chromosome: chr15
Length: 102531392
Number of RefSeq genes: 1249
Number of genes on positive strand: 648
Number of genes on negative strand: 601
Chr15 Rb pool1
Log2 ratio
+ 3
+ 2
+ 1
- 0
- 1
- 2
- 3
Centromere
q11.2
q13.1
q13.3
q15.1
q15.3
q21.2
q22.1
q22.31
q22.33
q24.1
q24.3
q25.2
q26.1
q26.3
p11.1
q11.1
q12
q13.2
q14
q15.2
q21.1
q21.3
q22.2
q22.32
q23
q24.2
q25.1
q25.3
q26.2

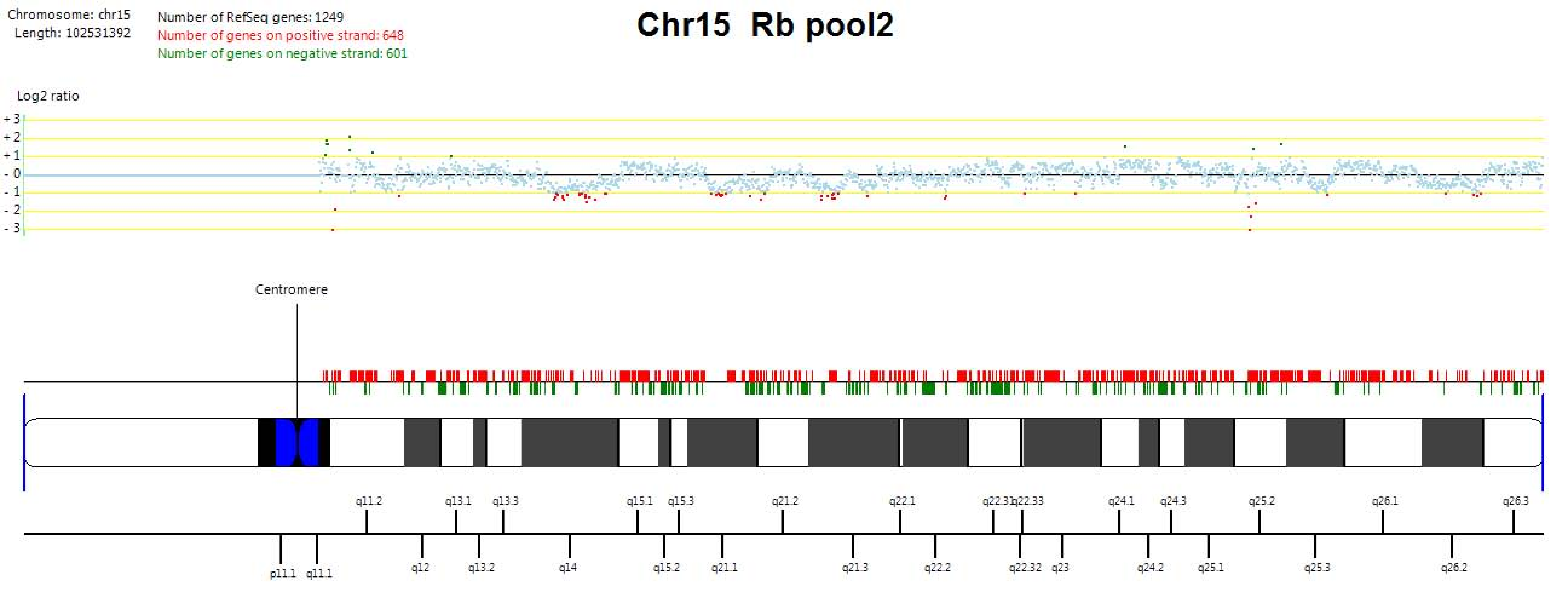
Chromosome: chr15
Length: 102531392
Number of RefSeq genes: 1249
Number of genes on positive strand: 648
Number of genes on negative strand: 601
Chr15 Rb pool2
Log2 ratio
+3
+2
+1
- 0
- 1
- 2
- 3
Centromere
q11.2
q13.1
q13.3
q15.1
q15.3
q21.2
q22.1
q22.31
q22.33
q24.1
q24.3
q25.2
q26.1
q26.3
p11.1
q11.1
q12
q13.2
q14
q15.2
q21.1
q21.3
q22.2
q22.32
q23
q24.2
q25.1
q25.3
q26.2

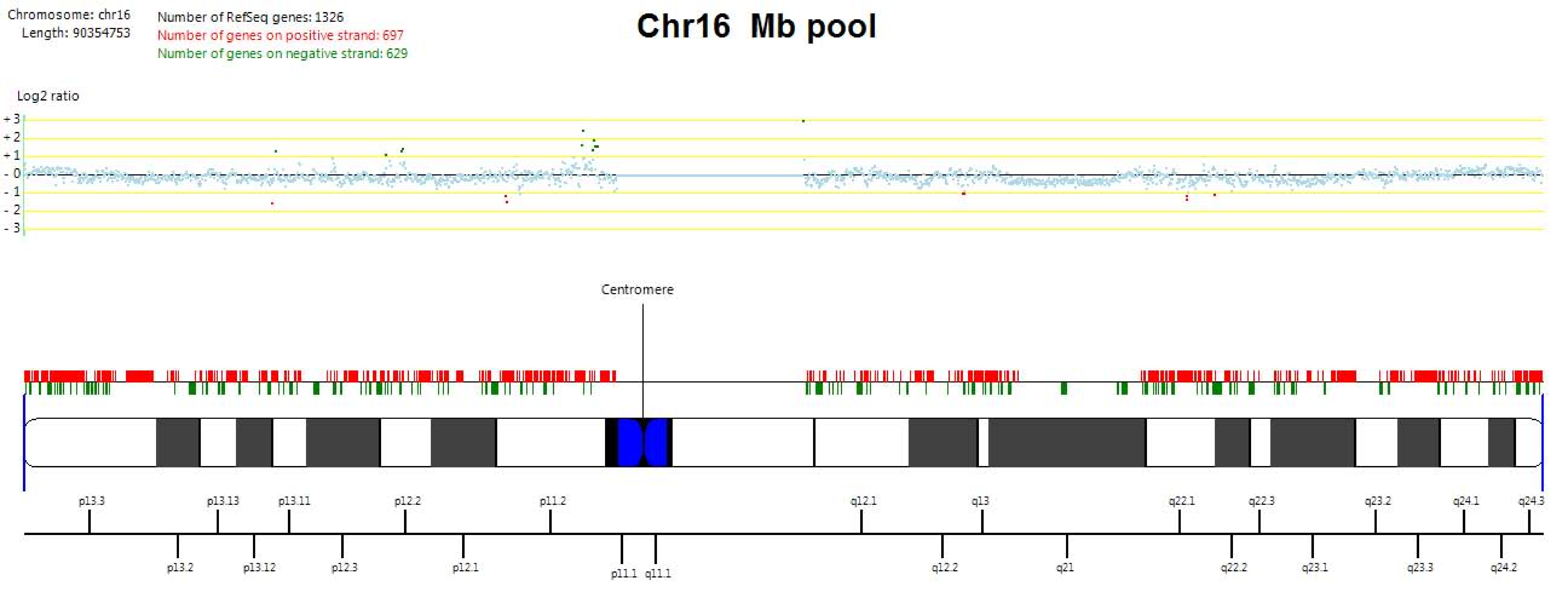
Chromosome: chr16
Length: 90354753
Number of RefSeq genes: 1326
Number of genes on positive strand: 697
Number of genes on negative strand: 629
Chr16 Mb pool
Log2 ratio
+3
+2
+1
-0
-1
-2
-3
Centromere
p13.3
p13.13
p13.11
p12.2
p11.2
q12.1
q13
q22.1
q22.3
q23.2
q24.1
q24.3
p13.2
p13.12
p12.3
p12.1
p11.1
q11.1
q12.2
q21
q22.2
q23.1
q23.3
q24.2

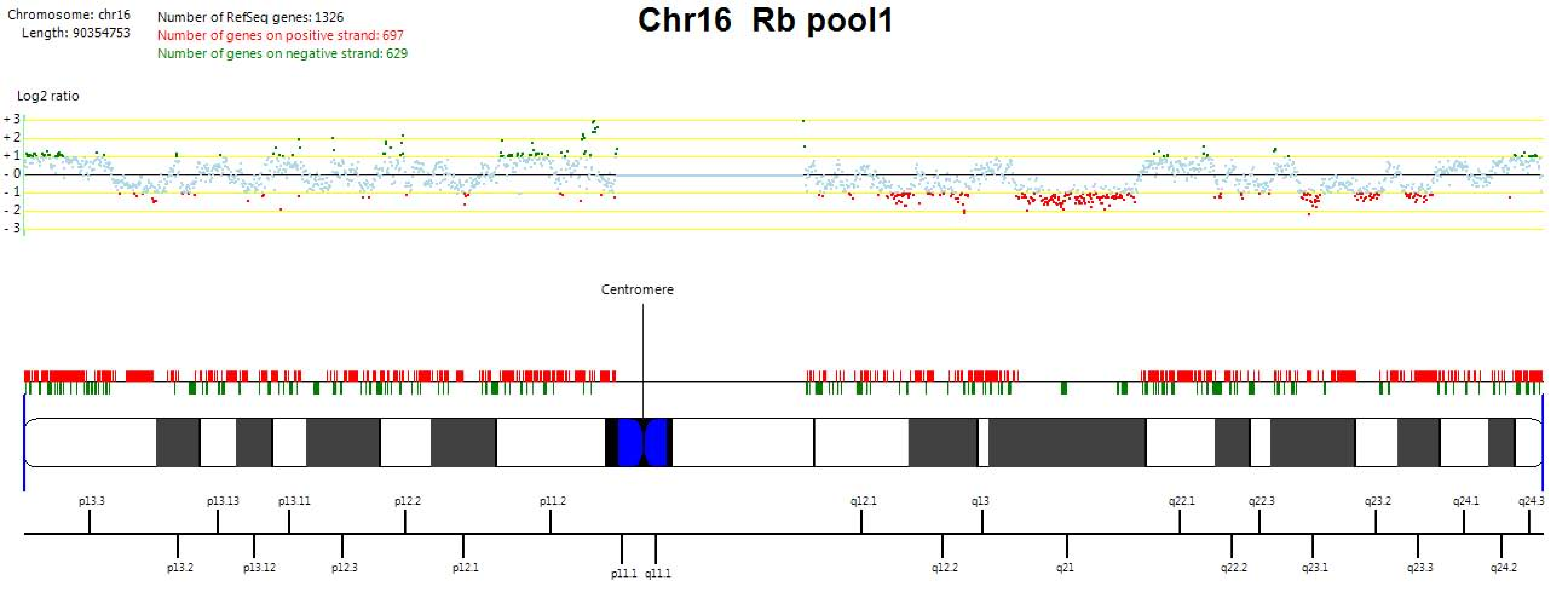

Chromosome: chr16
Length: 90354753
Number of RefSeq genes: 1326
Number of genes on positive strand: 697
Number of genes on negative strand: 629
Chr16 Rb pool1
Log2 ratio
+3
+2
+1
-0
-1
-2
-3
Centromere
p13.3
p13.13
p13.11
p12.2
p11.2
q12.1
q13
q22.1
q22.3
q23.2
q24.1
q24.3
p13.2
p13.12
p12.3
p12.1
p11.1
q11.1
q12.2
q21
q22.2
q23.1
q23.3
q24.2

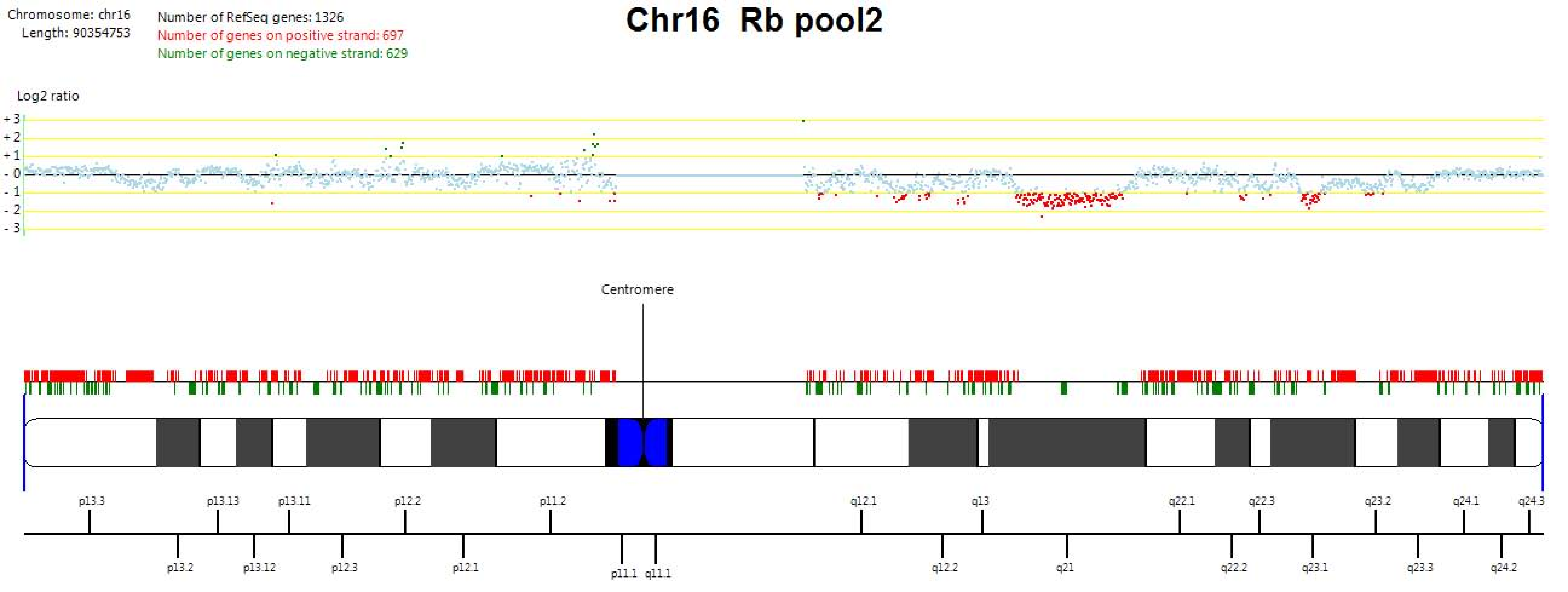
Chromosome: chr16
Length: 90354753
Number of RefSeq genes: 1326
Number of genes on positive strand: 697
Number of genes on negative strand: 629
Chr16 Rb pool2
Log2 ratio
+3
+2
+1
-0
-1
-2
-3
Centromere
p13.3
p13.2
p13.13
p13.12
p13.11
p12.3
p12.2
p12.1
p11.2
p11.1
q11.1
q12.1
q12.2
q13
q21
q22.1
q22.2
q22.3
q23.1
q23.2
q23.3
q24.1
q24.2
q24.3

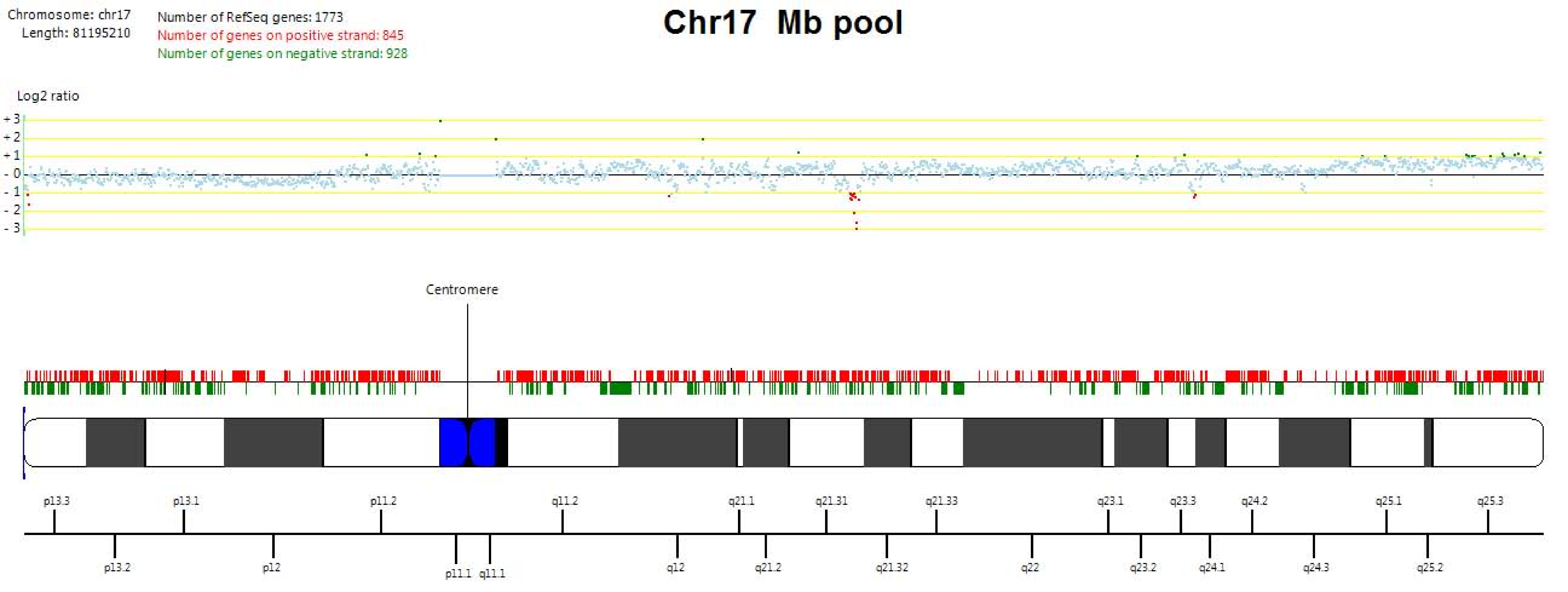
Chromosome: chr17
Length: 81195210
Number of RefSeq genes: 1773
Number of genes on positive strand: 845
Number of genes on negative strand: 928
Chr17 Mb pool
Log2 ratio
+3
+2
+1
- 0
- 1
- 2
- 3
Centromere
p13.3
p13.1
p11.2
q11.2
q21.1
q21.31
q21.33
q23.1
q23.3
q24.2
q25.1
q25.3
p13.2
p12
p11.1
q11.1
q12
q21.2
q21.32
q22
q23.2
q24.1
q24.3
q25.2

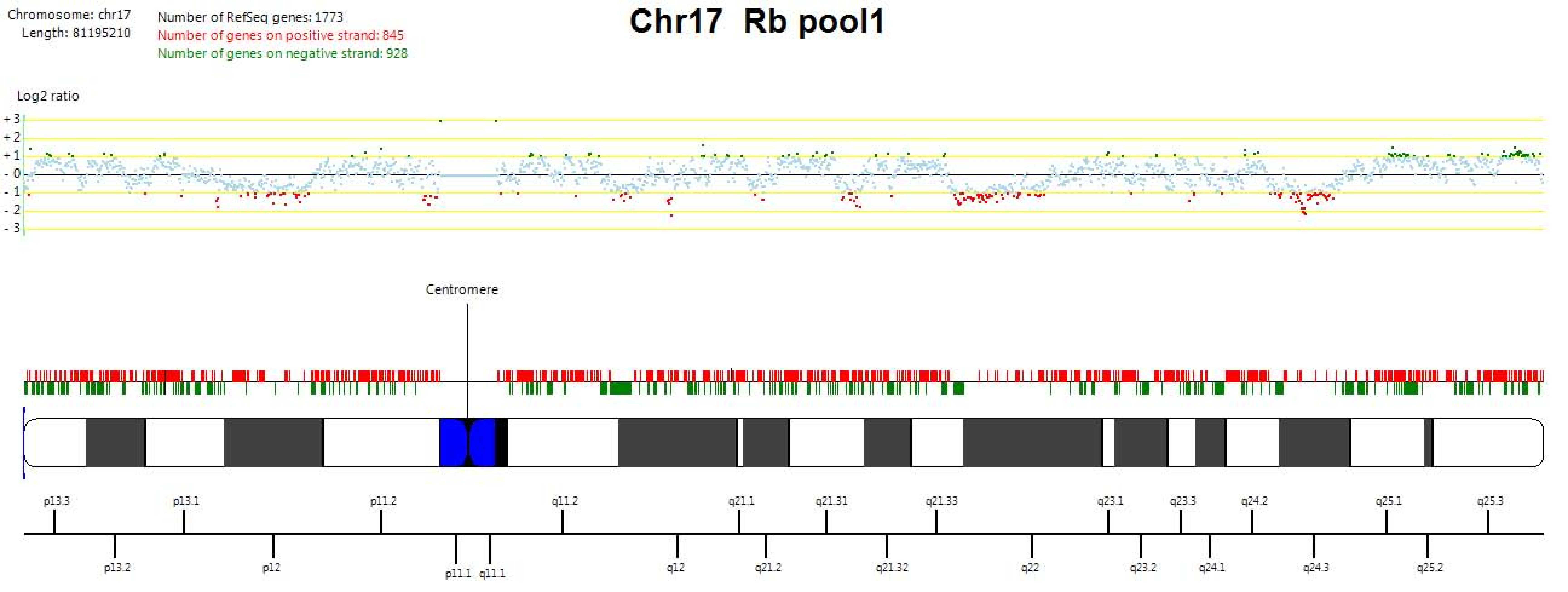

Chromosome: chr17
Length: 81195210
Number of RefSeq genes: 1773
Number of genes on positive strand: 845
Number of genes on negative strand: 928
Chr17 Rb pool1
Log2 ratio
+3
+2
+1
-0
-1
-2
-3
Centromere
p13.3
p13.1
p11.2
q11.2
q21.1
q21.31
q21.33
q23.1
q23.3
q24.2
q25.1
q25.3
p13.2
p12
p11.1
q11.1
q12
q21.2
q21.32
q22
q23.2
q24.1
q24.3
q25.2

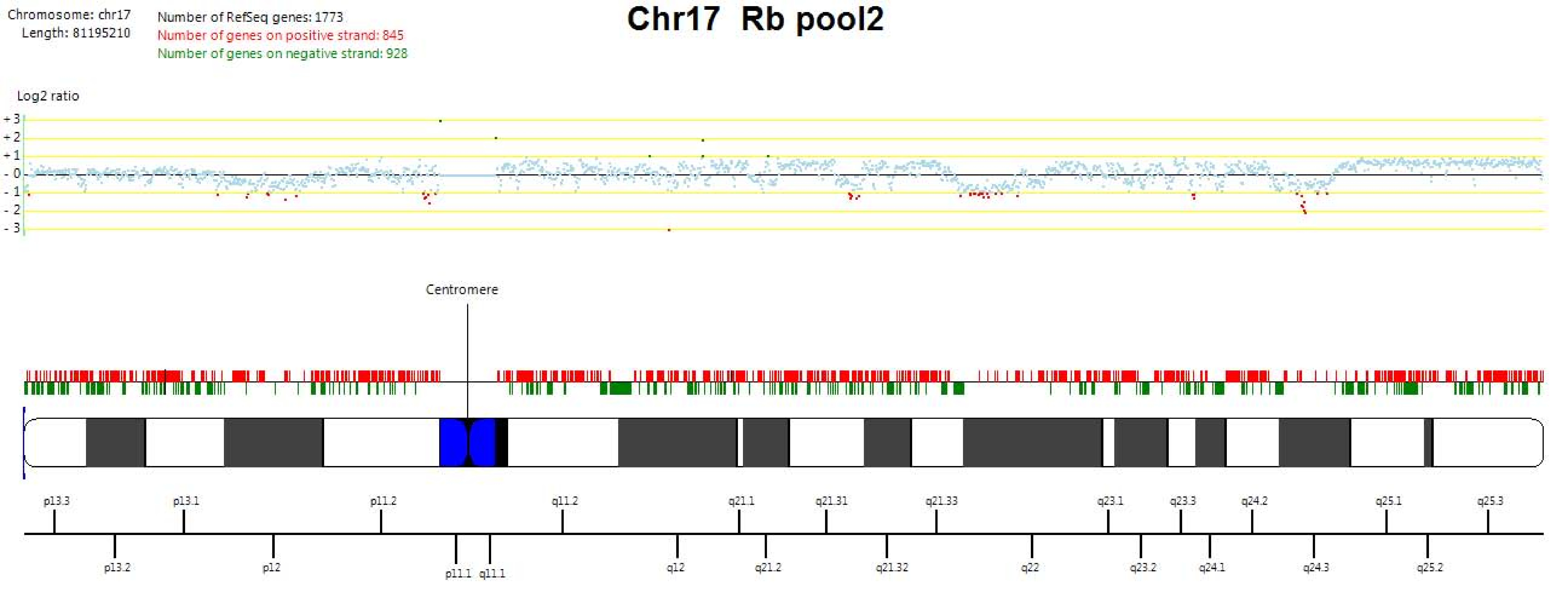

Chromosome: chr17
Length: 81195210
Number of RefSeq genes: 1773
Number of genes on positive strand: 845
Number of genes on negative strand: 928
Chr17 Rb pool2
Log2 ratio
+3
+2
+1
- 0
- 1
- 2
- 3
Centromere
p13.3
p13.2
p13.1
p12
p11.2
p11.1
q11.1
q11.2
q12
q21.1
q21.2
q21.31
q21.32
q21.33
q22
q23.1
q23.2
q23.3
q24.1
q24.2
q24.3
q25.1
q25.2
q25.3

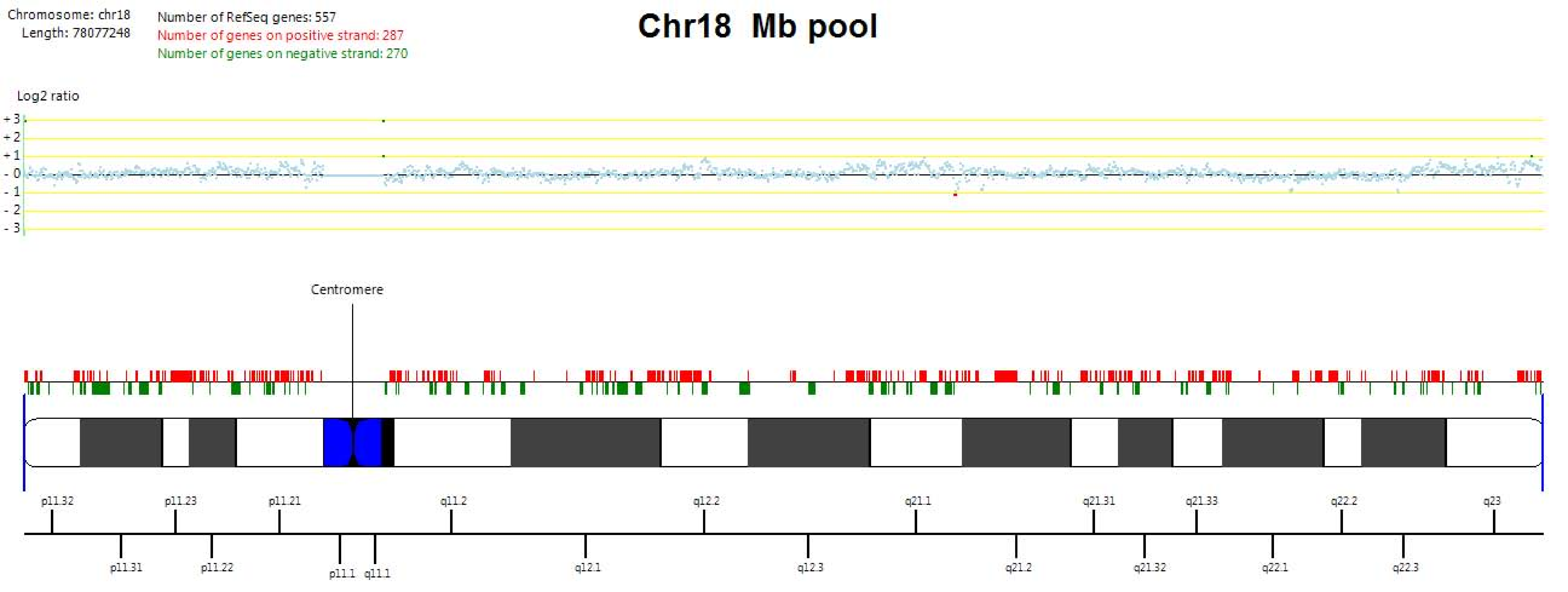
Chromosome: chr18
Length: 78077248
Number of RefSeq genes: 557
Number of genes on positive strand: 287
Number of genes on negative strand: 270
Chr18 Mb pool
Log2 ratio
+3
+2
+1
-0
-1
-2
-3
Centromere
p11.32
p11.23
p11.21
q11.2
q12.2
q21.1
q21.31
q21.33
q22.2
q23
p11.31
p11.22
p11.1
q11.1
q12.1
q12.3
q21.2
q21.32
q22.1
q22.3

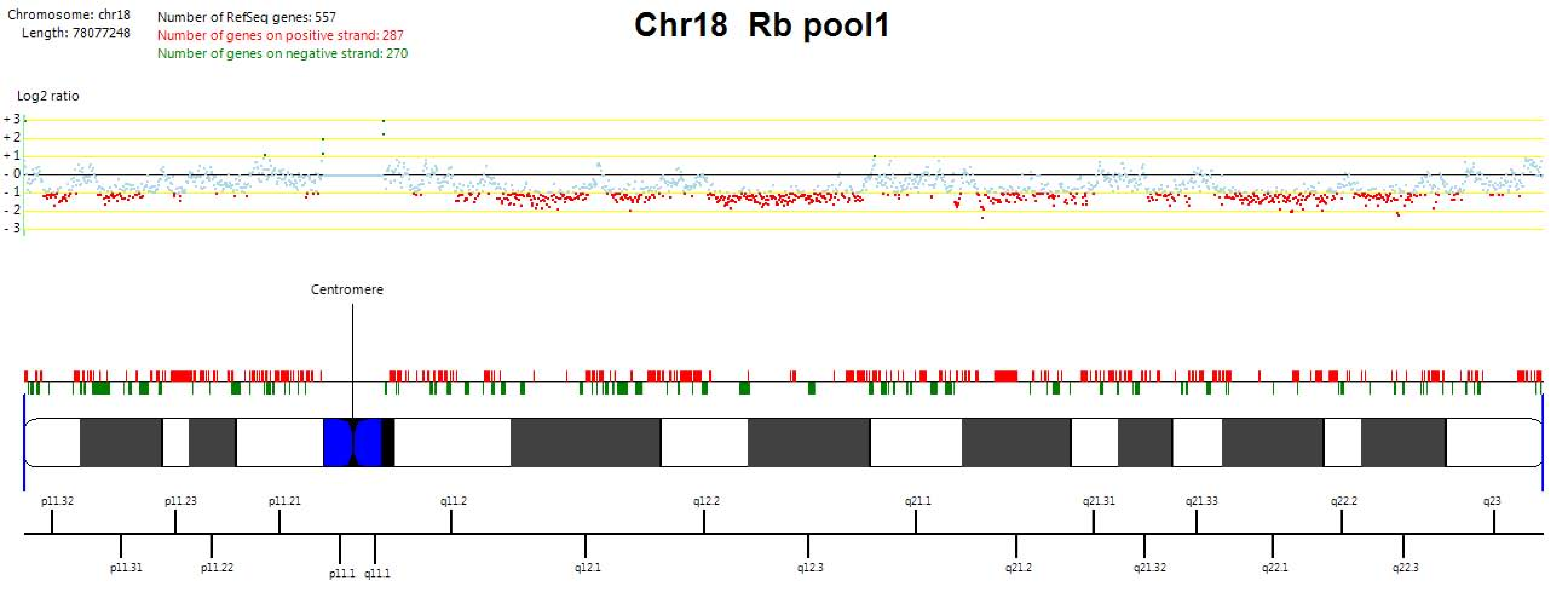

Chromosome: chr18
Length: 78077248
Number of RefSeq genes: 557
Number of genes on positive strand: 287
Number of genes on negative strand: 270
Chr18 Rb pool1
Log2 ratio
+3
+2
+1
- 0
- 1
- 2
- 3
Centromere
p11.32
p11.23
p11.21
q11.2
q12.2
q21.1
q21.31
q21.33
q22.2
q23
p11.31
p11.22
p11.1
q11.1
q12.1
q12.3
q21.2
q21.32
q22.1
q22.3

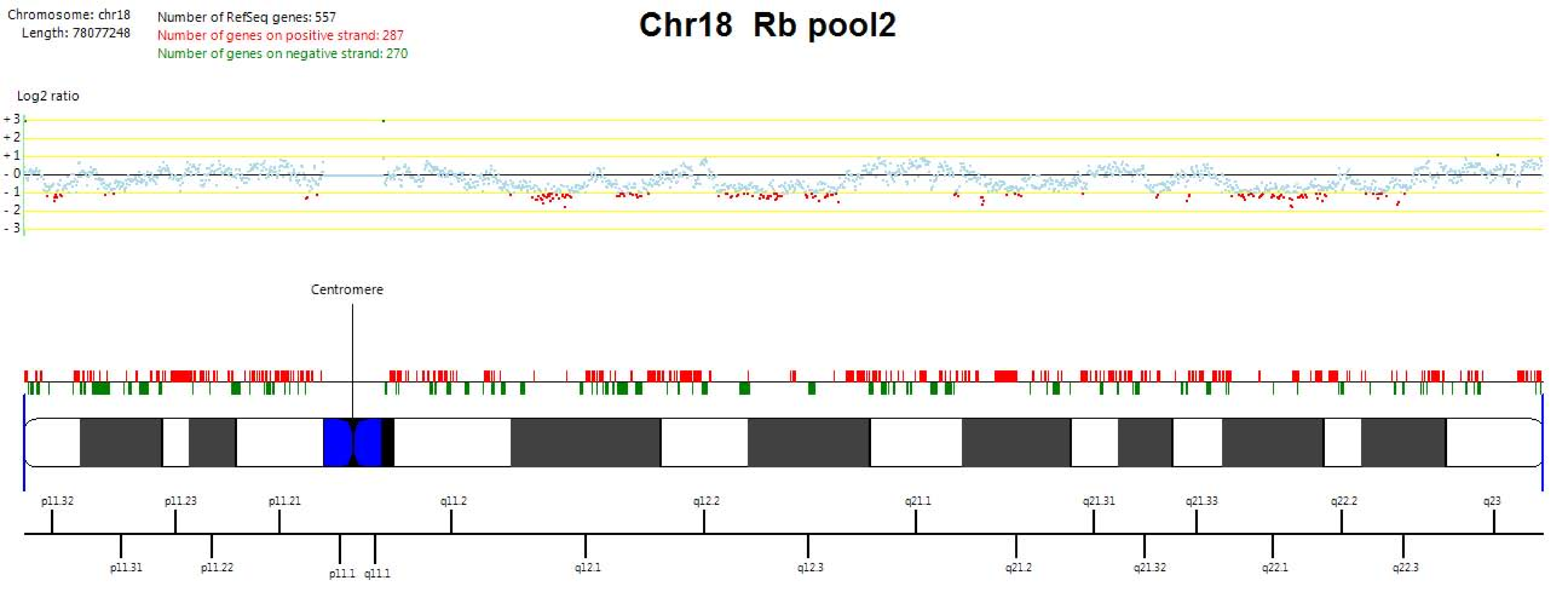

Chromosome: chr18
Length: 78077248
Number of RefSeq genes: 557
Number of genes on positive strand: 287
Number of genes on negative strand: 270
Chr18 Rb pool2
Log2 ratio
+3
+2
+1
-0
-1
-2
-3
Centromere
p11.32
p11.23
p11.21
q11.2
q12.2
q21.1
q21.31
q21.33
q22.2
q23
p11.31
p11.22
p11.1
q11.1
q12.1
q12.3
q21.2
q21.32
q22.1
q22.3

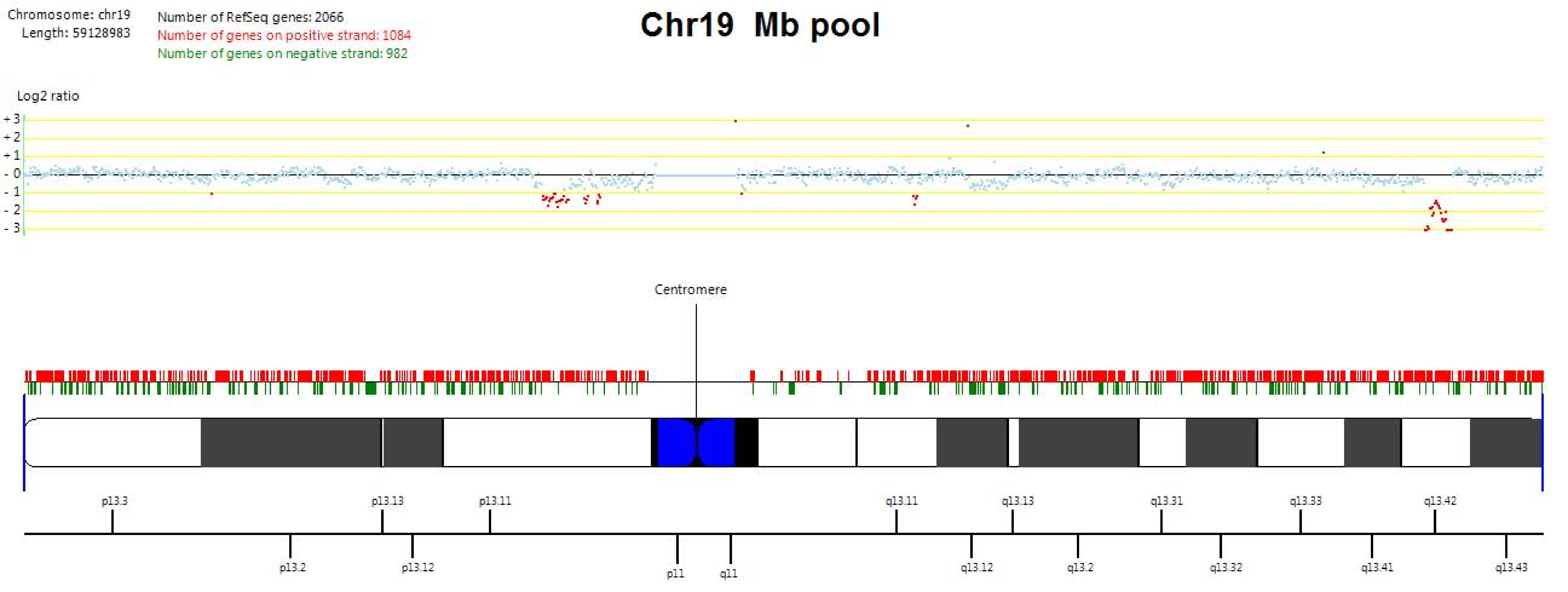
Chromosome: chr19
Length: 59128983
Number of RefSeq genes: 2066
Number of genes on positive strand: 1084
Number of genes on negative strand: 982
Chr19 Mb pool
Log2 ratio
+3
+2
+1
-0
-1
-2
-3
Centromere
p13.3
p13.2
p13.13
p13.12
p13.11
p11
q11
q13.11
q13.12
q13.13
q13.2
q13.31
q13.32
q13.33
q13.41
q13.42
q13.43

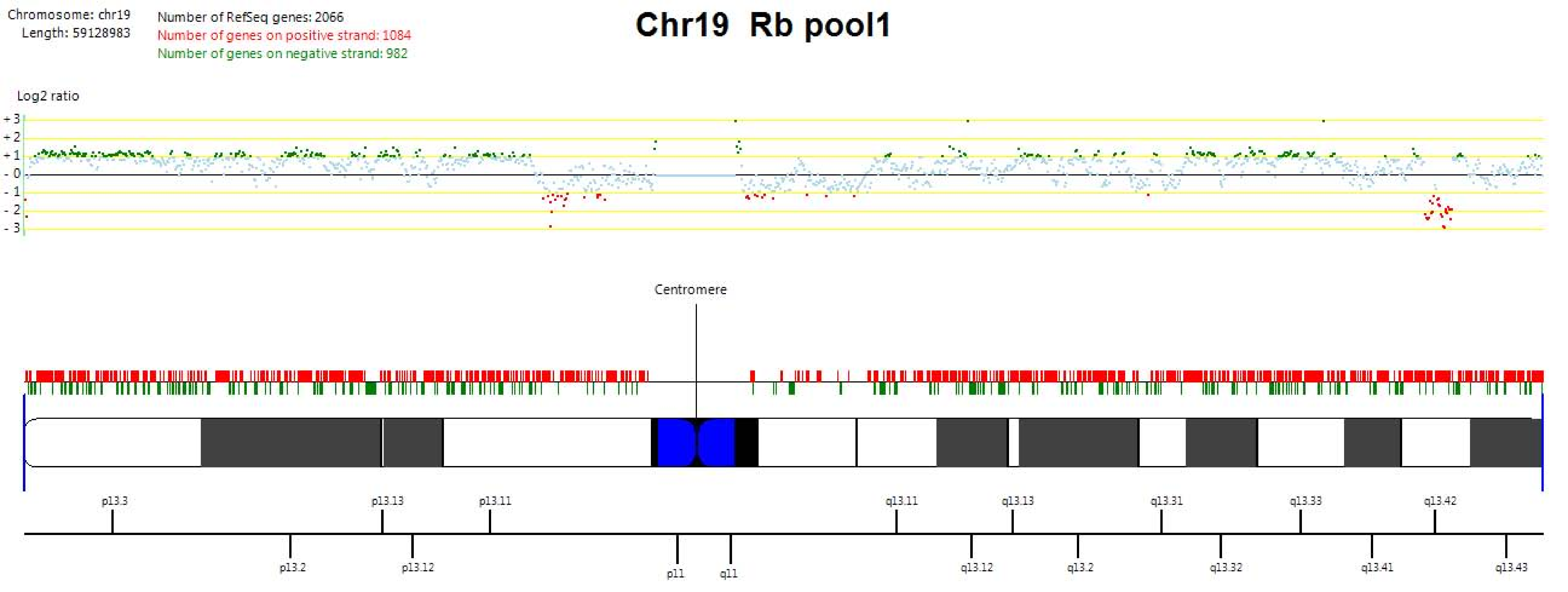

Chromosome: chr19
Length: 59128983
Number of RefSeq genes: 2066
Number of genes on positive strand: 1084
Number of genes on negative strand: 982
Chr19 Rb pool1
Log2 ratio
+3
+2
+1
-0
-1
-2
-3
Centromere
p13.3
p13.2
p13.13
p13.12
p13.11
p11
q11
q13.11
q13.12
q13.13
q13.2
q13.31
q13.32
q13.33
q13.41
q13.42
q13.43

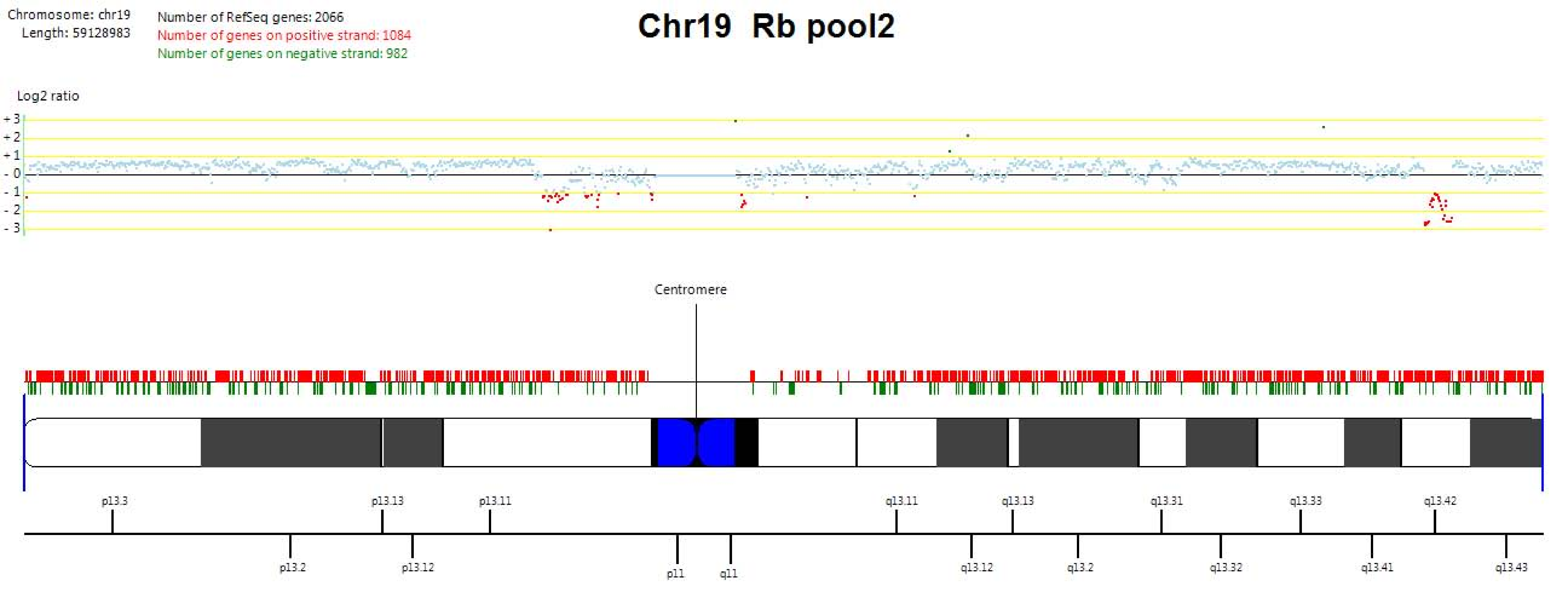

Chromosome: chr19
Length: 59128983
Number of RefSeq genes: 2066
Number of genes on positive strand: 1084
Number of genes on negative strand: 982
Chr19 Rb pool2
Log2 ratio
+3
+2
+1
-0
-1
-2
-3
Centromere
p13.3
p13.2
p13.13
p13.12
p13.11
p11
q11
q13.11
q13.12
q13.13
q13.2
q13.31
q13.32
q13.33
q13.41
q13.42
q13.43

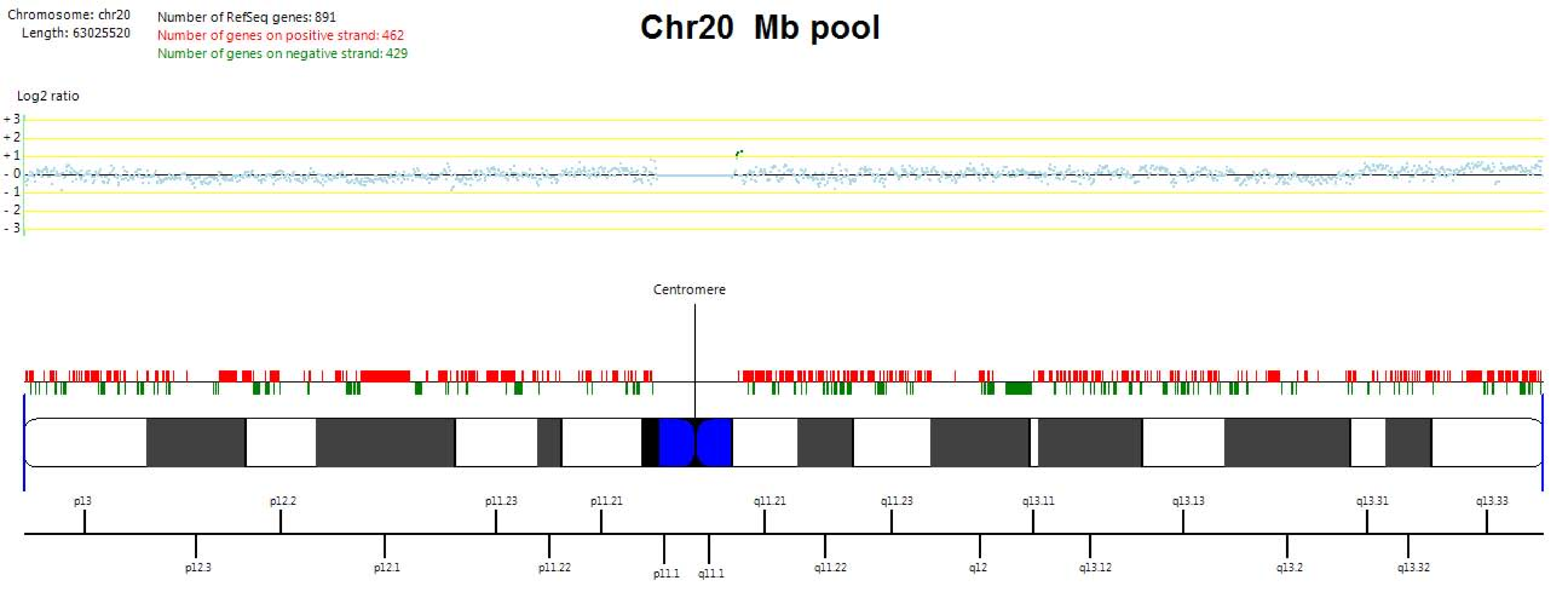

Chromosome: chr20
Length: 63025520
Number of RefSeq genes: 891
Number of genes on positive strand: 462
Number of genes on negative strand: 429
Chr20 Mb pool
Log2 ratio
+3
+2
+1
0
-1
-2
-3
Centromere
p13
p12.3
p12.2
p12.1
p11.23
p11.22
p11.21
p11.1
q11.1
q11.21
q11.22
q11.23
q12
q13.11
q13.12
q13.13
q13.2
q13.31
q13.32
q13.33

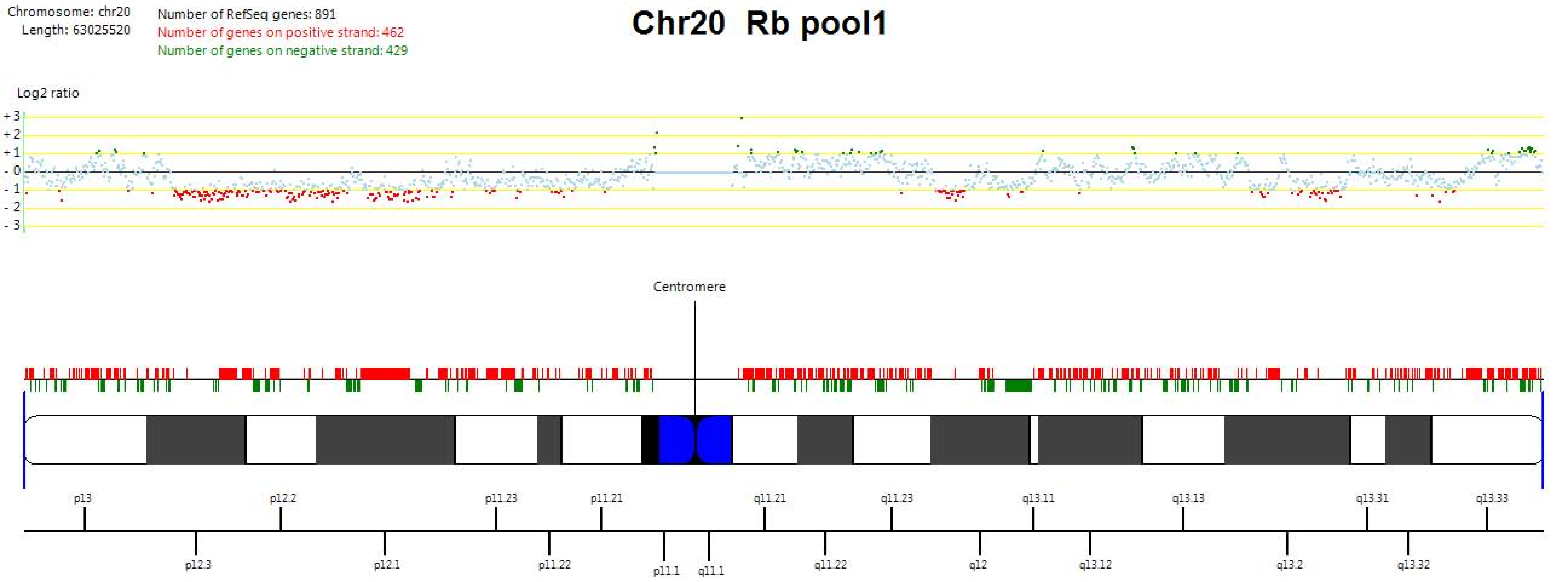

Chromosome: chr20
Length: 63025520
Number of RefSeq genes: 891
Number of genes on positive strand: 462
Number of genes on negative strand: 429
Chr20 Rb pool1
Log2 ratio
+3
+2
+1
-0
-1
-2
-3
Centromere
p13
p12.2
p11.23
p11.21
q11.21
q11.23
q13.11
q13.13
q13.31
q13.33
p12.3
p12.1
p11.22
p11.1
q11.1
q11.22
q12
q13.12
q13.2
q13.32

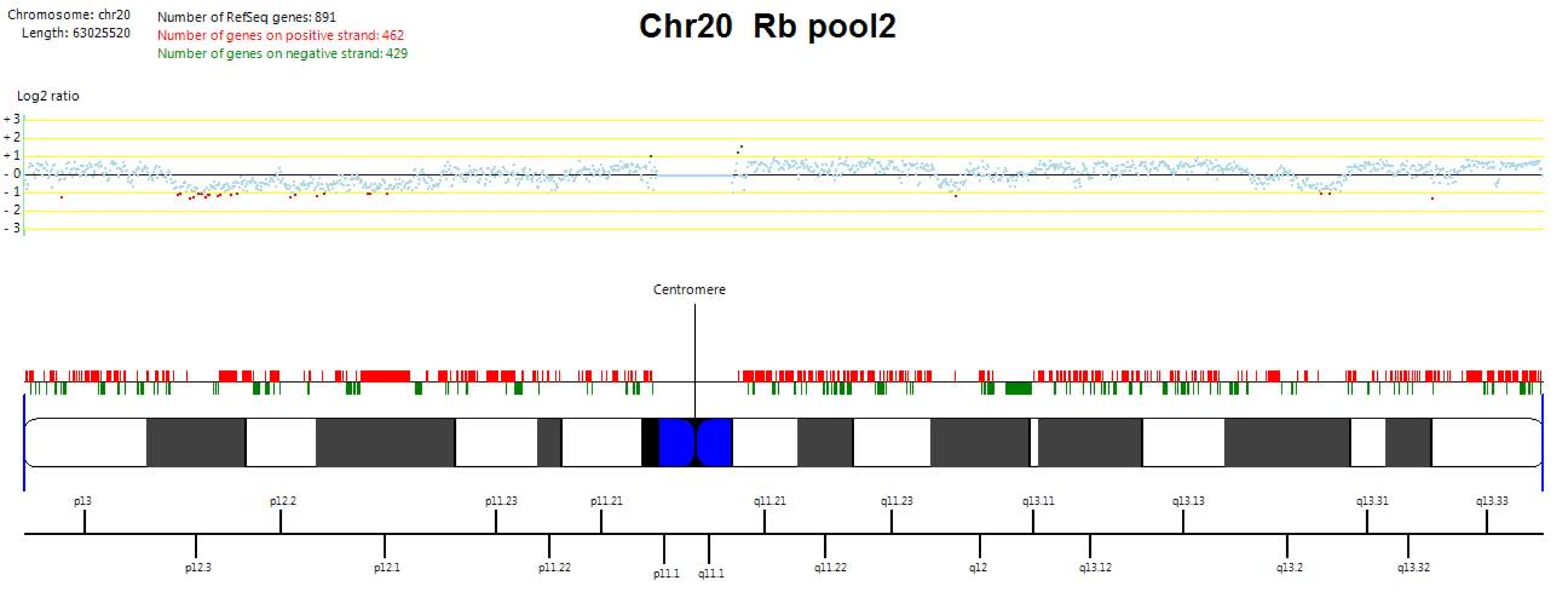

Chromosome: chr20
Length: 63025520
Number of RefSeq genes: 891
Number of genes on positive strand: 462
Number of genes on negative strand: 429
Chr20 Rb pool2
Log2 ratio
+3
+2
+1
-0
-1
-2
-3
Centromere
p13
p12.3
p12.2
p12.1
p11.23
p11.22
p11.21
p11.1
q11.1
q11.21
q11.22
q11.23
q12
q13.11
q13.12
q13.13
q13.2
q13.31
q13.32
q13.33

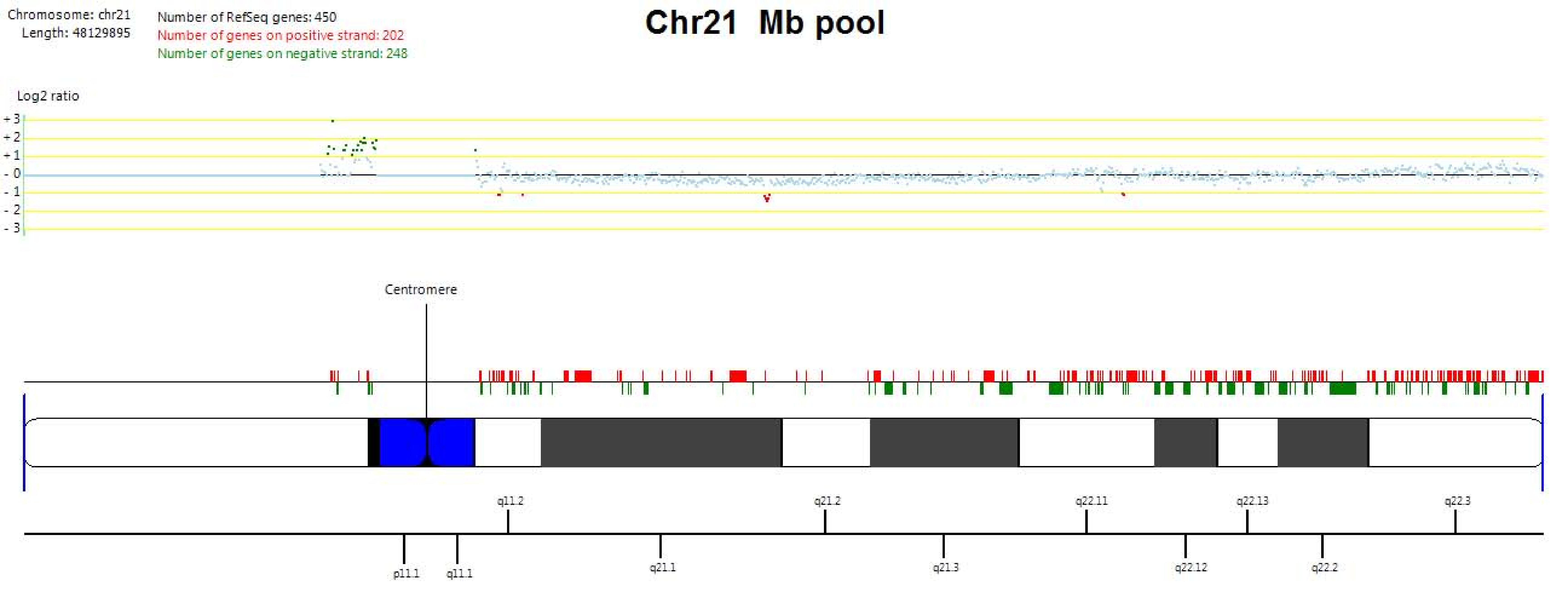
Chromosome: chr21
Length: 48129895
Number of RefSeq genes: 450
Number of genes on positive strand: 202
Number of genes on negative strand: 248
Chr21 Mb pool
Log2 ratio
+3
+2
+1
-0
-1
-2
-3
Centromere
q11.2
q21.2
q22.11
q22.13
q22.3
p11.1
q11.1
q21.1
q21.3
q22.12
q22.2

Chr21 Rb pool1

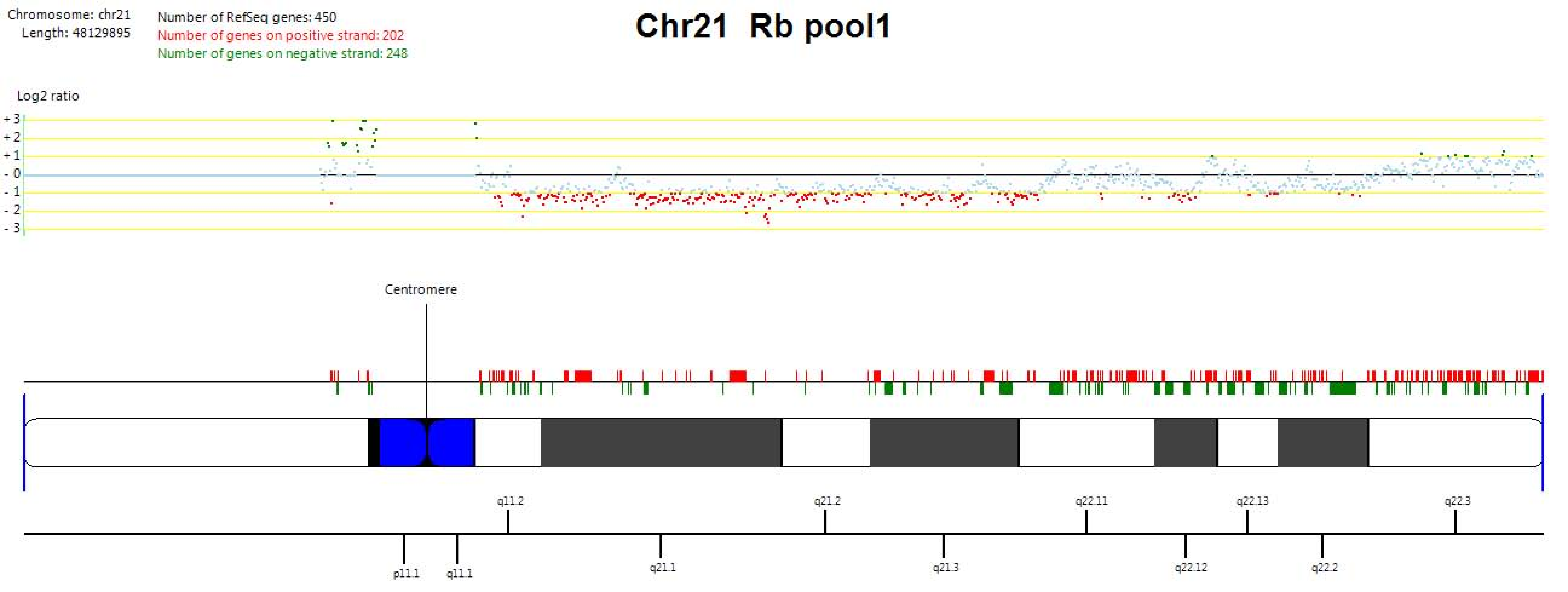

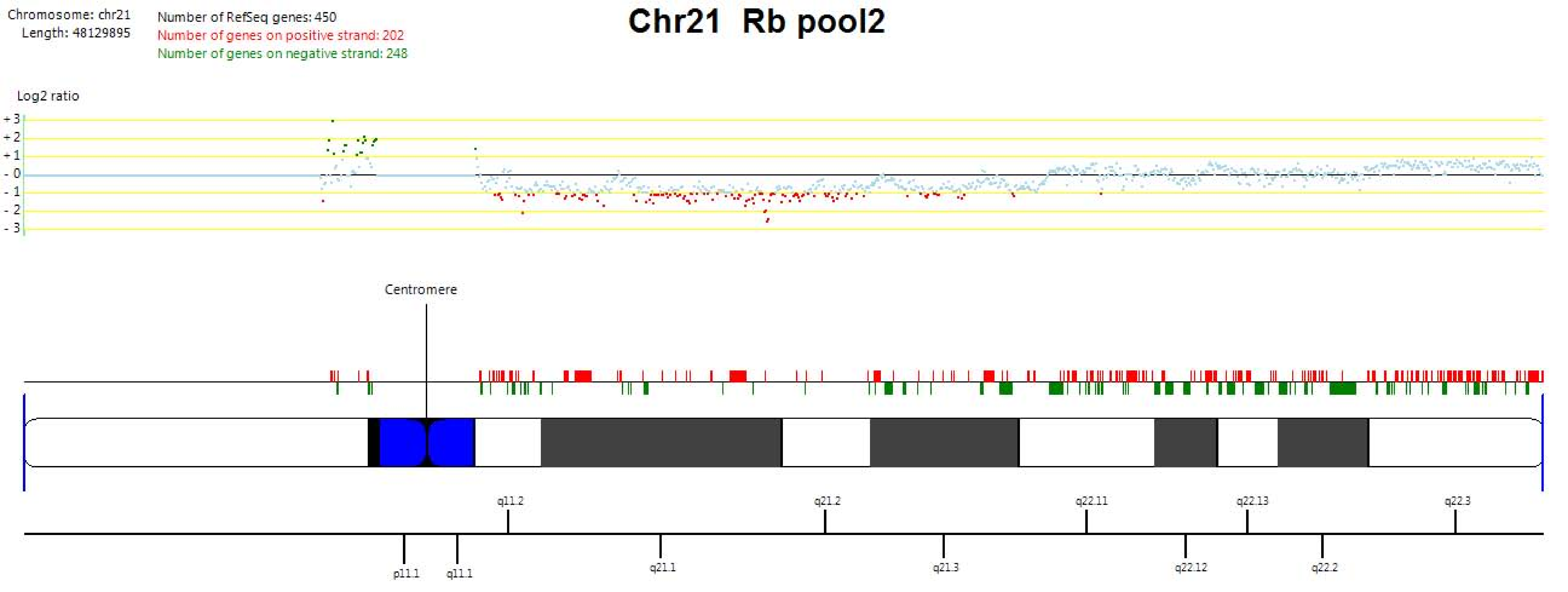
Chromosome: chr21
Length: 48129895
Number of RefSeq genes: 450
Number of genes on positive strand: 202
Number of genes on negative strand: 248
Chr21 Rb pool2
Log2 ratio
+3
+2
+1
0
-1
-2
-3
Centromere
q11.2
q21.2
q22.11
q22.13
q22.3
p11.1
q11.1
q21.1
q21.3
q22.12
q22.2

Chromosome: chr22
Length: 51304566

Number of RefSeq genes: 855
Number of genes on positive strand: 465
Number of genes on negative strand: 390

Chr22 Mb pool

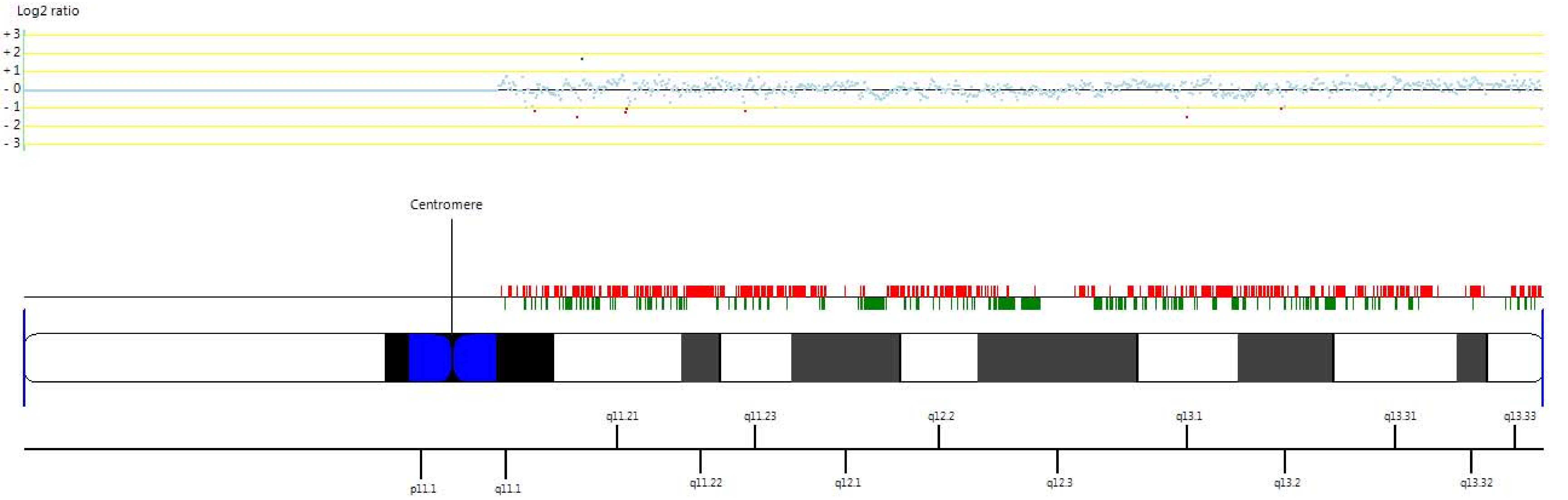

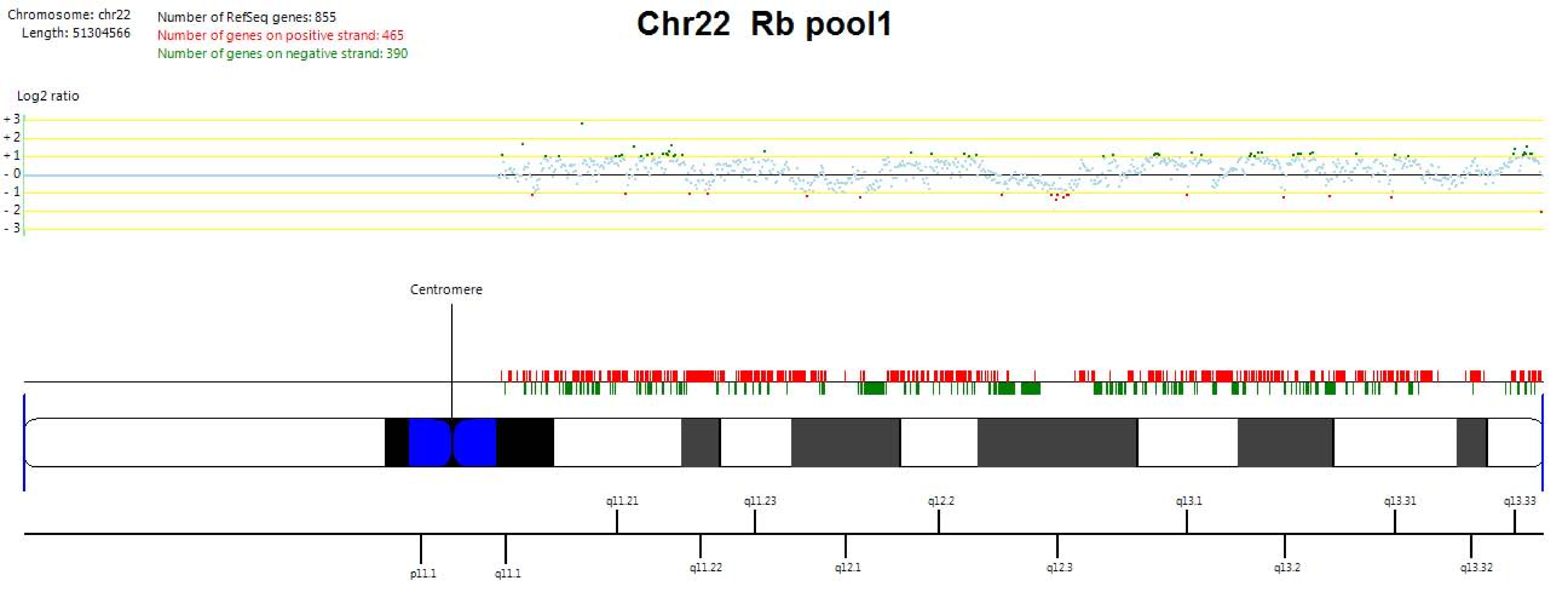

Chromosome: chr22
Length: 51304566
Number of RefSeq genes: 855
Number of genes on positive strand: 465
Number of genes on negative strand: 390
Chr22 Rb pool1
Log2 ratio
+3
+2
+1
-0
-1
-2
-3
Centromere
q11.21
q11.23
q12.2
q13.1
q13.31
q13.33
p11.1
q11.1
q11.22
q12.1
q12.3
q13.2
q13.32

Chr22 Rb pool2

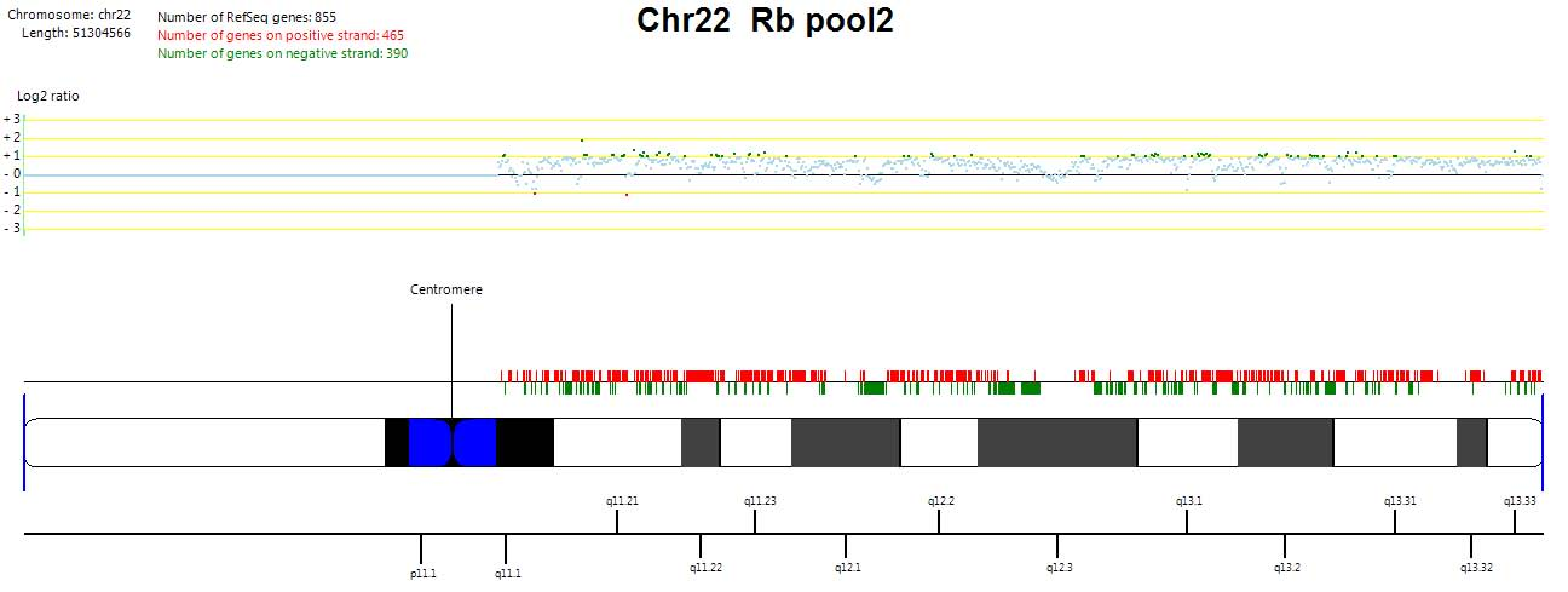

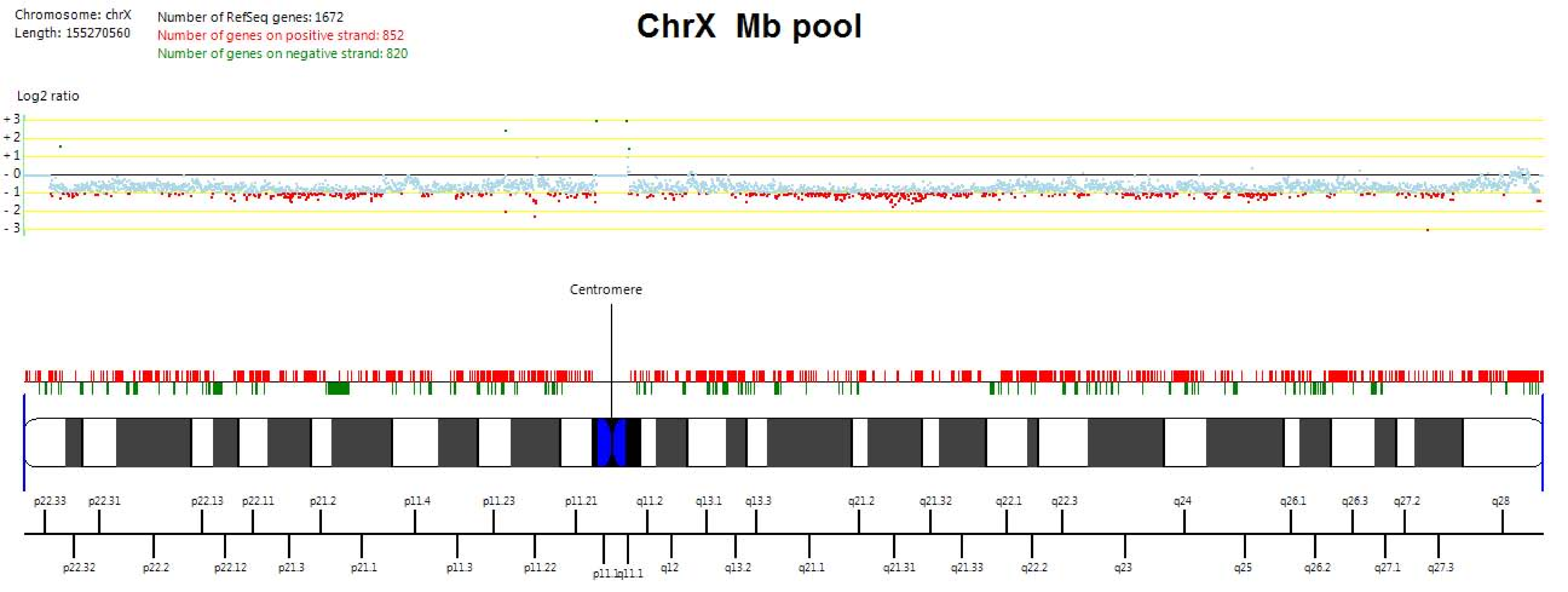
Chromosome: chrX
Length: 155270560
Number of RefSeq genes: 1672
Number of genes on positive strand: 852
Number of genes on negative strand: 820
ChrX Mb pool
Log2 ratio
+3
+2
+1
- 0
- 1
- 2
- 3
Centromere
p22.33
p22.31
p22.13
p22.11
p21.2
p11.4
p11.23
p11.21
q11.2
q13.1
q13.3
q21.2
q21.32
q22.1
q22.3
q24
q26.1
q26.3
q27.2
q28
p22.32
p22.2
p22.12
p21.3
p21.1
p11.3
p11.22
p11.1
q11.1
q12
q13.2
q21.1
q21.31
q21.33
q22.2
q23
q25
q26.2
q27.1
q27.3

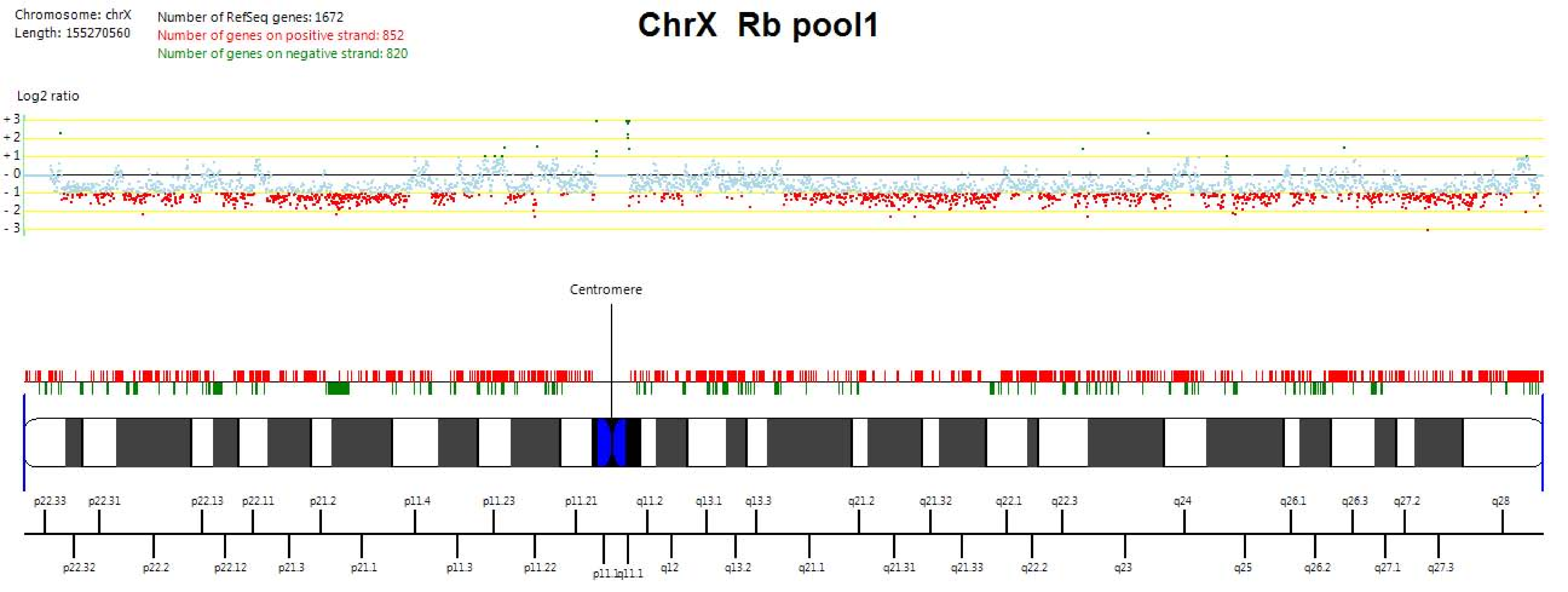
Chromosome: chrX
Length: 155270560
Number of RefSeq genes: 1672
Number of genes on positive strand: 852
Number of genes on negative strand: 820
ChrX Rb pool1
Log2 ratio
+3
+2
+1
-0
-1
-2
-3
Centromere
p22.33
p22.31
p22.13
p22.11
p21.2
p11.4
p11.23
p11.21
q11.2
q13.1
q13.3
q21.2
q21.32
q22.1
q22.3
q24
q26.1
q26.3
q27.2
q28
p22.32
p22.2
p22.12
p21.3
p21.1
p11.3
p11.22
p11.1
q11.1
q12
q13.2
q21.1
q21.31
q21.33
q22.2
q23
q25
q26.2
q27.1
q27.3

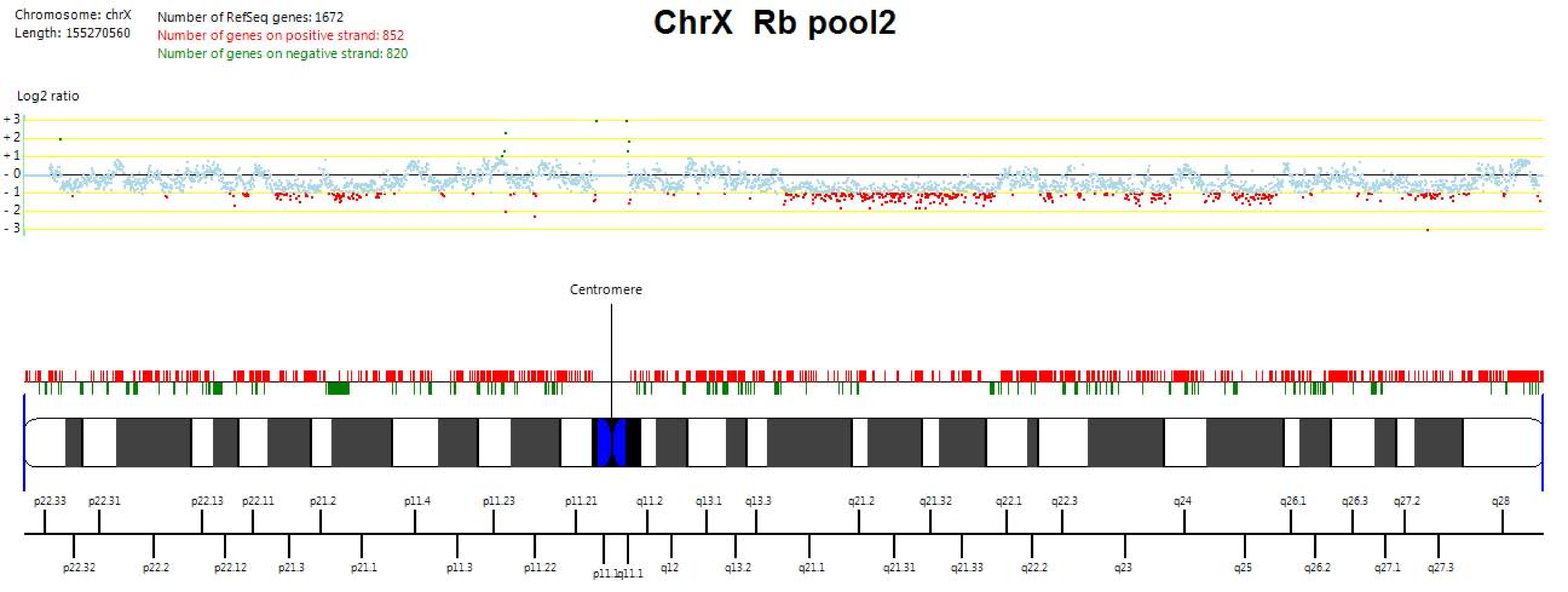

Chromosome: chrX
Length: 155270560
Number of RefSeq genes: 1672
Number of genes on positive strand: 852
Number of genes on negative strand: 820
ChrX Rb pool2
Log2 ratio
+3
+2
+1
- 0
- 1
- 2
- 3
Centromere
p22.33
p22.31
p22.13
p22.11
p21.2
p11.4
p11.23
p11.21
q11.2
q13.1
q13.3
q21.2
q21.32
q22.1
q22.3
q24
q26.1
q26.3
q27.2
q28
p22.32
p22.2
p22.12
p21.3
p21.1
p11.3
p11.22
p11.1
q11.1
q12
q13.2
q21.1
q21.31
q21.33
q22.2
q23
q25
q26.2
q27.1
q27.3

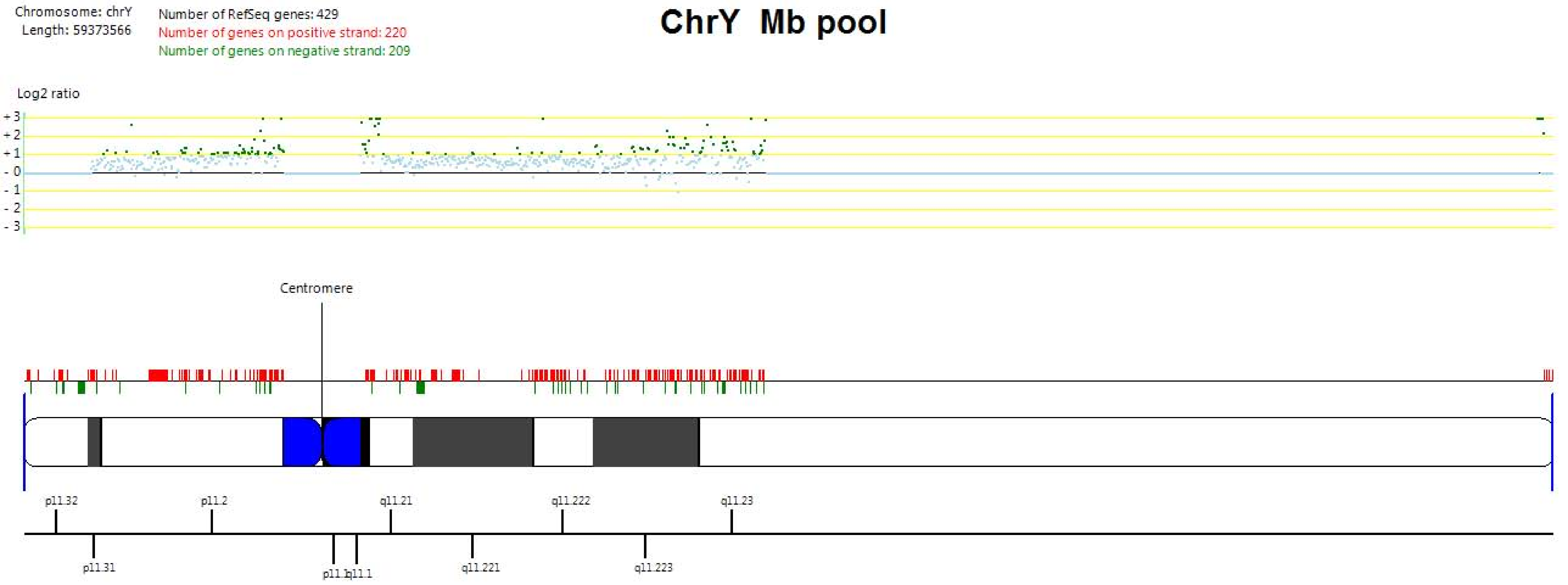
Chromosome: chrY
Length: 59373566
Number of RefSeq genes: 429
Number of genes on positive strand: 220
Number of genes on negative strand: 209
ChrY Mb pool
Log2 ratio
+3
+2
+1
- 0
- 1
- 2
- 3
Centromere
p11.32
p11.31
p11.2
p11.1
q11.1
q11.21
q11.221
q11.222
q11.223
q11.23

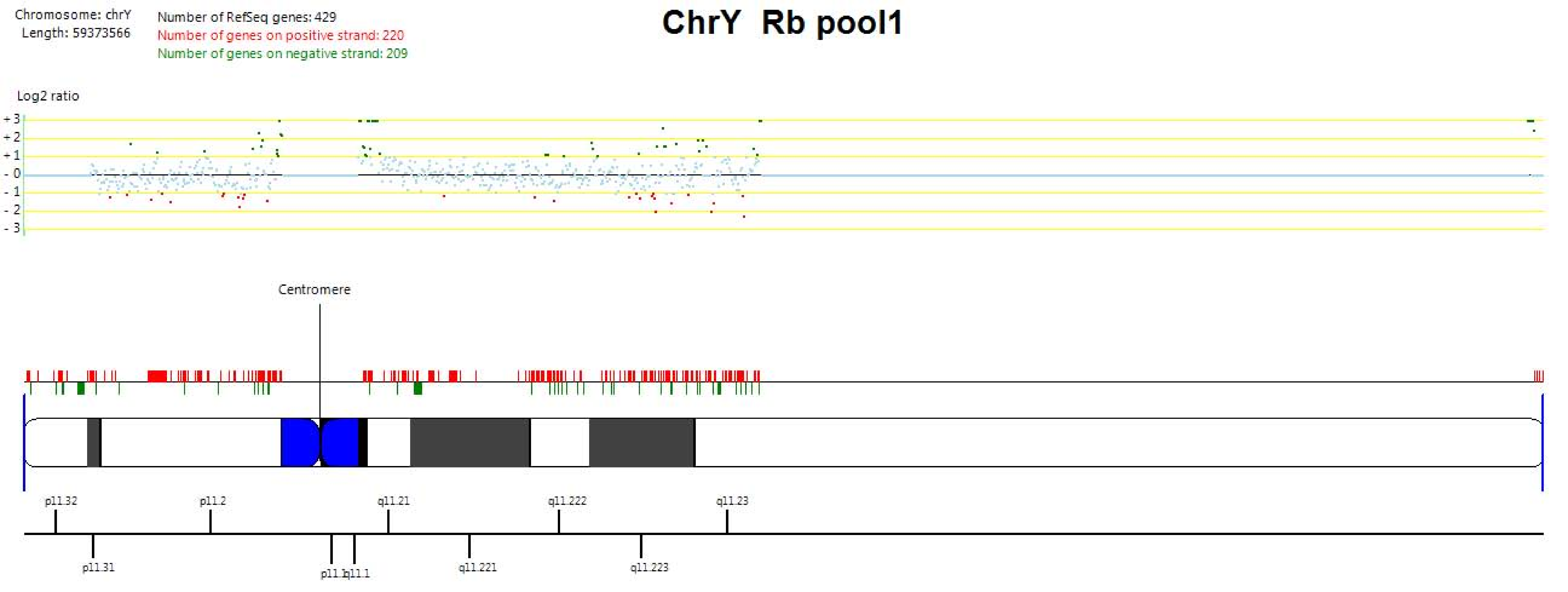

Chromosome: chrY
Length: 59373566
Number of RefSeq genes: 429
Number of genes on positive strand: 220
Number of genes on negative strand: 209
ChrY Rb pool1
Log2 ratio
+3
+2
+1
- 0
- 1
- 2
- 3
Centromere
p11.32
p11.2
q11.21
q11.222
q11.23
p11.31
p11.1
p11.1
q11.221
q11.223

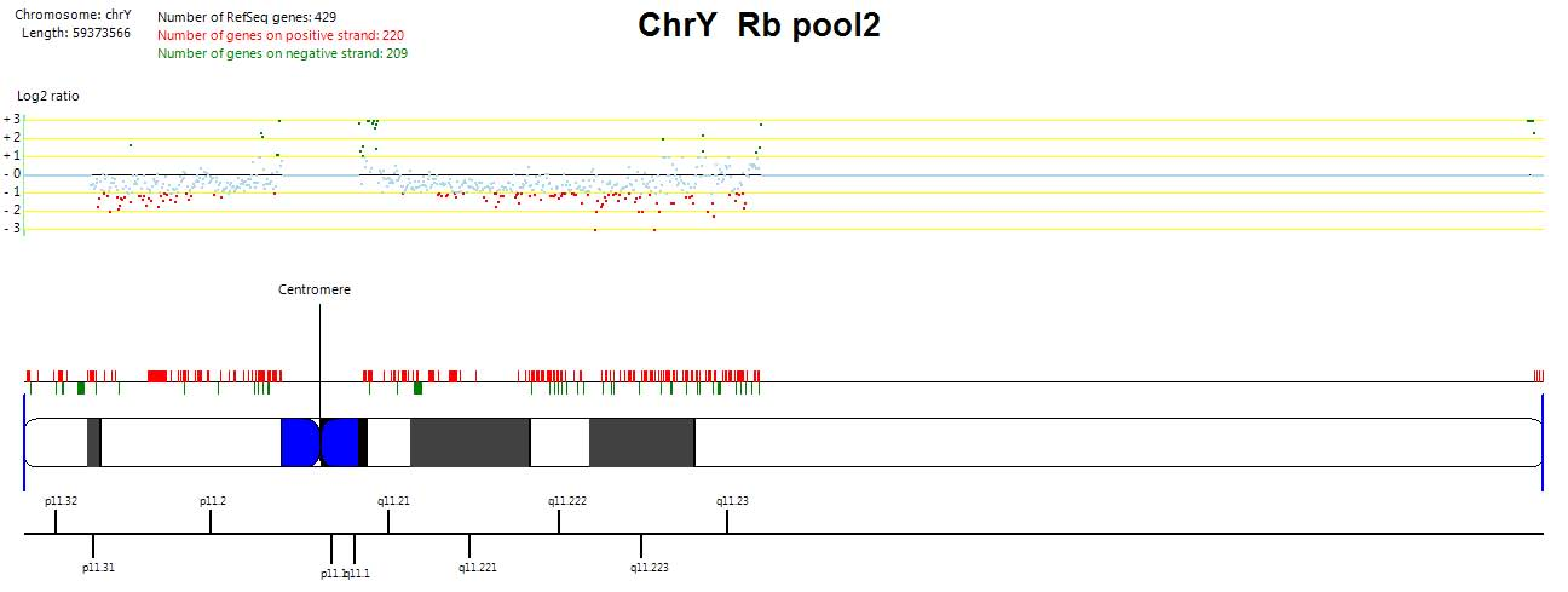
Chromosome: chrY
Length: 59373566
Number of RefSeq genes: 429
Number of genes on positive strand: 220
Number of genes on negative strand: 209
ChrY Rb pool2
Log2 ratio
+3
+2
+1
-0
-1
-2
-3
Centromere
p11.32
p11.2
q11.21
q11.222
q11.23
p11.31
q11.221
q11.223
